# sRNA Profiling Combined With Gene Function Analysis Reveals a Lack of Evidence for Cross-Kingdom RNAi in the Wheat – *Zymoseptoria tritici* Pathosystem

**DOI:** 10.3389/fpls.2019.00892

**Published:** 2019-07-04

**Authors:** Graeme J. Kettles, Bernhard J. Hofinger, Pingsha Hu, Carlos Bayon, Jason J. Rudd, Dirk Balmer, Mikael Courbot, Kim E. Hammond-Kosack, Gabriel Scalliet, Kostya Kanyuka

**Affiliations:** ^1^Biointeractions and Crop Protection, Rothamsted Research, Harpenden, United Kingdom; ^2^Syngenta Biotechnology, Inc., Research Triangle Park, NC, United States; ^3^Syngenta Crop Protection AG, Stein, Switzerland

**Keywords:** cross-kingdom RNAi, RNA interference, small RNA, host-induced gene silencing, septoria tritici blotch, *Zymoseptoria tritici*, *Triticum aestivum*, effectors

## Abstract

Cross-kingdom small RNA (sRNA) silencing has recently emerged as a mechanism facilitating fungal colonization and disease development. Here we characterized RNAi pathways in *Zymoseptoria tritici*, a major fungal pathogen of wheat, and assessed their contribution to pathogenesis. Computational analysis of fungal sRNA and host mRNA sequencing datasets was used to define the global sRNA populations in *Z. tritici* and predict their mRNA targets in wheat. 389 *in planta*-induced sRNA loci were identified. sRNAs generated from some of these loci were predicted to target wheat mRNAs including those potentially involved in pathogen defense. However, molecular approaches failed to validate targeting of selected wheat mRNAs by fungal sRNAs. Mutant strains of *Z. tritici* carrying deletions of genes encoding key components of RNAi such as Dicer-like (DCL) and Argonaute (AGO) proteins were generated, and virulence bioassays suggested that these are dispensable for full infection of wheat. Nonetheless, our results did suggest the existence of non-canonical DCL-independent pathway(s) for sRNA biogenesis in *Z. tritici*. dsRNA targeting essential fungal genes applied *in vitro* or generated from an RNA virus vector *in planta* in a procedure known as HIGS (Host-Induced Gene Silencing) was ineffective in preventing *Z. tritici* growth or disease. We also demonstrated that *Z. tritici* is incapable of dsRNA uptake. Collectively, our data suggest that RNAi approaches for gene function analyses in this fungal species and potentially also as a control measure may not be as effective as has been demonstrated for some other plant pathogenic fungi.

## Introduction

In plants, RNA silencing also known as RNAi (RNA interference) describes a collection of related biochemical pathways where numerous small RNA (sRNA) species, typically 21–24 nucleotides (nt) in length, e.g., miRNA (microRNA), siRNA (small interfering RNA), tasiRNA (*trans-*acting siRNA), natsiRNA (natural antisense siRNA) modify the expression of target mRNA molecules through classical Watson-Crick base-pairing (Ghildiyal and Zamore, 2009; Vickers et al., 2015). Generation of sRNA is achieved through the action of a Dicer-like (DCL) RNA helicase with an RNase III activity acting on double-stranded RNA (dsRNA) molecules synthesized by an RNA-dependent RNA polymerase (RdRp) or single-stranded RNAs folded into a secondary hairpin-like structure (Xie et al., 2004; Gasciolli et al., 2005). Once produced, sRNAs exert their activity on targets bearing sufficient complementarity through the activity of an RNA-induced Silencing Complex (RISC) containing an Argonaute (AGO) class endonuclease, which binds sRNAs, in its core (Vazquez et al., 2010). Target mRNAs are typically negatively regulated either through cleavage or translational inhibition (Vazquez et al., 2010). RNAi pathways are deeply integrated in plant immune processes and are involved in defense responses against viral, bacterial and fungal pathogens, and invertebrate pests (Hamilton and Baulcombe, 1999; Navarro et al., 2006; Pandey and Baldwin, 2007; Ellendorff et al., 2009; Kettles et al., 2013). Additionally, they orchestrate numerous developmental processes, responses to changes in the abiotic environment, and DNA methylation and heterochromatin formation (Fujii et al., 2005; Matzke et al., 2007; Sunkar et al., 2007). In filamentous fungi, a diverse range of RNAi pathways dependent on DCL, AGO and RdRp have also been identified and shown to be involved in protection against invasive nucleic acids, e.g., those generated from retrotransposons (Nolan et al., 2005; Segers et al., 2007), control of heterochromatin formation (Volpe et al., 2002), and in the meiotic silencing by unpaired DNA pathway (Chang et al., 2012). Intriguingly, RNAi pathways have also been significantly modified or lost in some fungal lineages thus suggesting that in some circumstances loss of RNAi may confer significant evolutionary advantage (Billmyre et al., 2013; Nicolás et al., 2013).

Recent observations, particularly using the *Arabidopsis thaliana* – *Botrytis cinerea* pathosystem, have suggested that natural cross-kingdom gene silencing can occur in some plant-pathogen interactions (Weiberg et al., 2013; Wang et al., 2016). It has been demonstrated that *B. cinerea* expresses numerous sRNAs during infection of Arabidopsis, and that some of these sRNAs can inhibit accumulation of certain plant defense-related transcripts apparently facilitating fungal colonization and disease development (Weiberg et al., 2013; Cai et al., 2018). Therefore, it appears that some fungal sRNAs can function in a manner analogous to the pathogen effector proteins. Furthermore, the discovery that sRNAs can transit between plant and fungal cells to modify gene expression in the recipient cell (Baulcombe, 2015), opens the possibility for novel crop protection strategies based on RNAi. One such strategy, known as host-induced gene silencing (HIGS), typically involves generation of transgenic plants expressing long dsRNAs or hairpin RNAs exhibiting high sequence homology to the essential pathogen mRNAs. Uptake of sRNAs generated *in planta* from these dsRNA species by the pathogen induces silencing of the target genes and ultimately suppression of the disease. HIGS has been demonstrated in several fungal and oomycete pathosystems (Nowara et al., 2010; Koch et al., 2013; Ghag et al., 2014; Cheng et al., 2015; Chen et al., 2016; Qi et al., 2018; Song and Thomma, 2018). To overcome potential difficulties with generating transgenic plant material and the associated GMO safety aspects, a new spray-induced gene silencing (SIGS) strategy involving exogenous application of synthetic dsRNA or siRNA molecules (“RNA fungicides”) to the plants for the control of fungal pathogens has recently been described (Koch et al., 2016; Wang et al., 2016; Machado et al., 2018; McLoughlin et al., 2018).

The ascomycete fungus *Zymoseptoria tritici* is the causative agent of septoria tritici blotch (STB) disease and is the major threat to bread and pasta wheat (*Triticum aestivum* and *Triticum durum*) production globally (Dean et al., 2012). *Z. tritici* is a hemibiotrophic foliar pathogen, which invades leaf tissue through natural openings such as stomata. *Z. tritici* remains exclusively apoplastic through its infection cycle, which is characterized by an extended symptomless infection phase (10–14 days) followed by the rapid transition to necrotrophy (Kema et al., 1996; Keon et al., 2007). Considerable progress has been made in understanding the infection biology of *Z. tritici*, including the role of fungal secreted proteins in facilitating or hindering plant colonization (Marshall et al., 2011; Kettles and Kanyuka, 2016; Fones et al., 2017; Kettles et al., 2017; Zhong et al., 2017; Kema et al., 2018; Meile et al., 2018). Whilst over twenty wheat genes for resistance to *Z. tritici* have been identified and characterized genetically, only one gene (*Stb6*) has been cloned so far (Saintenac et al., 2018).

Recent developments in the fledgling field of cross-kingdom RNAi prompted us to ask whether this phenomenon was involved in the colonization of wheat by *Z. tritici*, an exclusively extracellularly (apoplastically) dwelling pathogen. Here we report the identification and characterization of the sRNA populations generated by this fungal species during wheat leaf infection, predict putative wheat transcripts that may be subject to cross-kingdom RNAi and carry out validation of a subset of such interactions experimentally. The role of RNAi pathways in the infection biology of *Z. tritici* was assessed through the generation of targeted single gene deletion mutants. We also assessed whether *Z. tritici* has a capacity to uptake exogenously applied long dsRNA and sRNA and explored HIGS and *in vitro* RNAi as alternative approaches for characterizing fungal gene function and potentially also for control of this economically important fungal pathogen.

## Materials and Methods

### Plant and Fungal Material for Small RNA Sequencing

The *Z. tritici* isolate IPO323 and wheat (*T. aestivum*) cv. Bobwhite, which is fully susceptible to this and many other *Z. tritici* isolates, were used in all experiments. Fungal Czapek-Dox Broth (CDB) cultures were propagated in shake flasks at 220 rpm and 15°C for 4 d and then harvested via filtration. Plant inoculation experiments were done as described previously (Rudd et al., 2015) using a suspension of 1 × 10^7^ spores⋅mL^−1^ in water supplemented with 0.1% (v/v) Silwet L-77. Mock inoculations of plants were made using a 0.1% (v/v) Silwet L-77 water solution. Each biological replicate plant sample for RNA isolation was made up of five 6-cm long leaf segments each collected from a separate individual mock- or *Z. tritici-*inoculated plant randomly distributed in a single temperature-controlled glasshouse. Leaves and fungal *in vitro* samples were immediately frozen in liquid nitrogen and stored at −80°C before used for RNA purification.

### RNA Sequencing and Bioinformatics Analysis

Wheat cv. Bobwhite leaf tissue samples mock-inoculated and those inoculated with *Z. tritici* isolate IPO323 were collected at 4 dpi (asymptomatic stage), 9 dpi (first signs of host cell death), 13 dpi (extensive host cell death and a few fungal pycnidia initials visible), and 21 dpi (end of infection with numerous pycnidia visible), respectively. Healthy, untreated 17 days old wheat cv. Bobwhite leaf tissue was also collected to serve as an additional control. Samples were used for RNA extraction with the ZR RNA MiniPrep kit (Zymo Research). Concentration, quality and integrity of each RNA preparation was verified using NanoDrop Spectrophotometer (Thermo Fisher Scientific), Qubit 2.0 Fluorometer (Thermo Fisher Scientific), and 2100 Bioanalyzer (Agilent). sRNA libraries were constructed using the TruSeq small-RNA Library Preparation kit (Illumina) and sequenced as 50 cycle single end reads on a HiSeq 2000 (Illumina) by GENEWIZ. For the RNA samples that have been produced and sequenced there were 3 independent replicates for each timepoint and condition (inoculated, mock inoculated, and healthy untreated). The sequencing data was deposited to the European Nucleotide Archive under the study PRJEB28454. The raw reads were trimmed using Trim Galore!^1^ to remove the adapter sequences with a minimum length cut-off 17 nt and trimmed fastq sequences analyzed using FastQC^2^.

The trimmed reads were aligned to the genomes of *Z. tritici* IPO323^3^ and wheat cv. Chinese Spring^4^ using Bowtie 2 (Langmead and Salzberg, 2012). End-to-end model was used in Bowtie 2 and the score-min was set as L, 0, −0.1. All other parameters were default. Mapped reads from all the samples were merged together for sRNA loci discovery using SiLoCo (UEA sRNA toolkit^5^; Moxon et al., 2008; Stocks et al., 2012) with the following key parameters: maximum gap 50 nt, maximum sRNA size 30 nt, minimum sRNA size 19 nt, and minimum hit 100 reads. sRNA reads from each sample were counted exactly as they were based on the defined sRNA locus space from SiLoCo results and no apportioning method was applied to the read counts. Namely, if a read S was aligned to N places, read S were counted for N times. The typical sRNA loci, by contrast to the null loci, could be characterized by the significantly higher abundance of reads and the prevalence of reads with the same length (rather than distribution of reads with random variable lengths). Based on both read coverage depth and read length distribution, a Bayesian model is applied to estimate posterior probability for a given locus and used this as an indicator of likelihood for a locus to be a *bona fide* sRNA locus (Supplementary Method S1). Briefly, sRNA loci were selected for further investigation based on the following four criteria: (1) the difference of the maximum posterior probability between the fungus containing samples (infected wheat and *in vitro* culture) and the fungus free samples (mock-inoculated healthy wheat) is >0.8; (2) posterior probability for at least one of the infected wheat samples is >0.8; (3) differential expression FDR is <0.05 in at least one comparison between the infected wheat samples and the corresponding mock samples; and (4) dominant length of sRNAseq reads that map to the locus must be <25 nt.

For the sRNA differential expression analysis, comparisons between *Z. tritici-*inoculated and the corresponding mock-inoculated samples were performed for each sRNA locus. The quantitative measurement of sRNA expression was based on reads counts. No apportioning was done before the differential expression analysis as any biased estimation in apportioning could bring true negative and false positive results as well. Moreover, apportioning sRNA read counts (21–30 nt) based on the adjacent exon/region expression in gene expression analysis was not possible as for sRNA there is not adjacent region to estimate to what extend to apportion, whereas we reckoned that a simplified apportioning based on the numbers of identical sRNA loci would not likely impact a differential expression analysis, only reducing the total counts by a constant number for both control and treated samples. The raw data counts on sRNA locus space were normalized by the trimmed mean of *M*-values (TMM) method (Robinson and Oshlack, 2010). Differential expression analysis was performed using the edgeR package (Robinson et al., 2010) and a generalized linear model using negative binomial distribution was applied.

For sRNA target prediction, the input sRNA sequences were 21–24 nt reads from each sRNA locus, and the target DNA regions were the wheat cv. Chinese Spring chromosome-specific cDNA sequence database produced by the International Wheat Genome Sequencing Consortium (IWGSC) in late 2015, at the time when this bioinformatics part of the study was done^6^. An initial sRNA target prediction was done using the microRNA target prediction tool (UEA sRNA toolkit). To meet more stringent target criteria, an R script wrapped in Python was written to filter out the initial results^7^. Briefly, sRNA target prediction criteria followed the (Weiberg et al., 2013) study: (1) no gap or bulge is allowed for sRNA/target duplex; (2) no mismatches in positions 10–11; (3) no adjacent mismatches in positions 2–12; (4) cutoff score ≤4.5, where the mismatch and G-U match penalty is 2 and 1, respectively in position 2–12 and mismatch penalty 1 and G-U match penalty 0.5 in other positions; and (5) minimum free energy (MFE) of sRNA-target duplex >0.74.

The RNA Sequencing (RNAseq) data from the *Z. tritici* IPO323 infection time-course on a fully susceptible wheat cv. Riband and the corresponding mock-inoculated wheat leaf samples produced in our previous study [(Rudd et al., 2015); the National Center for Biotechnology Information Sequence Read Archive (SRA), project PRJEB8798] was re-analyzed by mapping the clean sequencing reads with Bowtie 2 (Langmead and Salzberg, 2012) to the chromosome-specific protein coding sequences (CDS) database of wheat cv. Chinese Spring^8^. Transcript abundances were quantified from the SAMtools (Li et al., 2009) sorted BAM files using eXpress (Roberts and Pachter, 2013) and only the sequences with >1 raw CPM in at least one time-treatment were kept for downstream analyses. Differential expression analysis was performed using the edgeR package (Robinson et al., 2010) and a generalized linear model using negative binomial distribution was applied. The Benjamin–Hochberg false discovery rate (FDR) correction was used to adjust *p*-values based on the exact Fisher test (Robinson et al., 2010). The genes were considered differentially expressed if they had a log fold change ≥1 or ≤−1 and FDR ≤0.05. Wheat transcript sequences were annotated using the Mercator pipeline (Lohse et al., 2014).

This study used the protein coding genes annotation of the *Z. tritici* IPO323 genome generated by the United States Department of Energy (DOE) Joint Genome Institute (JGI). It is available from Ensembl Fungi^9^. Repeats and transposable elements annotation of the *Z. tritici* IPO323 genome was kindly provided by Eva Stukenbrock (Grandaubert et al., 2015).

### RNA Extractions for RT-PCR Analyses

All sample material was snap frozen in liquid nitrogen and homogenized using a mortar and pestle. RNA was recovered using Trizol reagent (Invitrogen) following the manufacturer’s instructions, with a 3 h ethanol precipitation at −20°C to ensure maximal recovery of sRNA. Total RNA was treated with RQ1 DNase (Promega) following the manufacturer’s instructions. DNA-free total RNA was recovered by ethanol precipitation for 3 h at −20°C followed by quantification using a NanoDrop spectrophotometer.

### Stem-Loop qRT-PCR

Reactions were assembled following the stem-loop quantitative reverse transcription protocol and the miRNA SYBR Green 1 assay protocol, both previously described (Varkonyi-Gasic et al., 2007) with the exception that SYBR Green JumpStart Taq ReadyMix (Sigma) was used. Reactions were run on a CFX384 real-time PCR system (Biorad) using the following thermocycle: 95°C for 2 min, then 40 cycles of 95°C (15 s), 60°C (15 s), 72°C (15 s). This followed by melt curve analysis of products: 95°C (10 s), then 65 – 95°C at 0.5°C increments with 5 s at each. Relative expression values for each fungal sRNA were calculated using the 2-ΔCt method with normalization to *Z. tritici β-tubulin*. All stem-loop qRT-PCR experiments had 3 biological replicates and each experiment was repeated at least twice with similar results.

### qRT-PCR

The 1–2 μg DNA-free total RNA was used as template for cDNA synthesis using Superscript III (Invitrogen). cDNA was diluted 1:10 with distilled water prior to assembling reactions. SYBR Green JumpStart Taq ReadyMix (Sigma) reactions were assembled following the manufacturer’s instructions. Reactions were run on a CFX384 real-time PCR system (Biorad) using the following thermocycle: 95°C for 3 min, then 40 cycles of 95°C (30 s), 60°C (30 s), 72°C (30 s). This followed by melt curve analysis of products: 95°C (10 s), then 65 – 95°C at 0.5°C increments with 5 s at each. All qRT-PCR experiments had 3 biological replicates and each experiment was repeated at least twice with similar results.

### Fungal Mutagenesis

Generation of gene deletion strains using *Agrobacterium tumefaciens* mediated transformation of *Z. tritici* IPO323 Δ*ku70* strain (Bowler et al., 2010) has been described previously (Motteram et al., 2009). Several independent transformants with each gene deleted were validated by PCR on genomic DNA and 3 randomly chosen verified mutants for each target gene were selected for fungal bioassays. Primer sequences used for the development of single gene deletion constructs and validation of transformants are shown in Supplementary Table S1.

### Fungal Bioassays

Inoculation of *Z. tritici* strains to wheat seedlings and assessment of disease was done following a previously described procedure (Motteram et al., 2009; Rudd et al., 2015). Three randomly chosen verified mutant strains for each target gene were subjected to the bioassays. Each individual mutant strain and a “wild type” control *Z. tritici* IPO323 Δ*ku70* strain was tested by inoculation onto at least 4–5 individual wheat seedlings. The experiment was repeated at least thrice with similar results.

### 5′-RACE

Assays were performed using components of the GeneRacer kit (Invitrogen) using the method of Llave et al. (2002). DNA-free RNA from both mock (negative control) and fungus-infected plant tissue were used as templates. The interaction between Ta-miR156 and the *TaSPL3* transcript was used as a positive control (Liu et al., 2017).

### *Barley Stripe Mosaic Virus* (BSMV)-Mediated HIGS

Approximately ∼300-bp fragments of coding sequences (CDS) of *Z. tritici* genes *ZtTUBa* (*Mycgr3G76039*), *ZtTUBb* (*Mycgr3G102950*), *ZtCYP51* (*Mycgr3G110231*), and *ZtALG2* (*Mycgr3G75289*) or the Green Fluorescent Protein (GFP)-encoding gene were selected for development of BSMV-HIGS constructs following interrogation of *Z. tritici* IPO323 and wheat transcript databases using si-Fi v. 3.1.0 (siRNA Finder^10^) software. These fragments were then amplified by PCR from total cDNA from an *in vitro* cultured *Z. tritici* IPO323, the full-length cDNA clone WT-CYP51 (kindly provided by Hans Cools, Rothamsted Research) or the BSMVγ::GFP plasmid (Haupt et al., 2001) using Invitrogen Taq DNA Polymerase (Thermo Fisher Scientific) and primers carrying 5′ ligation independent cloning (LIC) adaptors specified in Supplementary Table S1. Each PCR product was cloned into the binary BSMV vector pCa-γbLIC using LIC (Yuan et al., 2011) in antisense orientation. Sequence verified HIGS constructs pCa-γbLIC:asZtTUBa, pCa-γbLIC:asZtTUBb, pCa-γbLIC:asZtCYP51, pCa-γbLIC:asZtALG2, and pCa-γbLIC: asGFP were then transformed into the *A. tumefaciens* strain GV3101 by electroporation and used in conjunction with *A. tumefaciens* strains transformed with pCaBS-α (BSMV RNAα) and pCaBS-β (BSMV RNAβ) for Agrobacterium mediated inoculation of *Nicotiana benthamiana* plants following a procedure described in Lee et al. (2015b). Sap from the virus infected *N. benthamiana* plants was used for rub-inoculation of wheat cv. Riband plants. Wheat inoculation with BSMV:asTaChlH served as a positive control for RNA silencing (Lee et al., 2015b). Each BSMV-HIGS and control construct was inoculated onto at least 12 wheat seedlings. In BSMV-mediated RNAi, individual plants undergo separate silencing program and therefore we consider each plant as a separate sample. At 14 days post-virus inoculation, for each BSMV-HIGS and control construct at least 8 individual plants (i.e., replicated samples) showing typical virus-induced mosaic symptoms were challenge inoculated with *Z. tritici* IPO323 and progression of fungal infection was visually monitored during the next 22–25 days, following the final visual assessment of the disease as well as by quantifying asexual fungal sporulation using the previously described procedures (Lee et al., 2015b). Additional experimentation carried out using the same BSMV-HIGS and control constructs but another *Z. tritici* strain, known as “B3” (Rohel et al., 2001), generated similar results.

### Assessment of dsRNA Uptake

Capacity to uptake exogenously applied control long dsRNA and sRNA by *Z. tritici* isolate IPO323 and *B. cinerea* isolate B05.10 was assessed as follows. Fungal material was harvested by scratching from culture plates and adjusting in liquid minimal synthetic nutrient medium (SNA) to approximately 10^5^ spores⋅mL^−1^. Fungal spore preparations were co-incubated with 100 nM BLOCK-iT Alexa Fluor Red siRNA (ThermoFisher catalog No. 14750100) or with 1 μg Cy3-labeled *in vitro*-transcribed 250-bp long dsRNA against GFP in microtiter plates at 21°C, shielded from light. Uptake of fluorescently labeled RNA molecules by germinated spores extending hyphae was monitored after 12 and 48 h of incubation using a fluorescence microscope, and images were captured with bright field and fluorescent (absorption 555 nm, emission 565 nm) settings. The experiment was repeated 3 times with similar results.

### *In vitro* RNAi

Pairs of PCR primers, each carrying the same extension sequence (5′-tcctaatacgactcactatagggag-3′) at their 5′-end, that corresponds to a binding site for T7 RNA Polymerase, were used for amplification of ∼300-bp fragments of CDS of *Z. tritici* genes *ZtTUBa*, *ZtTUBb*, and *ZtCYP51* or the negative control GFP-encoding gene (Supplementary Table S1). The pCa-γbLIC derivatives described above were used as templates for PCRs that were performed using Invitrogen Taq DNA Polymerase (Thermo Fisher Scientific). The resulting PCR products served as templates for the synthesis of double-stranded RNA (dsRNA) using the Ambion^®^MEGAscript^®^RNAi kit (Thermo Fisher Scientific) following the manufacturer’s instructions. Three different amounts of dsRNA (12.5, 125, and 1250 ng) in 2.5 μl elution buffer (Ambion^®^MEGAscript^®^RNAi kit) were mixed with 100 μl water suspensions of *Z. tritici* IPO323 conidiospores at four different concentrations (5 × 10^6^, 5 × 10^5^, 5 × 10^4^, and 5 × 10^3^ mL^−1^) in wells of 96-well culture plates. The plates were incubated at room temperature for 1, 4, and 20.5 h following which the two 5 μl aliquots of germinating spores were taken from each well and spotted as replicas onto Petri dishes containing Yeast Peptone Dextrose Agar (YPDA). The YPDA plates were incubated at 17°C in the dark and the appearance and growth of fungal colonies was monitored macroscopically and by viewing under a binocular microscope over the period of 7 days. Because of the clearly negative results obtained, this experiment was not repeated and the corresponding data presented here as the Supplementary Material.

## Results

### Identification and Validation of Fungal sRNA Transcriptionally Induced *in planta*

The availability of a fully sequenced genome for *Z. tritici* isolate IPO323 allowed identification of the key components of RNA silencing machinery in this fungal species. Only one gene, *Mycgr3G47983*, is annotated as the “DsRNA-specific nuclease” in the *Z. tritici* IPO323 genome (Ensembl Fungi, release 41). BLAST analyses using this gene (named *ZtDCL*), the corresponding protein sequence, or the well-characterized *DCL-1* (*NCU08270*) and *DCL-2* (*NCU06766*) genes of the model fungal species *Neurospora crassa* against the *Z. tritici* IPO323 genome revealed no additional sequences potentially encoding DCLs. Similar analyses revealed the presence of four candidate AGO protein encoding genes: *Mycgr3G38035*, *Mycgr3G10621*, *Mycgr3G90232*, *Mycgr3G25632*. However, only the first two (named *ZtAGO1* and *ZtAGO2*, respectively) appeared to be functional with the other two coding for truncated proteins missing one or more essential N-terminal domains and showing very low level of expression (Supplementary Figure S1 and Supplementary Table S2). Two candidate RdRp encoding genes, *Mycgr3G51407* and *Mycgr3G49833*, were also identified. All these genes encoding components of the RNAi machinery identified in the reference isolate IPO323 are also present in the four Swiss *Z. tritici* isolates, 3D1, 1A5, 1E4, and 3D7, for which the genome data is available from Ensembl Fungi. This provided indirect evidence that a system for sRNA biogenesis may exist in *Z. tritici*.

We therefore carried out deep sequencing of sRNA preparations from *in vitro* cultured fungus, as well as from the infected fully susceptible wheat cv. Bobwhite plants. Over 9.5 million reads ranging in size from 16 to 52 nt that mapped to the fungal genome were obtained from the *in vitro* samples. Moreover, over 0.74, 1.28, 2.65, and 3.45 million reads that mapped to the *Z. tritici* IPO323 genome were obtained from the infected wheat leaf tissues sampled at the four different time points corresponding to the four critical stages of disease development (Supplementary Table S3), namely 4 dpi (“biotrophic”/ asymptomatic stage), 9 dpi (transition from “biotrophic” to “necrotrophic” stage), 13 dpi (“necrotrophic” stage), and 21 dpi (profuse asexual sporulation) (Rudd et al., 2015). This provided direct evidence that *Z. tritici* is capable of generating sRNAs during both *in vitro* and *in planta* growth. The fungal sRNA length distribution was much broader than that typical of vascular plants. Also, in addition to the typical ∼20–24 nt sRNA peaks, two extra peaks centered at ∼30 and ∼50 nt were observed in the samples (Supplementary Figure S2). Further analysis indicated that the majority of these longer sequences originate either from tRNA or rRNA loci, or from wheat chloroplast sequences (data not shown).

**FIGURE 1:**
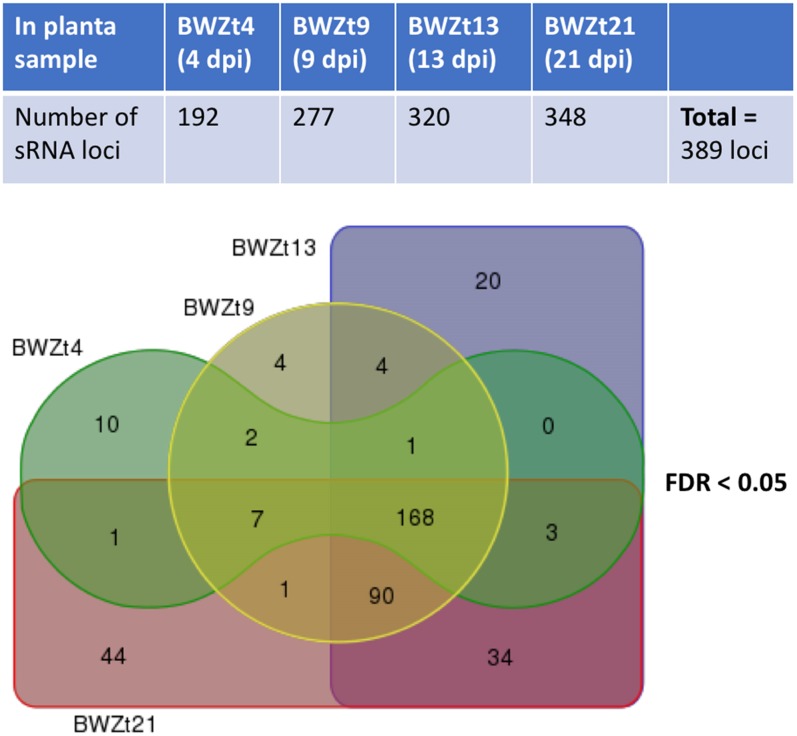
Predicted *Zymoseptoria tritici* sRNA loci active during different timepoints of wheat infection. Numbers of sRNA loci upregulated at 4, 9, 13, and 21 days post-inoculation (dpi) of a susceptible wheat cv. Bobwhite with *Z. tritici* isolate IPO323 are shown in the upper panel. A Venn diagram showing numbers of infection stage-specific sRNA loci as well as those shared between the consecutive infection timepoints is presented in the lower panel. False discovery rate (FDR) rate for differential sRNA loci expression in at least one comparison between the infected wheat samples and the corresponding mock samples was set to <0.05.

All adapter trimmed sRNA reads that mapped to the *Z. tritici* IPO323 genome without mismatches and had no perfect matches in the wheat genome were used for sRNA loci prediction using SiLoCo. This resulted in the identification of 1619 candidate sRNA loci. sRNA reads were counted based on the defined sRNA loci for each sample. Based on the read count abundance and read length distribution, posterior probability per sRNA locus per sample was estimated. sRNA loci differential expression between infected and mock samples were also estimated. sRNA loci were selected for further investigation following a pipeline described in the section “Materials and Methods,” and Supplementary Method S1. All sRNA loci annotated as rRNAs and tRNAs were excluded from further analysis apart from a small number of those that mapped to the non-coding strand of these genes. This analysis funneled down the candidate sRNA loci list from 1619 to 389 potentially genuine sRNA loci that differed in length and total expression level (Supplementary Table S4). Over three quarters of these sRNA loci originated from intergenic regions and overlapped with transposable elements (TEs) and unclassified repetitive elements, whereas only 66 out of 389 loci overlapped with genes (Supplementary Figure S3 and Supplementary Table S4). There were also 34 loci, which may or may not be genuine, that overlapped with non-coding RNA genes (Supplementary Figure S3 and Supplementary Table S4). All these sRNA loci were active at one or sometimes at several, often adjacent timepoints during wheat infection (Figure 1). Notably, all chromosomes of *Z. tritici* including the 13 core and 8 shorter accessory (dispensable) chromosomes (Goodwin et al., 2011) were predicted to host sRNA loci (Supplementary Figure S4 and Supplementary Tables S4, S5). As the predicted *Z. tritici* sRNA loci and chromosomes differ in length substantially we therefore calculated for each chromosome the percentage occupied by sRNA loci. This revealed that all accessory chromosomes, apart from chromosome 19, were substantially more densely covered by sRNA loci (Supplementary Table S5). Nevertheless, sRNA loci identified on accessory chromosomes appeared to show activities comparable to those of sRNA loci residing on core chromosomes. That is the interrogation of the sRNAseq dataset revealed that each 1 kb of sRNA loci located on accessory and core chromosomes generates on average from 991 (chromosome 21) to 3127 (chromosome 19) sRNA reads, and from 1323 (chromosome 10) to 4544 (chromosome 11) sRNA reads, respectively (Supplementary Table S5).

**FIGURE 2:**
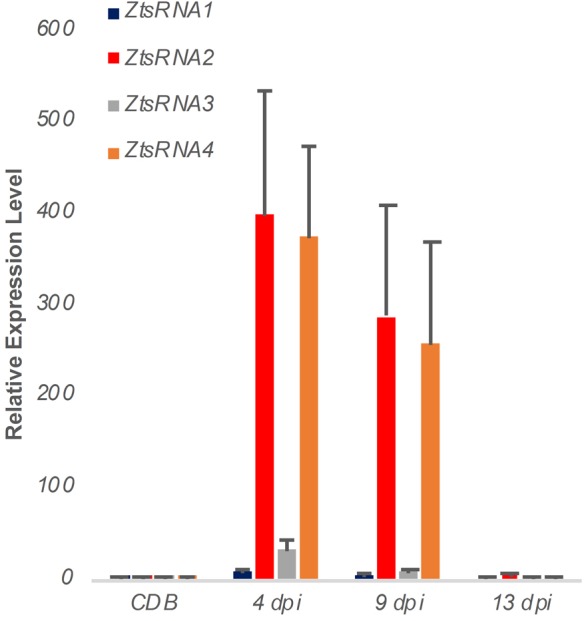
Expression profiling of *Zymoseptoria tritici* mature sRNAs by stem-loop qRT-PCR. Relative expression of four *Z. tritici* sRNAs (ZtsRNA1 – ZtsRNA4) from the fungus grown *in vitro* (CDB) or *in planta* (leaf tissue at 4, 9, and 13 days post-inoculation, dpi). There were 3 biological replicates in this experiment. Bars indicate SE.

To verify the sRNAseq data, we performed a new *Z. tritici* IPO323 infection time course on the susceptible wheat cv. Bobwhite, sampling the leaf tissue at 4, 9, and 13 dpi. RNA from this material was used to assess an *in planta* expression of four fungal sRNAs, selected from the sRNAseq dataset, using a sequence guided stem-loop qRT-PCR assay (Figure 2). The same technique was also used to evaluate expression of these sRNAs in liquid (CDB) fungal culture (Figure 2). Expression of these sRNAs in the infected plant material was found to be considerably higher than in the fungus cultured *in vitro* (Figure 2). Expression was highest at the 4 and 9 dpi timepoints, which correspond to the symptomless and transition phases of colonization by this fungus. By 13 dpi, when significant regions of necrotic tissue were present in leaves infected under glasshouse conditions, the expression of all four fungal sRNAs had returned to levels similar to those identified *in vitro* (Figure 2). These experiments demonstrated that all fungal sRNAs investigated were significantly induced during wheat infection, thereby validating the data generated by sRNAseq.

### sRNA Target Prediction: Identification and Validation of Candidate Wheat Transcripts Repressed During Fungal Infection

For a given sRNA locus, 21–24 nt sequences were retrieved from each infected sample alignment bam file for target prediction on transcripts of the wheat cv. Chinese Spring (the only wheat genotype for which the draft genome sequence was available at the time of the study). Here we used sRNA target prediction criteria similar to those utilized in the previously published study (Weiberg et al., 2013). This identified a total of 262 sRNAs, with 140 of which having unique sequences and 122 others falling into 43 sequence related groups (Supplementary Table S6), that were computationally predicted to target 737 wheat transcripts (Supplementary Table S7). Majority of these sRNAs were 21 nt long and displayed a bias toward uridine in their 5′ termini (Supplementary Figure S5), features characteristic of functional sRNAs capable of directing cleavage or translational silencing of the corresponding target mRNAs.

**FIGURE 3:**
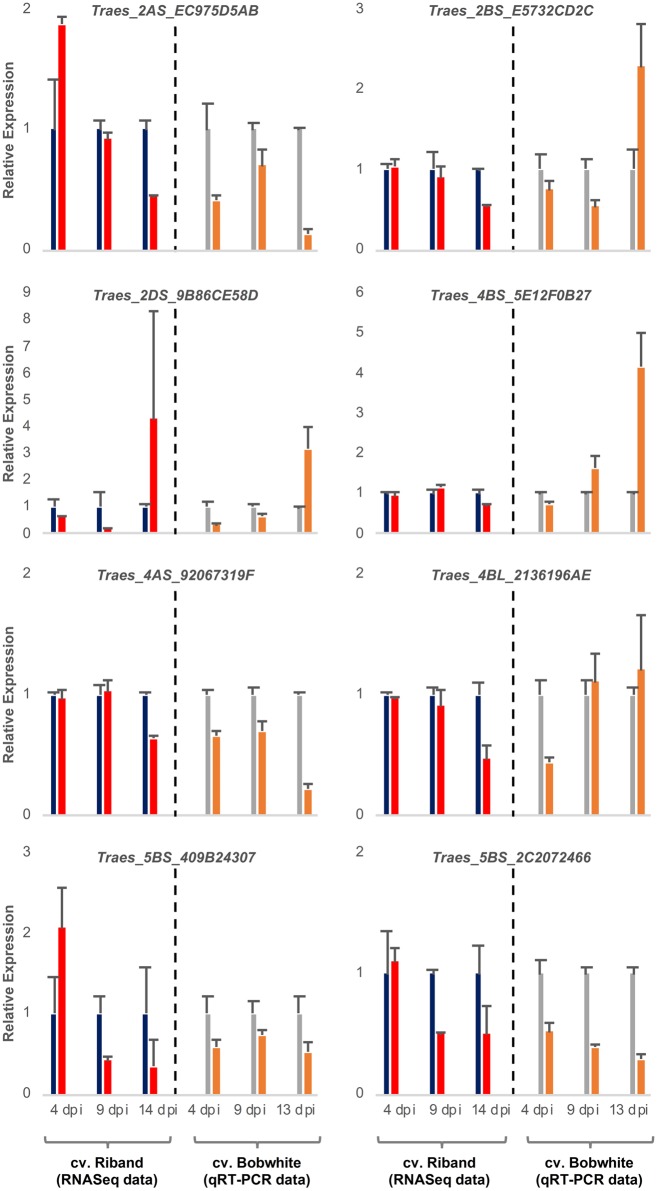
Expression profiling of eight candidate wheat target mRNAs during *Zymoseptoria tritici* IPO323 infection of wheat cv. Bobwhite leaf tissue. Left panels show RNAseq data [re-analysis of Rudd et al. (2015)] for candidate genes converted to relative expression for presentation for mock (blue) and *Z. tritici* IPO323 (red) treatments. Right panels show qRT-PCR analysis of duplicate sample set, mock (gray) and *Z. tritici* IPO323 (orange). Expression levels were rescaled such that the mock infection treatment at each timepoint represents a value of 1. There were 3 biological replicates in this experiment. Bars indicate SE.

It was expected that the wheat transcripts successfully targeted by fungal sRNAs would display significant downregulation during either specific or all stages of the *Z. tritici* – wheat infection. To predict wheat genes whose expression was significantly reduced during fungal infection we re-analyzed our previously published RNAseq data set from the *Z. tritici* IPO323 infection time course on the fully susceptible wheat cv. Riband and the corresponding mock-inoculated plants (Rudd et al., 2015) by re-mapping the sequence reads to the wheat cv. Chinese Spring chromosome-specific CDS “Triticum_aestivum. IWGSC2.26.cds.all.fa” followed by differential gene expression analysis. This data helped us to prioritize 10 wheat mRNAs, predicted to be targeted by the four selected *Z. tritici* sRNAs for cleavage, for further investigation based on their expression profile and potential role in pathogen defense (Table 1 and Supplementary Table S7). Most of these wheat transcripts exhibited reduced expression in the infected wheat cv. Riband tissue compared to the corresponding mock-inoculated healthy control plants in at least one of the three assessed timepoints, with the expression levels often being lower at 9 and 14 dpi (transition and necrotrophic phases) than at 4 dpi (symptomless phase) (Figure 3). However, there were also some instances where expression of predicted target mRNAs was higher in the infected tissue in comparison to the mock-treated controls, specifically for Traes_2AS_EC975D5AB and Traes_5BS_409B24307 at 4 dpi and for Traes_2DS_9B86CE58D at 14 dpi (Figure 3). A qRT-PCR was then used to analyze expression of these predicted target mRNAs during *Z. tritici* IPO323 infection of wheat cv. Bobwhite (Figure 3). All target wheat mRNAs again showed downregulation in the infected leaf tissue compared to the mock-treated controls in at least one infection timepoint. Therefore, the qRT-PCR data for wheat cv. Bobwhite agreed well with the expression data obtained using RNAseq for wheat cv. Riband, indicating similar regulation of expression of these mRNAs in the two different fully susceptible wheat cultivars. Expression profiles of the two mRNAs, Traes_2BS_E5732CD2C and Traes_4BS_5E12F0B27, showed some differences between the two cultivars (Figure 3). However, alternatively, this may be due to qRT-PCR assays being more effective than RNAseq in distinguishing between highly similar homoeologous transcripts.

**Table 1:** *Zymoseptoria tritici* IPO323 mature sRNAs selected for detailed study and their respective wheat mRNA targets.

sRNA code	sRNA sequence	Wheat transcript target code	Wheat transcript ID	Wheat transcript annotation	Alignment
ZtsRNA1	TTGGGGAATCCGT AGTGTTGT	mRNA1-1	Traes_2AS_EC975D5AB	Alpha-N-arabino-furanosidase A	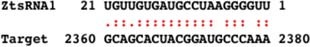
ZtsRNA1	TTGGGGAATCCGTA GTGTTGT	mRNA1-2	Traes_2BS_E5732CD2C	Alpha-N-arabino-furanosidase A	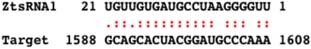
ZtsRNA1	TTGGGGAATCCGTA GTGTTGT	mRNA1-3	Traes_2DS_9B86CE58D	alpha-N-arabino-furanosidase A	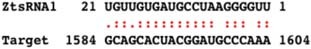
ZtsRNA2	TGCACTGGTTGCTC GAACGCT	mRNA2	Traes_4BS_5E12F0B27	Probable S/T protein kinase IREH1	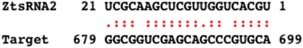
ZtsRNA3	TAACCATCTTTCGGG TCTGACT	mRNA3-1	Traes_4AS_92067319F	NAC domain superfamily	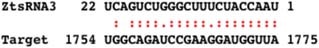
ZtsRNA3	TAACCATCTTTCGGG TCTGACT	mRNA3-2	Traes_4BL_2136196AE	NAC domain superfamily	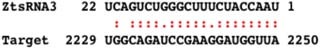
ZtsRNA3	TAACCATCTTTCGGG TCTGACT	mRNA3-3	Traes_4DL_28E7C238E	NAC domain superfamily	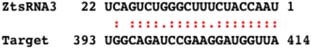
ZtsRNA4	TGGAGATCGCGAAG GAGGTTC	mRNA4-1	Traes_5BS_409B24307	Glutaredoxin, thioredoxin-like	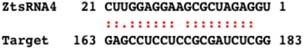
ZtsRNA4	TGGAGATCGCGAAG GAGGTTC	mRNA4-2	Traes_5BS_2C2072466	Glutaredoxin, thioredoxin-like	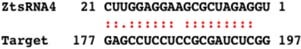
ZtsRNA4	TGGAGATCGCGAAG GAGGTTC	mRNA4-3	Traes_5DS_286CDF1FE1	Glutaredoxin, thioredoxin-like	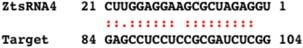

*Potential homoeologous gene transcripts are shown in the same color.*

### RNAi-Deficient Fungal Mutant Strains Are Fully Pathogenic on Wheat

**FIGURE 4:**
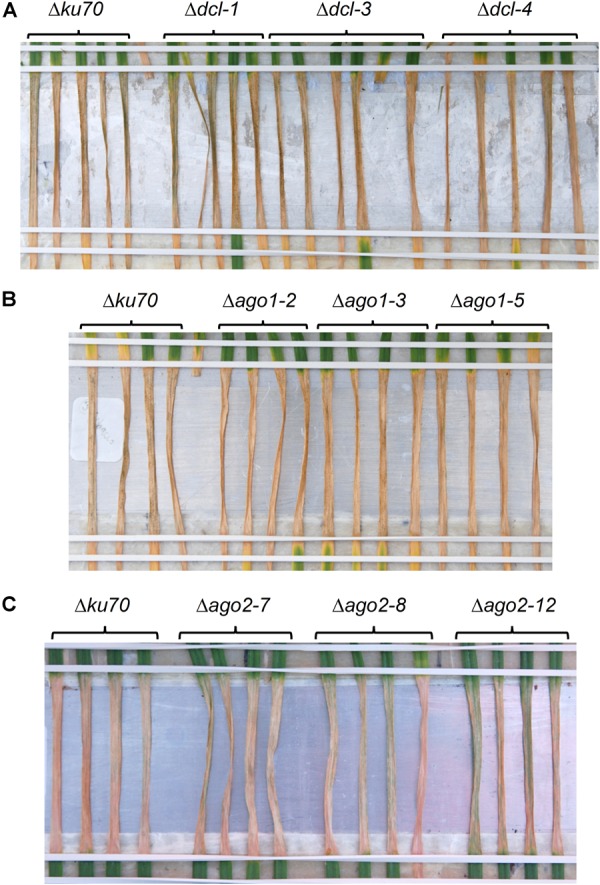
*Zymoseptoria tritici* infection bioassays. *Z. tritici* IPO323 strain Δ*ku70* and its derivatives deficient in RNA silencing pathway (Δ*dcl*, Δ*ago1*, or Δ*ago2*) were inoculated onto the wheat cv. Bobwhite. Three randomly chosen mutant strains for each target gene were tested by inoculation onto at least 4–5 individual wheat seedlings, and this data is shown in panels **(A–C)**. Fungal inoculations were done using suspension of conidiospores at 1 × 10^6^ mL^−1^ and the inoculated leaves were phenotyped and photographed at 16 days post-inoculation.

To further examine the potential role of the fungal RNAi pathway in a compatible interaction between wheat and *Z. tritici* we produced several *Z. tritici* mutants deficient in the key RNAi components. Gene deletion strains for the single predicted *ZtDCL* gene and both of the genes, *ZtAGO1* and *ZtAGO2*, predicted to encode Argonaute proteins were generated and validated by PCR. All mutant strains were produced in the *Z. tritici* IPO323 Δ*ku70* background (Bowler et al., 2010) which maximizes homologous recombination. Three independent mutants for each target gene were selected for further study. In replicated infection bioassays using the susceptible wheat cv. Bobwhite, all generated mutant strains, Δ*dcl*, Δ*ago1*, and Δ*ago2*, induced similar levels of disease to the parental IPO323 Δ*ku70* strain. That is, no appreciable change in the percentage of leaf area affected by necrosis (Figure 4) and or pycnidia density (Supplementary Figure S6) between the parental strain and any of the three mutant strains was apparent. Moreover, rates of disease lesion development on the infected leaves were similar to the IPO323 Δ*ku70* control in all experiments, with inoculation zones becoming fully necrotic by 16 dpi (Figure 4). These experiments therefore demonstrated that *ZtDCL*, *ZtAGO1*, and *ZtAGO2* genes are dispensable for virulence.

### Evidence for DCL-Independent Biogenesis of sRNA in *Z. tritici*

**FIGURE 5:**
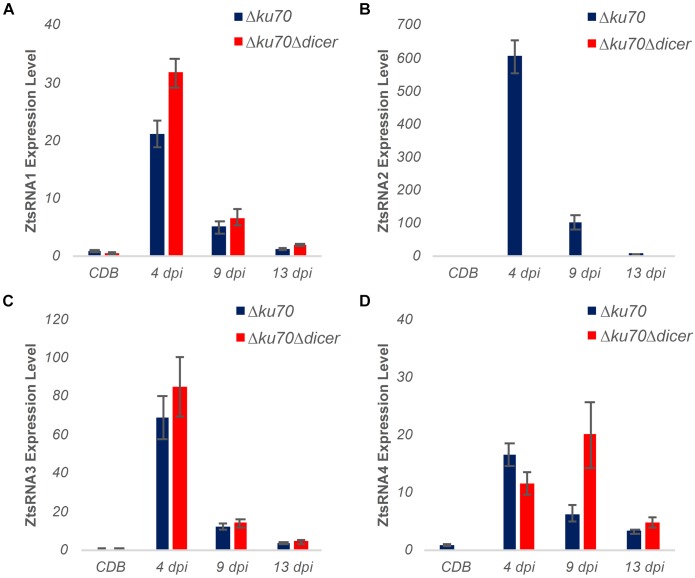
Expression profiling of fungal sRNAs in an RNAi-deficient *Zymoseptoria tritici* mutant Δ*dicer* (IPO323 Δ*ku70*Δ*dcl*). **(A–D)** Stem-loop qRT-PCR analysis of four ZtsRNAs from *Z. tritici* IPO323 Δ*ku70* (blue) and *Z. tritici* IPO323 Δ*ku70*Δ*dcl* (red). Fungal material from an *in vitro* culture (Czapek-Dox Broth, CDB) and *in planta* (leaf tissue at 4, 9, and 13 days post-inoculation, dpi) was assessed. There were 3 biological replicates in this experiment. Bars represent the mean and SE. The *Z. tritici* IPO323 Δ*ku70* (CDB) samples were set to 1 in all panels.

As described above, the *Z. tritici* mutant Δ*dcl* displayed no reduction in virulence in the wheat infection bioassay. To further investigate the role of fungal sRNAs as potential virulence factors during infection by this foliar pathogen, we carried out new wheat infection time courses with both *Z. tritici* IPO323 Δ*ku70* (control) and the Δ*dcl* mutant followed by stem-loop qRT-PCR analysis of fungal sRNA expression (Figure 5). Unexpectedly, expression of three out of four fungal sRNAs investigated, namely ZtsRNA1, ZtsRNA3, and ZtsRNA4, was maintained in the Δ*dcl* mutant at levels comparable to those in IPO323 Δ*ku70* control (Figure 5). Moreover, as with the control strain, sRNA expression of these 3 sRNAs in the Δ*dcl* mutant was considerably higher when the fungus was recovered from the infected wheat leaf tissue in comparison to the fungus cultured *in vitro* (Figure 5). Highest sRNA expression levels were detected at either 4 dpi (ZtsRNA1, ZtsRNA3) or 9 dpi (ZtsRNA4). In contrast, expression of ZtsRNA2 was abolished in the Δ*dcl* mutant (Figure 5). No expression of this sRNA was detected in the fungus grown either *in vitro* or *in planta*. This data suggested that *ZtDCL* is required for the biogenesis of ZtsRNA2, whereas the other three sRNAs may be generated independently of *ZtDCL*.

To further examine the role of ZtsRNA2 in fungal virulence, a qRT-PCR analysis was performed on the same sample set to assess expression levels of the predicted wheat target mRNA Traes_4BS_5E12F0B27. Surprisingly, expression of this mRNA was similar in the wheat leaf tissue infected with either *Z. tritici* IPO323 Δ*ku70* or Δ*dcl* at all three timepoints (Supplementary Figure S7). Given the absence of ZtsRNA2 in the *Z. tritici* Δ*dcl* mutant, this indicated that Traes_4BS_5E12F0B27 is likely not a genuine target of this fungal sRNA. To verify this, and to assess the interactions between the three other *Z. tritici* sRNAs and their putative targets, 5′-RACE assays were performed to detect mRNA cleavage fragments resulting from targeting. These assays were unable to detect any fragments associated with the predicted sRNA-guided target cleavage (Supplementary Figure S8). Therefore, it appears that none of the wheat mRNAs investigated represent genuine targets of the four fungal sRNAs, ZtsRNA1 – ZtsRNA4.

### Transient Host-Induced Gene Silencing (HIGS) Is Ineffective in Protecting Wheat Plants Against Septoria Tritici Blotch Disease

The data presented above suggested that although *Z. tritici* produces numerous sRNAs both *in vitro* and during infection of wheat, at least those that are *ZtDCL-*dependent do not seem to contribute to fungal virulence. Next, we asked whether the plant-derived sRNAs could translocate and induce RNAi in *Z. tritici* during infection. To address this, we used a plant RNA virus-based vector as a potent RNA silencing inducer in a procedure known as transient HIGS (Lee et al., 2012). In this procedure, a recombinant *Barley-stripe mosaic virus* (BSMV) carrying a fragment of target fungal pathogenicity or essential for life gene is inoculated onto wheat leaves, which triggers a massive production of sRNAs from the replicating virus genome (i.e., long double-stranded RNA, dsRNA) by the plant’s own RNAi machinery (Lee et al., 2012). Uptake of these long dsRNAs or fungal-gene specific sRNAs by *Z. tritici* during attempted infection of BSMV-HIGS-treated plants would be expected to result in substantially reduced disease levels.

**FIGURE 6:**
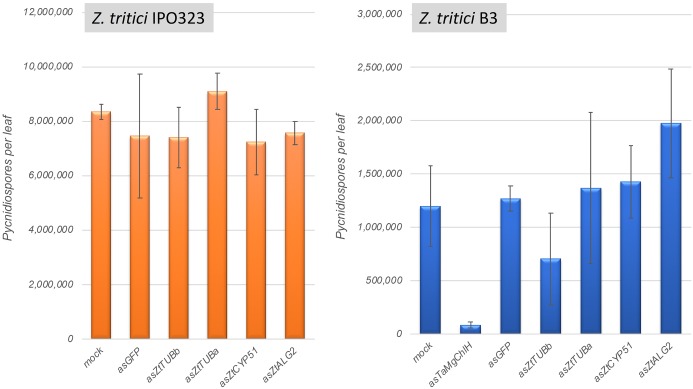
Assessment of *Zymoseptoria tritici* strains IPO323 and B3 pycnidiospore production in wheat cv. Riband plants pre-treated with various BSMV-HIGS and BSMV-VIGS constructs.

Four *Z. tritici* genes were selected for silencing in this experiment: *ZtCYP51* (*Mycgr3G110231*; encodes cytochrome P450 lanosterol C-14α-demethylase), *ZtTUBa* (*Mycgr3G76039*; encodes α-tubulin), *ZtTUBb* (*Mycgr3G102950*; encodes β-tubulin), and *ZtALG2* (*Mycgr3G75289*; encodes α-1,2-mannosyltransferase). The first three of these genes are known fungicide targets (Siegel, 1981; Hollomon et al., 1998), whereas *ZtALG2* is essential for hyphal growth and pathogenicity (Motteram et al., 2011). The similarly sized (∼300-bp) fragments of protein coding sequences were PCR amplified and cloned individually into the BSMV vector pCa-γbLIC in anti-sense orientation. As predicted *in silico* using si-Fi v. 3.1.0 software, all selected target CDS fragments were likely to generate comparable numbers of silencing-efficient small interfering RNAs (siRNAs). Moreover, the selected CDS fragments were not likely to generate siRNAs capable of inducing silencing of off-target genes in *Z. tritici* (except for *ZtTUBa* construct; see Supplementary Table S8) or in its host, wheat. BSMV vectors carrying ∼300-bp fragments of a non-fungal and a non-plant origin gene, such as the jellyfish *Green Fluorescent Protein* (*GFP*) gene, and the wheat *Magnesium Chelatase subunit H* (*TaMgChlH*) gene involved in chlorophyll biosynthesis were used as the negative and positive controls for gene silencing, respectively (Lee et al., 2015b). As expected, upper uninoculated leaves (L3 and L4) of all plants systemically infected with BSMV:asTaMgChlH appeared orangey-yellow rather than green (Supplementary Figure S9, and data not shown), reporting 100% silencing frequency of *TaMgChlH* that resulted in chlorophyll deficiency (Lee et al., 2015b). The rate of disease lesion development on wheat leaves infected with the two different *Z. tritici* strains, IPO323 and B3, was similar on all experimental plants, i.e., those untreated with BSMV and also those pre-treated with the various BSMV-HIGS constructs (apart from BSMV:asTaMgChlH) or a negative control BSMV:asGFP. Visually similar levels of STB disease were observed by 22–25 days post-fungal inoculation, when *Z. tritici* had completed its life cycle (data not shown). The disease was further quantified by counting *Z. tritici* pycnidiospores washed from the diseased leaves to verify the visual assessment. Indeed, very similar counts of pycnidiospores were obtained in washes from the virus-free wheat plants and from those that were pre-treated with BSMV:asGFP or the various BSMV-HIGS constructs (for the exception of BSMV:asTaMgChlH) prior to fungal inoculation (Figure 6). The number of fungal spores washed from each leaf was somewhat higher in the experiments involving *Z. tritici* IPO323. This may be either due to the possible differences in virulence between the different fungal strains, or because in the experiments involving *Z. tritici* IPO323 the leaves were sampled 3 days later post-fungal inoculation and incubated under high relative humidity for 42 h rather than 24 h (before counting pycnidiospores) compared to the experiments involving *Z. tritici* B3 (Figure 6). By contrast and in agreement with our previous study (Lee et al., 2015a) the ability of *Z. tritici* to complete its asexual infection cycle was severely impaired in *TaMgChlH*-silenced wheat plants as only a very small number of pycnidiospores was recovered in washes from these plants (Figure 6). This suggested, albeit indirectly, that the plant sRNA generating silencing pathways were successfully activated in these BSMV-mediated RNAi experiments. However, these experiments failed to generate any evidence suggesting that BSMV-HIGS could work successfully to protect wheat plants against STB disease.

### *Z. tritici* Is Unable to Uptake Externally Applied dsRNAs *in vitro*

**FIGURE 7:**
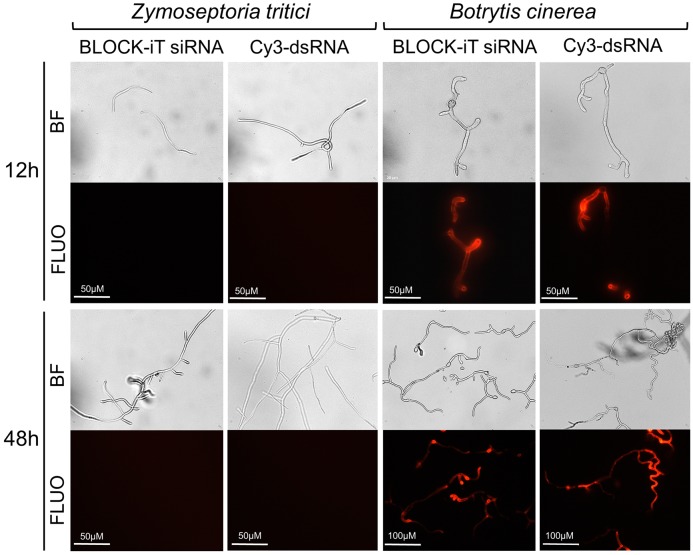
Assessment of dsRNA molecules uptake in *Zymoseptoria tritici* and *Botrytis cinerea*. Uptake of fluorescently labeled short dsRNA (Alexa Fluor Red BLOCK-iT siRNA) or 250-long long Cy3-labeled dsRNA against GFP was monitored at 12 and 48 h of co-incubation using microscopy. Representative images from a single experiment are depicted as captured with bright field (BF) and fluorescent (FLUO) absorption 555 nm, emission 565 nm) settings. The experiment was repeated 3 times with similar results.

To directly assess the uptake of dsRNA by *Z. tritici*, we co-incubated germinating conidiospores in a liquid nutrient-poor medium, which stimulates spores germination and hyphal extension (King et al., 2017), with either Cy3-labeled^®^250 nt long dsRNA derived from the *GFP* gene or Alexa Fluor 555-labeled siRNA in the dark. *B. cinerea* was used as a positive control in these experiments as this fungal species is known to be engaging in bidirectional cross-kingdom RNAi and readily take up external dsRNAs (Wang et al., 2016). Germinated spores were monitored using fluorescent microscopy and, as expected, fluorescent signals were detected in the *B. cinerea* from 12 h of co-incubation with labeled dsRNA species onward (Figure 7). However, no fluorescent signals were detected in germinated *Z. tritici* conidiospores, which clearly extended hyphal strands, even by 48 h post co-incubation with either long or short labeled dsRNA species (Figure 7). Thus, it appears that *Z. tritici* lacks the capacity to uptake exogenous dsRNAs.

In parallel with the dsRNA uptake experiments described above, we also investigated whether external application of dsRNAs targeting essential for life fungal genes could impair or restrict *Z. tritici* growth *in vitro*. We, therefore, treated preparations of germinating *Z. tritici* conidiospores with *in vitro* synthesized ∼300 nt long dsRNAs targeting *ZtCYP51*, *ZtTUBa*, and *ZtTUBb* genes. All these genes as mentioned above are known targets for fungicides, and knock-out mutations in these genes are thought to be lethal as none have been reported to date. GFP-specific dsRNA of a similar size was used as a negative control. The same gene regions of these genes, as in the BSMV-HIGS experiments described above, were selected to serve as templates for production of dsRNAs. Four different concentrations of germinating *Z. tritici* conidiospore preparations (i.e., 10× serial dilutions ranging from 5 × 10^6^ mL^−1^ to 5 ×10^3^ mL^−1^) were incubated in sterile deionized water with the three increasing amounts of dsRNAs (12.5, 125, and 1250 ng per 100 μL volume) for 1 h, 4 h and overnight. After co-incubations with dsRNA, aliquots of fungal suspensions were spotted onto YPD agar plates and incubated at 17°C in the dark for 5–7 days or until fungal colonies could be visualized. No differences were observed in the appearance or growth rates between the colonies originating from untreated *Z. tritici* samples versus those treated with the three different amounts of dsRNA for any of the dsRNA treatments (Supplementary Figure S10, and data not shown).

These experiments combined suggested that *Z. tritici*, in contrast to other fungal species such as *B. cinerea*, *Verticillium dahliae*, *Sclerotinia sclerotiorum*, *Fusarium graminearum*, and *Fusarium asiaticum* (Koch et al., 2016; Wang et al., 2016; McLoughlin et al., 2018), is unable to uptake both external long as well as short dsRNA.

## Discussion

This study represents the most extensive attempt to date to characterize the sRNA transcriptome of the foliar wheat pathogen *Z. tritici* (isolate IPO323), with 389 sRNA loci active at one or more timepoints during infection of a susceptible wheat cv. Bobwhite (Figure 1 and Supplementary Table S4). Furthermore, here we examined the possible role of cross-kingdom sRNA transfer and gene silencing in the interaction between this fungus and its natural host (wheat) and assessed the potential of RNAi as a possible future strategy for control of septoria tritici blotch disease in wheat.

The contribution of the fungal RNAi pathway to virulence was assessed through production of targeted deletions of the *ZtDCL*, *ZtAGO1*, and *ZtAGO2* genes. Fungal mutants for each of these three genes exhibited wild-type levels of virulence on wheat suggesting that these important components of the canonical RNAi pathway in *Z. tritici* are dispensable for successful wheat infection. These mutants also displayed no abnormal yeast-like growth morphology when the fungus was cultured on the rich growth media such as YPD (data not shown). Together, this implied that the *Z. tritici* RNAi pathway is superfluous or plays only a minimal role during either yeast-like or filamentous growth of this species. However, subsequent experiments revealed that expression of some fungal sRNAs is retained in the *Z. tritici* Δ*dcl* mutant, at the level similar to that in the wild-type fungus (Figure 5). Given that this was observed for three of four examined sRNAs of interest (Figure 5), this data provided preliminary evidence that a proportion of the total sRNA pool in *Z. tritici* may be generated in a *ZtDCL*-independent manner. It would be interesting to investigate this further by determining a complete repertoire of sRNAs produced independently of the canonical RNAi pathway for example through sRNA sequencing from *in vitro* and *in planta* samples infected with the *Z. tritici* Δ*dcl* mutant. It would also be of interest to further investigate the components and mechanisms of a Dicer-independent sRNA biogenesis in *Z. tritici* and compare to those that have previously been reported for other fungal species (Chang et al., 2012). Simultaneous inactivation of both, this as yet uncharacterized pathway and a canonical Dicer-dependent sRNA biogenesis pathway, via deletion of key genes involved in both pathways in the same strain will be necessary to definitively conclude the involvement (or lack of) of sRNAs in successful infection by this fungus.

Two hundred and thirty-four *Z. tritici* sRNA loci were predicted to generate sRNAs putatively targeting 737 wheat transcripts with annotations for some suggestive of their potential role in plant immunity (Supplementary Table S7), implying the existence of cross-kingdom RNAi in this pathosystem. It might be expected that the abundance of candidate wheat mRNA targets is reduced during *Z. tritici* infection due to the fungal sRNA-mediated cleavage. However, less than one third of the predicted targets showed downregulation of more than 2-fold even at one time point during *Z. tritici* IPO323 infection of a susceptible wheat cv. Riband, and downregulation of only ∼70 of these transcripts was found to be statistically significant. Moreover, we were unable to detect the predicted ZtsRNA1 – ZtsRNA4 catalyzed cleavage fragments in any of their 10 predicted wheat mRNA targets using 5′-RACE (Supplementary Figure S8). It is possible that this could be because the predicted *Z. tritici* sRNAs act by repressing translation of target wheat transcripts rather than orchestrate transcript cleavage. It may also be possible that the analyzed wheat mRNA targets identified computationally are either not genuine targets, or not sufficiently well-targeted for the effects to be detectable using the methods described here.

The rational for this investigation was based on recent findings describing bidirectional cross-kingdom RNAi for the necrotrophic fungus *B. cinerea* (Wang et al., 2016). The data presented here suggest that for *Z. tritici* fungal RNAi pathways do not play a significant role in the successful colonization of wheat plants. This agrees well with the data from a complementary study by Ma et al. (2018) in which no strong evidence for silencing of wheat genes in the susceptible cv. Drifter by sRNAs of the *Z. tritici* isolate 3D7 was found. It is likely that evasion or manipulation of host immunity by this fungal species is more reliant on previously described mechanism such as the deployment of proteinaceous effectors and /or the production of specific metabolites (Deller et al., 2011; Kettles and Kanyuka, 2016). Whilst we conclude that fungal RNAi plays a minimal role during infection, the genome of *Z. tritici* does encode key components of the canonical RNAi pathway such as DCL, AGOs and RdRps. Therefore, it seemed possible that the plant-derived or exogenously applied long dsRNAs or sRNAs if taken up by *Z. tritici* could induce RNAi in this fungus. However, there were no reductions in the amount of *Z. tritici* infection in the BSMV-HIGS experiments targeting fungal genes essential for life or pathogenicity toward wheat (Figure 6) and also no observable effects on growth of *Z. tritici* when germinating fungal conidiospores were co-incubated with the fungal-derived dsRNA targeting essential genes such as *ZtCYP51*, *ZtTUBa*, and *ZtTUBb* (Supplementary Figure S10). These data could be explained by the observations that *Z. tritici* is incapable or very inefficient in taking up exogenous dsRNAs (Figure 7). We therefore conclude that the prospects for topical chemical intervention via next-generation RNA fungicides or through the generation of the stable transgenic HIGS lines in wheat targeting *Z. tritici* genes that are essential for life appear doubtful. We also hypothesize that the RNAi machinery in *Z. tritici* may either be completely redundant or play a role perhaps during growth under certain stressful conditions, e.g., fungicide treatment or only during specific developmental stages. This would not be unprecedented as some fungal species, including *Ustilago maydis*, which is a serious pathogen of maize, have lost RNAi (Billmyre et al., 2013). Furthermore, knockout of either DCL, AGO, or RdRp encoding genes in another fungal pathogen of wheat, *F. graminearum*, did not compromise pathogenicity toward wheat (Kim et al., 2015; and own unpublished data) and it has been recently demonstrated that the RNAi machinery in *F. graminearum* is mainly involved in the regulation of sexual development (Kim et al., 2015; Zeng et al., 2018). It would be interesting to investigate in future studies whether the same may be true for *Z. tritici*. Also, it seems possible that the RNAi machinery in this fungal species may play a role in defense against transposable elements (Dang et al., 2011) and/or mycoviruses. A mycovirus in several *Z. tritici* isolates has been previously reported and shown to be highly expressed under various stress conditions (Kema et al., 2008), and a full-length sequence of *Z. tritici fusarivirus 1* from *Z. tritici* isolate IPO323 has very recently been reported (Gilbert et al., 2019).

## Author Contributions

GS, KK, KH-K, and MC conceived the project. KK generated material for sRNAseq and re-analyzed the previously published RNAseq data set. sRNAseq analysis and prediction of wheat mRNA targets was done by PH. GK and CB performed experimental validation of fungal sRNA and wheat mRNA expression and developed and characterized *Z. tritici* gene deletion mutants. DB and GS assessed capacity of *Z. tritici* to uptake dsRNAs. JR and DB advised on various aspects of *Z. tritici* biology and RNAi, respectively. BH performed BSMV-HIGS and the *in vitro* RNAi experimentation. GK, BH, KH-K, and KK analyzed the data. GK and KK wrote the manuscript.

## Conflict of Interest Statement

PH was employed by Syngenta Biotechnology Inc., and DB, MC, and GS were employed by Syngenta Crop Protection AG. The remaining authors declare that the research was conducted in the absence of any commercial or financial relationships that could be construed as a potential conflict of interest.

## Footnotes

1 https://github.com/FelixKrueger/TrimGalore

2 https://www.bioinformatics.babraham.ac.uk/projects/fastqc/

3 https://genome.jgi.doe.gov/Mycgr3/Mycgr3.home.html

4 ftp://ftp.ensemblgenomes.org/pub/plants/release-26/fasta/triticum_aestivum/dna/

5 http://srna-workbench.cmp.uea.ac.uk/

6 ftp://ftp.ensemblgenomes.org/pub/plants/release-26/fasta/triticum_aestivum/cdna/

7 https://github.com/PingshaHu/sRNApProbfilter

8 ftp://ftp.ensemblgenomes.org/pub/plants/release-26/fasta/triticum_aestivum/cds/

9 https://fungi.ensembl.org/Zymoseptoria_tritici/Info/Index?db=core

10 http://labtools.ipk-gatersleben.de/
