# Supplementary material for: sRNA Profiling Combined With Gene Function Analysis Reveals a Lack of Evidence for Cross-Kingdom RNAi in the Wheat – Zymoseptoria tritici Pathosystem
Source: Front Plant Sci. 2019 Jul 4;10:892. doi: 10.3389/fpls.2019.00892 (PMC6620828; doi:10.3389/fpls.2019.00892)

Figure S1. Domain architecture of candidate AGO proteins in *Zymoseptoria tritici* isolate IPO323.

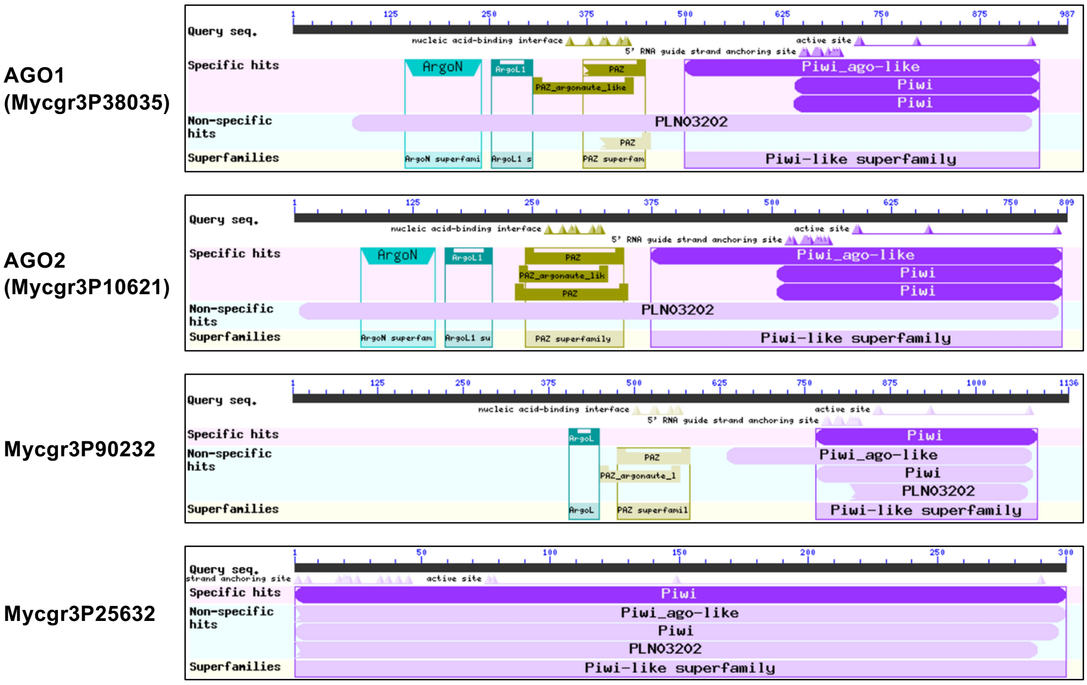

Figure S2. Examples of the distribution of sRNAseq reads mapped to the *Zymoseptoria tritici* IPO323 genome from *in vitro* culture and infected wheat cv. Bobwhite samples at 13 days post inoculation (dpi).

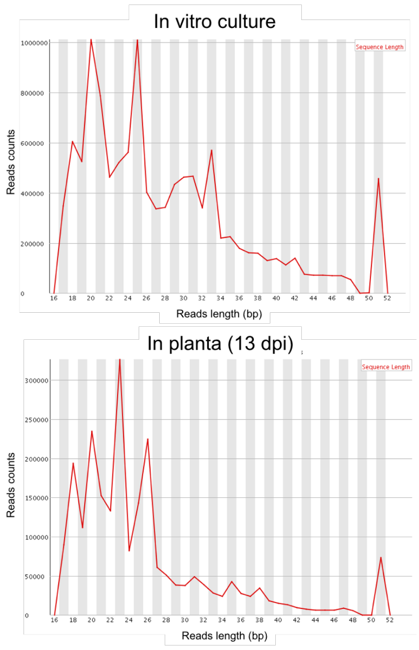

Figure S3. Origin, length and expression levels of 389 sRNA loci identified to be active in *Zymoseptoria tritici* isolate IPO323 during wheat infection.

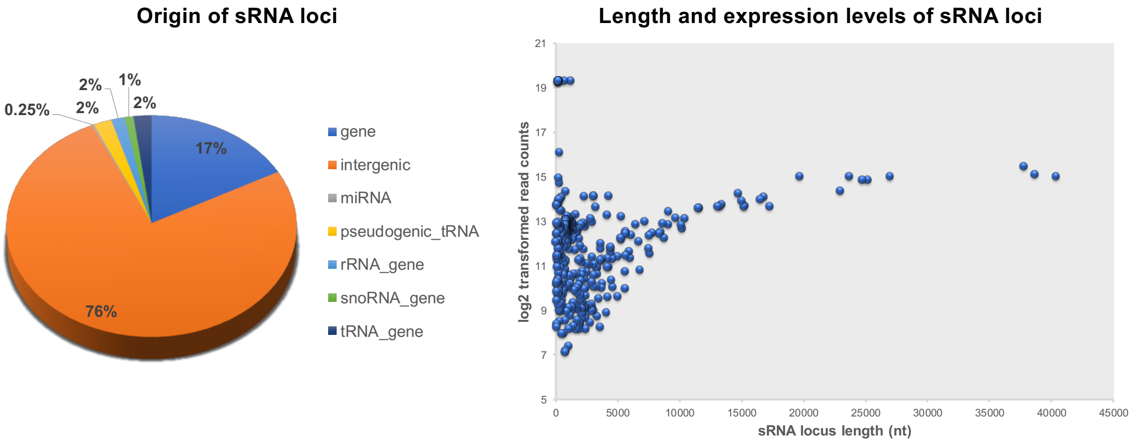

Figure S4. Numbers of sRNA loci residing on individual chromosomes of *Zymoseptoria tritici* isolate IPO323. Core and accessory chromosomes are shown in blue and orange, respectively.

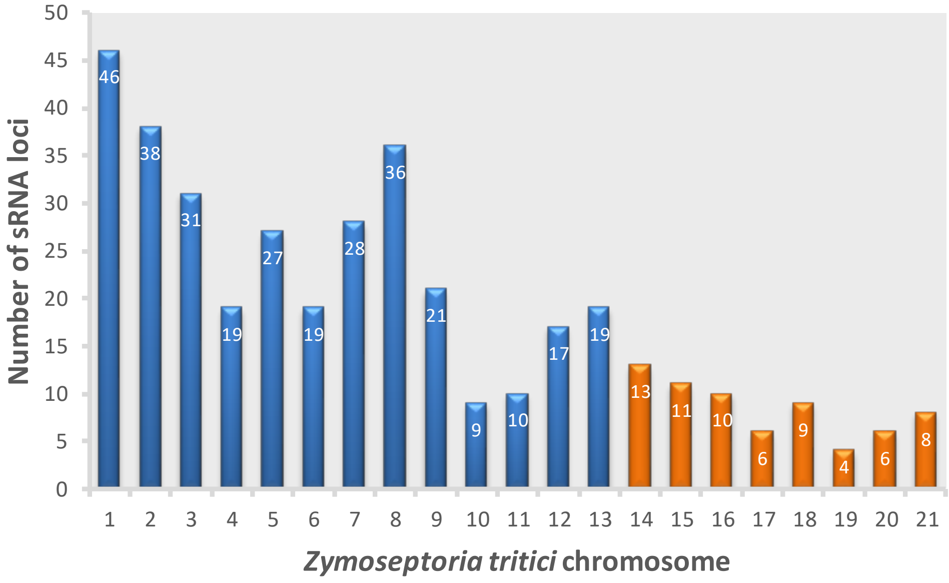

Figure S5. Size distribution and percentage of nucleotides in the 5' position of 262 mature *Zymoseptoria tritici* sRNAs predicted to target wheat transcripts.

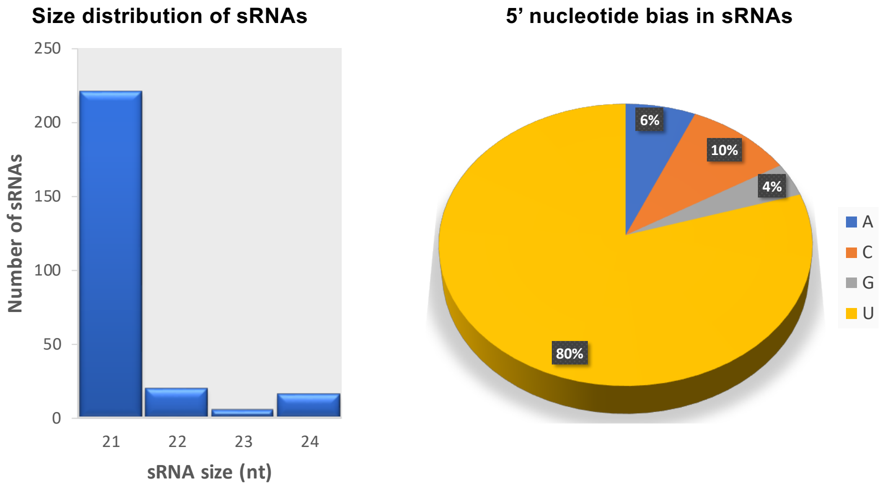

Figure S6. High humidity-induced pycnidiation of *Zymoseptoria tritici* following infection bioassays. *Z. tritici* IPO323 strain *Δku70* and its derivatives deficient in RNA silencing pathway (Δ*dcl*, Δ*ago1* or Δ*ago2*) were inoculated onto the wheat cv. Bobwhite. Three randomly chosen mutant strains for each target gene were tested by inoculation onto 3-5 individual wheat seedlings. Fungal inoculations were done using suspension of conidiospores at 5 x 10^6 mL^{-1}. Mock leaves were inoculated with 0.1% Silwet L-77 only. The inoculated and mock-inoculated leaves were then excised and incubated for 48 hr under ~ 100% humidity to induce pycnidiation before being photographed.

A

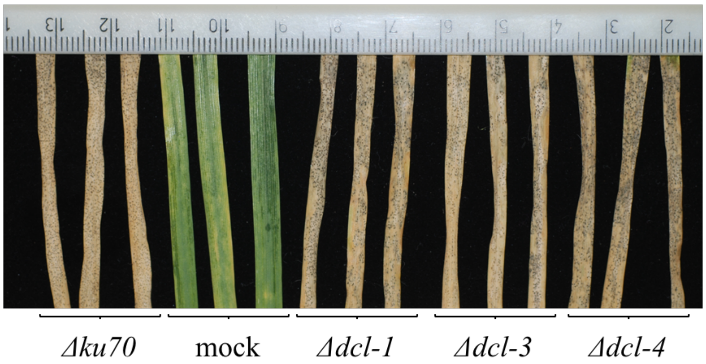

B

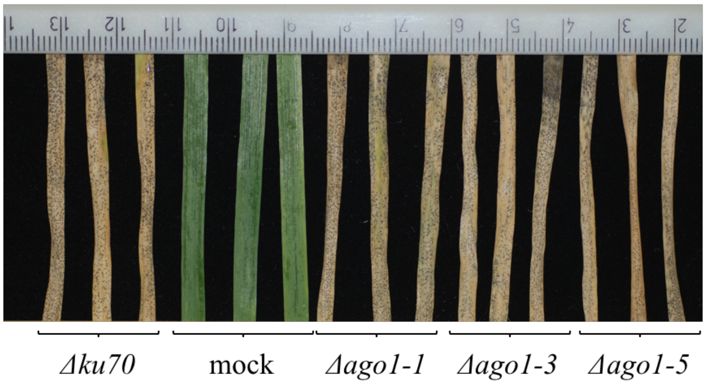

C

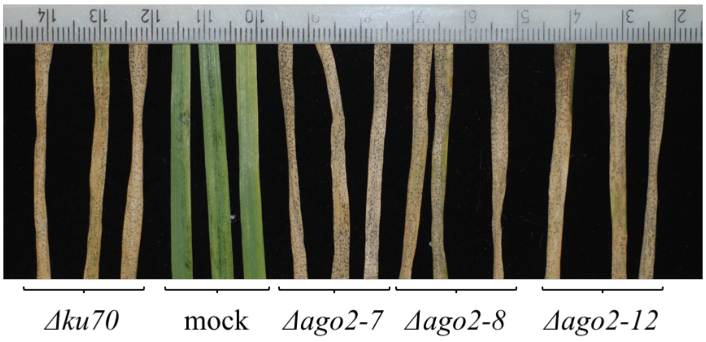

Figure S7. Expression of wheat mRNA Traes_4BS_5E12F0B27 in leaf tissue at 4, 9, and 13 days post inoculation (dpi) with RNAi-competent and RNAi-deficient *Zymoseptoria tritici* strains. Bars indicate SE.

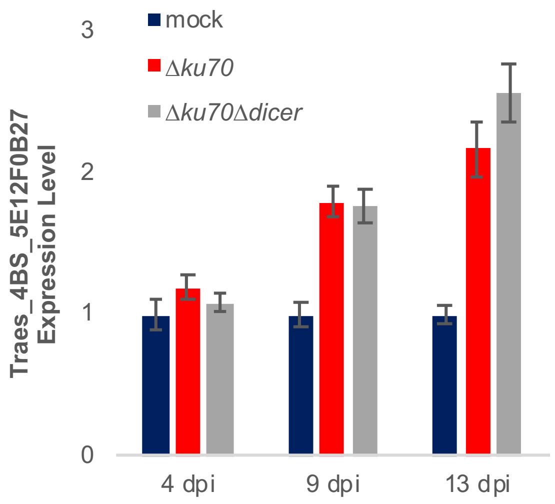

Figure S8. 5'-RACE on the selected wheat targets. Assays were performed using both mock-infected (-) and *Zymoseptoria tritici* IPO323-infected (+) wheat cDNA as templates. Reaction products from lanes indicated in red were sequenced.

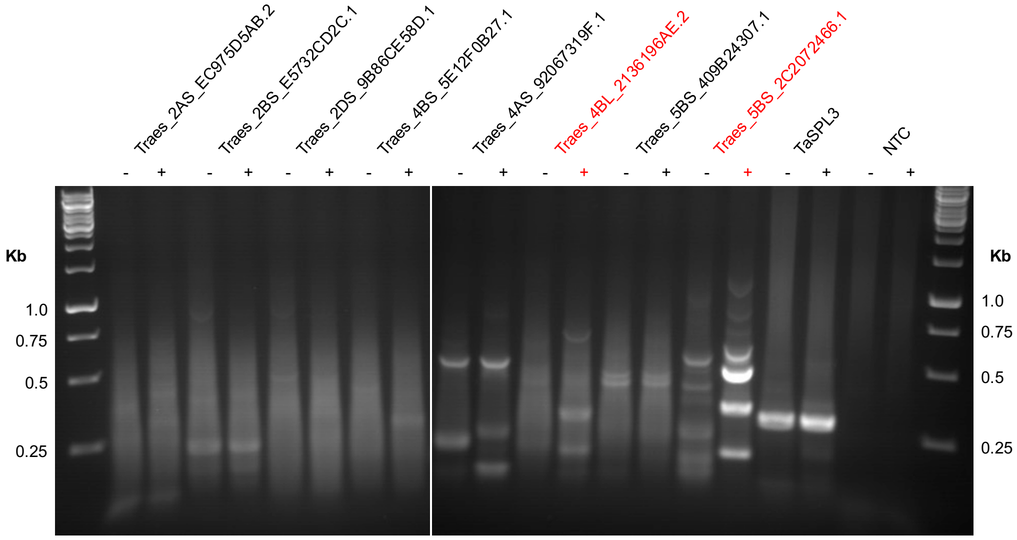

Figure S9. Leaves of wheat cv. Riband plants at 14 days post pre-treatment with the BSMV:asTaMgChlH construct showing RNAi-induced chlorophyll deficiency (orangey-yellow coloration).

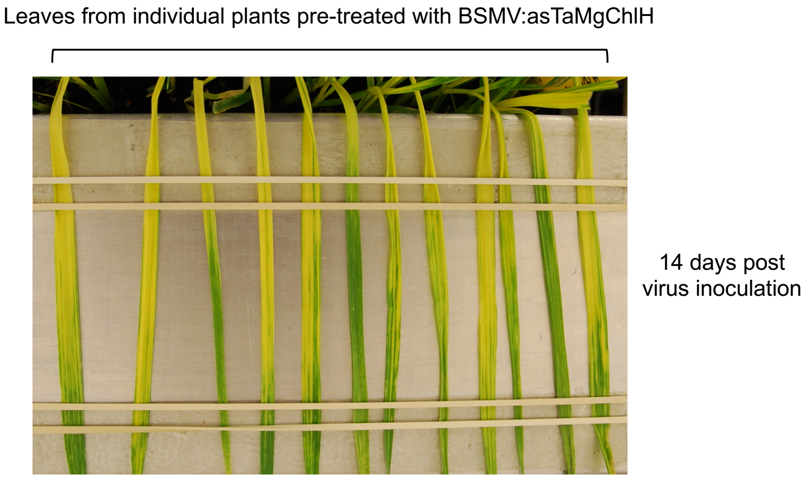

Figure S10. *In vitro* RNAi using externally supplied long dsRNAs targeting essential genes in *Zymoseptoria tritici*. One hundred microliters of *Z. tritici* IPO323 germinating conidiospore suspensions at four different concentrations (indicated on the left) were treated overnight with 12.5 ng (L), 125 ng (M) or 1250 ng (H) of dsRNA specific for different *Z. tritici* mRNAs before plating out onto YPD agar plates and growing for 4 days at 17°C in the dark. CON, untreated *Z. tritici* samples.

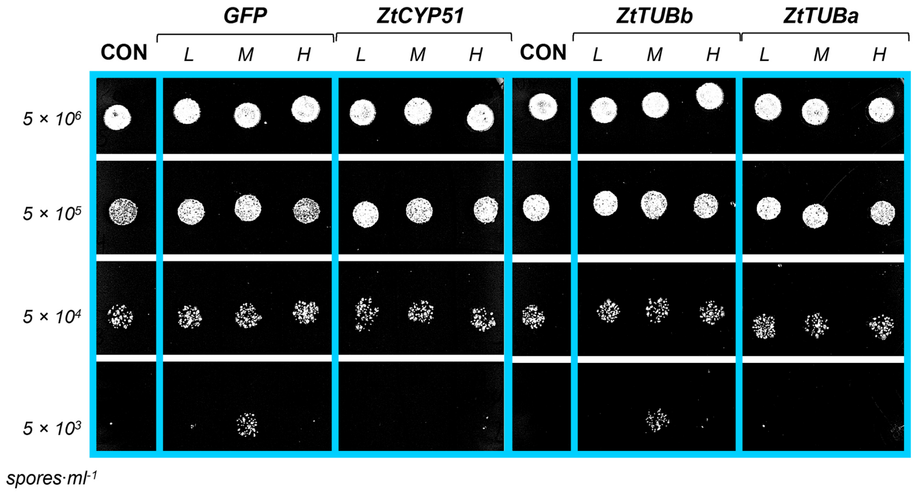

Supplement: Supplementary file 1 [file Data_Sheet_1.PDF]
