# Supplementary material for: sRNA Profiling Combined With Gene Function Analysis Reveals a Lack of Evidence for Cross-Kingdom RNAi in the Wheat – Zymoseptoria tritici Pathosystem
Source: Front Plant Sci. 2019 Jul 4;10:892. doi: 10.3389/fpls.2019.00892 (PMC6620828; doi:10.3389/fpls.2019.00892)
Supplement: Supplementary file 2 [file Data_Sheet_2.PDF]

Table S1. Primers used in this study.

Primer Name	Sequence	Purpose
TamRNA1-1 F	GCGGCTACAAGTTGATGATGAC	qRT-PCR (wheat target mRNA Traes_2AS_EC975D5AB)
TamRNA1-1 R	TCTCCGGACGACCACAATATTG	qRT-PCR (wheat target mRNA Traes_2AS_EC975D5AB)
TamRNA1-2 Fnew	TTCTGTGTGCCTGGATTGTATGTC	qRT-PCR (wheat target mRNA Traes_2BS_E5732CD2C)
TamRNA1-2 Rnew	GAACTTGTATGATGTGTTGAAAGTGGCG	qRT-PCR (wheat target mRNA Traes_2BS_E5732CD2C)
TamRNA1-3 Fnew	TGGAGCCCAGCAAGCACTCGG	qRT-PCR (wheat target mRNA Traes_2DS_9B86CE58D)
TamRNA1-3 Rnew	GCTTGTAATGCAGGGCACAACG	qRT-PCR (wheat target mRNA Traes_2DS_9B86CE58D)
TamRNA 2Fnew	CCGTAACATTCACTGGGTATAAATC	qRT-PCR (wheat target mRNA Traes_4BS_5E12F0B27)
TamRNA 2 Rnew	AAATATACTCTAGATGCACCAGGC	qRT-PCR (wheat target mRNA Traes_4BS_5E12F0B27)
TamRNA3-1 F	CCTTCCTCTCGGTGGGTGTTG	qRT-PCR (wheat target mRNA Traes_4AS_92067319F)
TamRNA 3-1 R	CCCATCTTTGCAGCGCCTGCT	qRT-PCR (wheat target mRNA Traes_4AS_92067319F)
TamRNA 3-2 Fnew	TGAACAGAACTGTATCTGCTGTAAG	qRT-PCR (wheat target mRNA Traes_4BL_2136196AE)
TamRNA 3-2 R	ATGCGGCGGCGGGTCATGG	qRT-PCR (wheat target mRNA Traes_4BL_2136196AE)
TamRNA4-1 Fnew	TCAGTCGCGTACCTTCTTGTC	qRT-PCR (wheat target mRNA Traes_5BS_409B24307)
TamRNA4-1 Rnew	CGAAGTGTTCTGACAAAGCCG	qRT-PCR (wheat target mRNA Traes_5BS_409B24307)
TamRNA4-2 F	CATTTAGGCGCCAGAGAATAATGC	qRT-PCR (wheat target mRNA Traes_5BS_2C2072466)
TamRNA4-2R	GACAGGAACAGGTACTCCCATG	qRT-PCR (wheat target mRNA Traes_5BS_2C2072466)
TaCDC48 newF	GTCCTCCTGGCTGTGGTAAAAC	wheat reference gene for plant tissue qPCR
TaCDC48 newR	AGCAGCTCAGGTCCCTTGATAC	wheat reference gene for plant tissue qPCR
ZtB-tubulin F	CCTACTTCGTCGAGTGGATT	*Z. tritici* reference gene for fungal tissue stem-loop qPCR
ZtB-tubulin R	TACTCGGACACAAGATCGTT	*Z. tritici* reference gene for fungal tissue stem-loop qPCR
universal R	GTGCAGGGTCCGAGGT	stem-loop qRT-PCR (universal reverse primer for all sRNA targets)
Zt-sRNA1 F	GCGATCTTGGGGAATCCGTAG	stem-loop qRT-PCR
Zt-sRNA1 SLRT	GTCGTATCCAGTGCAGGGTCCGAGGTATTCGCACTGGATACGACACAACA	stem-loop cDNA synthesis
Zt-sRNA2 F	TGGACTGCACTGGTTGCTCG	stem-loop qRT-PCR
Zt-sRNA2 SLRT	GTCGTATCCAGTGCAGGGTCCGAGGTATTCGCACTGGATACGACAGCGTT	stem-loop cDNA synthesis
Zt-sRNA3 F	GGCAGTAACCATCTTTCGGGT	stem-loop qRT-PCR
Zt-sRNA3 SLRT	GTCGTATCCAGTGCAGGGTCCGAGGTATTCGCACTGGATACGACAGTCAG	stem-loop cDNA synthesis
Zt-sRNA4 F	GTGAACTGGAGATCGCGAAGG	stem-loop qRT-PCR
Zt-sRNA4 SLRT	GTCGTATCCAGTGCAGGGTCCGAGGTATTCGCACTGGATACGACGAACCT	stem-loop cDNA synthesis
ZtDCL-1	GGGCCCGGCGCGCCGAATTCGAGCTGACAACTATCTGATCTATTGCG	Gibson cloning of the KO construct into vector pCHYG
ZtDCL-2	TCAGTTATCGAATATTGTGTTTCGACGACATC	Gibson cloning of the KO construct into vector pCHYG
ZtDCL-3	GTCGAAACACAATATTCGATAACTGATATTGAAGG	Gibson cloning of the KO construct into vector pCHYG
ZtDCL-4	CATCCAACCACCCCTCGAGGTCGACGGTATC	Gibson cloning of the KO construct into vector pCHYG
ZtDCL-5	CGTCGACCTCGAGGGGTGGTTGGATGGTGATTG	Gibson cloning of the KO construct into vector pCHYG
ZtDCL-6	CACGTGGTGGTGGTGGTGGTGGCTAGCGTTAACACGGCCATTCCGTTCACGA	Gibson cloning of the KO construct into vector pCHYG
ZtAGO2-1	GGGCCCGGCGCGCCGAATTCGAGCTGCCTTGGACTCGGACCGG	Gibson cloning of the KO construct into vector pCHYG
ZtAGO2-2	TCAGTTATCGAATGACAGTGCGGTTGGTGGG	Gibson cloning of the KO construct into vector pCHYG
ZtAGO2-3	CAACCGCACTGTCATTCGATAACTGATATTGAAGG	Gibson cloning of the KO construct into vector pCHYG
ZtAGO2-4	GCTGAAGTGACTTCTCGAGGTCGACGGTATC	Gibson cloning of the KO construct into vector pCHYG
ZtAGO2-5	CGTCGACCTCGAGAAGTCACTTCAGCTTCTTTTC	Gibson cloning of the KO construct into vector pCHYG
ZtAGO2-6	CACGTGGTGGTGGTGGTGGTGGCTAGCGTTAACAGGAGAGAGGGATGATACC	Gibson cloning of the KO construct into vector pCHYG
ZtAGO1-1	GGGCCCGGCGCGCCGAATTCGAGCTCTCGAGCCTCCACCACTAC	Gibson cloning of the KO construct into vector pCHYG
ZtAGO1-2	TCAGTTATCGAATGAAGTTGAGGTTGGGTAACG	Gibson cloning of the KO construct into vector pCHYG
ZtAGO1-3	CAACCTCAACTTCATTCGATAACTGATATTGAAGG	Gibson cloning of the KO construct into vector pCHYG
ZtAGO1-4	TCTTCGAGCTCTCCTCGAGGTCGACGGTATC	Gibson cloning of the KO construct into vector pCHYG
ZtAGO1-5	CGTCGACCTCGAGGAGAGCTCGAAGAGAAATC	Gibson cloning of the KO construct into vector pCHYG
ZtAGO1-6	CACGTGGTGGTGGTGGTGGTGGCTAGCGTTAACACTTACCTGTACGCCTATTCAG	Gibson cloning of the KO construct into vector pCHYG
ztTUBalpha_F2	aaggaagtttaaCACCTACCGCAGCCTTTTCCAC	BSMV-HIGS
ztTUBalpha_R2	aaccaccaccaccgtGTGCTGGGTACACGCAGAACTCA	BSMV-HIGS
ztTUBbeta_F1	aaggaagtttaaCACCCAGCAAATCTTCGACCCTA	BSMV-HIGS
ztTUBbeta_R1	aaccaccaccaccgtCTGAACATGGCGGAGAACTGGTC	BSMV-HIGS
ztCYP51_F1	aaggaagtttaaCCGTTTTGGACTCTCGTCTCTT	BSMV-HIGS
ztCYP51_R1	aaccaccaccaccgtCAGTCCTACGTGACCTTGATCG	BSMV-HIGS
ztALG2_F1	aaggaagtttaaCCTCGTGTACTTGCTGTTCACC	BSMV-HIGS
ztALG2_R1	aaccaccaccaccgtCGATTCGCGATCCTCTGTACTA	BSMV-HIGS
ztTUBalpha_T7P_F2	tcctaatacgactcactatagggagCACCTACCGCAGCCTTTTCCAC	in vitro RNAi
ztTUBalpha_T7P_R2	tcctaatacgactcactatagggagGTGCTGGGTACACGCAGAACTCA	in vitro RNAi
ztTUBbeta_T7P_F1	tcctaatacgactcactatagggagCACCCAGCAAATCTTCGACCCTA	in vitro RNAi
ztTUBbeta_T7P_R1	tcctaatacgactcactatagggagCTGAACATGGCGGAGAACTGGTC	in vitro RNAi
ztCYP51_T7P_F1	tcctaatacgactcactatagggagCCGTTTTGGACTCTCGTCTCTT	in vitro RNAi
ztCYP51_T7P_R1	tcctaatacgactcactatagggagCAGTCCTACGTGACCTTGATCG	in vitro RNAi
GFP_T7P_F1	tcctaatacgactcactatagggagGCACAAATTTTCTGTCAGTGGA	in vitro RNAi
GFP_T7P_R1	tcctaatacgactcactatagggagGTCCGAGAATGTTTCCATCTTC	in vitro RNAi

Table S2. Expression levels of the key predicted RNAi machinery components in *Zymoseptoria tritici* isolate IPO323.

			Mean FPKM values†						
**Gene**	**Ensembl Fungi gene code**	**Locus**	**CDB‡**	**PDB**	**1 dpi**	**4 dpi**	**9 dpi**	**14 dpi**	**21 dpi**
ZtDCL1	Mycgr3G47983	9:1660746-1665410	1.3	1.7	1.0	0.7	1.1	1.3	2.2
ZtAGO1	Mycgr3G38035	3:2630539-2633932	8.9	3.0	14.1	5.4	18.8	11.7	18.1
ZtAGO2	Mycgr3G10621	11:940972-943399	4.0	2.7	4.8	5.1	3.7	2.6	3.4
pseudoAGO3	Mycgr3G90232	1:5667618-5671029	0.1	0.2	0.3	0.8	0.4	0.1	0.2
pseudoAGO4	Mycgr3G25632	1:4995448-4996504	1.3	1.3	7.2	0.0	2.0	2.0	1.0
ZtRDRP1	Mycgr3G51407	13:605705-608630	1.0	0.9	1.5	1.6	1.3	0.7	0.4
ZtRDRP2	Mycgr3G49833	11:202398-206395	0.6	0.5	0.2	0.6	1.4	1.2	1.4

† Expression levels of the candidate genes was inferred based on the previously published RNAseq data (Rudd *et al.,* 2015) and is shown as FPKM (fragments per kilobase of exon model per million reads mapped) values

‡ *Zymoseptoria tritici* isolate IPO323 was grown in vitro in low nutrients CDB (Czapek Dox Broth) or high nutrients PDB (Potato Dextrose Broth) media. The same fungal isolate was also inoculated onto the leaves of susceptible wheat cv. Riband and gene expression assessed at 1, 4, 9, 14, and 21 dpi (days post inoculation).

Table S3. Alignment rate of sRNA sequencing reads to the *Zymoseptoria tritici* (*Zt*) isolate IPO323 genome from 30 *in vitro* and *in planta* samples.

Sample name	Sample description	Number of input reads	Alignment rate (%)
BW0-1	Healthy uninoculated control wheat cv. Bobwhite	1,713,836	3.44
BW0-2	Healthy uninoculated control wheat cv. Bobwhite	6,139,812	1.72
BW0-3	Healthy uninoculated control wheat cv. Bobwhite	5,744,866	1.44
CDB-1	Fungus cultured *in vitro* (in Czapek-Dox Broth)	5,962,085	61.12
CDB-2	Fungus cultured *in vitro* (in Czapek-Dox Broth)	5,489,439	52.46
CDB-3	Fungus cultured *in vitro* (in Czapek-Dox Broth)	5,804,472	51.79
BWM4-1	Wheat cv. Bobwhite sampled at 4 days post mock inoculation	4,929,559	2.20
BWM4-2	Wheat cv. Bobwhite sampled at 4 days post mock inoculation	2,898,526	2.67
BWM4-3	Wheat cv. Bobwhite sampled at 4 days post mock inoculation	4,774,689	3.25
BWZt4-1	Wheat cv. Bobwhite sampled at 4 days post fungal inoculation	6,227,127	5.70
BWZt4-2	Wheat cv. Bobwhite sampled at 4 days post fungal inoculation	6,395,321	2.92
BWZt4-3	Wheat cv. Bobwhite sampled at 4 days post fungal inoculation	4,939,622	4.11
BWM9-1	Wheat cv. Bobwhite sampled at 9 days post mock inoculation	4,841,465	1.89
BWM9-2	Wheat cv. Bobwhite sampled at 9 days post mock inoculation	4,431,331	1.46
BWM9-3	Wheat cv. Bobwhite sampled at 9 days post mock inoculation	3,717,393	1.97
BWZt9-1	Wheat cv. Bobwhite sampled at 9 days post fungal inoculation	6,718,653	7.18
BWZt9-2	Wheat cv. Bobwhite sampled at 9 days post fungal inoculation	5,846,043	6.40
BWZt9-3	Wheat cv. Bobwhite sampled at 9 days post fungal inoculation	6,381,610	6.68
BWM13-1	Wheat cv. Bobwhite sampled at 13 days post mock inoculation	5,279,980	2.16
BWM13-2	Wheat cv. Bobwhite sampled at 13 days post mock inoculation	5,751,607	2.97
BWM13-3	Wheat cv. Bobwhite sampled at 13 days post mock inoculation	1,937,591	2.98
BWZt13-1	Wheat cv. Bobwhite sampled at 13 days post fungal inoculation	5,053,219	15.28
BWZt13-2	Wheat cv. Bobwhite sampled at 13 days post fungal inoculation	4,190,908	25.60
BWZt13-3	Wheat cv. Bobwhite sampled at 13 days post fungal inoculation	4,203,443	19.27
BWM21-1	Wheat cv. Bobwhite sampled at 21 days post mock inoculation	5,379,386	2.88
BWM21-2	Wheat cv. Bobwhite sampled at 21 days post mock inoculation	5,422,889	2.40
BWM21-3	Wheat cv. Bobwhite sampled at 21 days post mock inoculation	2,716,384	1.89
BWZt21-1	Wheat cv. Bobwhite sampled at 21 days post fungal inoculation	4,426,578	35.13
BWZt21-2	Wheat cv. Bobwhite sampled at 21 days post fungal inoculation	3,446,552	31.81
BWZt21-3	Wheat cv. Bobwhite sampled at 21 days post fungal inoculation	2,982,932	27.04

Table S4. Computationally predicted *Zymoseptoria tritici* IPO323 sRNA loci.

sRNA locus ID	chromosome	start (nt)	end (nt)	length (nt)	sRNA read counts from all samples	overlaps with protein coding genes	overlaps with other genes	overlaps with TE / repetitive elements	sRNA loci active at 4, 9, 13, or 21 dpi during wheat infection				posterior probability difference†	Min FDR differential expression‡
1.2.24760	1	2	24760	24759	29600			yes	BWZt4	BWZt9	BWZt13	BWZt21	0.99	0.00
1.24832.33907	1	24832	33907	9076	7406			yes	BWZt4	BWZt9	BWZt13	BWZt21	1.00	0.00
1.52723.55652	1	52723	55652	2930	2665			yes		BWZt9	BWZt13	BWZt21	1.00	0.00
1.55724.59340	1	55724	59340	3617	3434			yes		BWZt9	BWZt13	BWZt21	1.00	0.00
1.59399.69532	1	59399	69532	10134	7238			yes		BWZt9	BWZt13	BWZt21	1.00	0.00
1.132780.135800	1	132780	135800	3021	498	yes					BWZt13	BWZt21	1.00	0.01
1.168753.169514	1	168753	169514	762	7814			yes	BWZt4	BWZt9	BWZt13	BWZt21	1.00	0.00
1.610884.611019	1	610884	611019	136	2523					BWZt9	BWZt13	BWZt21	1.00	0.00
1.681033.681226	1	681033	681226	194	1345	yes				BWZt9	BWZt13	BWZt21	1.00	0.00
1.686410.686631	1	686410	686631	222	872	yes						BWZt21	1.00	0.04
1.802150.802635	1	802150	802635	486	4664						BWZt13		1.00	0.00
1.809089.809248	1	809089	809248	160	1207						BWZt13		1.00	0.01
1.912518.913393	1	912518	913393	876	5584			yes		BWZt9	BWZt13	BWZt21	1.00	0.00
1.952268.954112	1	952268	954112	1845	2446	yes					BWZt13	BWZt21	1.00	0.00
1.1088031.1088594	1	1088031	1088594	564	938				BWZt4	BWZt9		BWZt21	1.00	0.00
1.1432722.1433785	1	1432722	1433785	1064	6332	yes		yes	BWZt4	BWZt9	BWZt13	BWZt21	1.00	0.00
1.2088218.2090311	1	2088218	2090311	2094	525	yes						BWZt21	1.00	0.02
1.2630102.2631752	1	2630102	2631752	1651	1029	yes						BWZt21	1.00	0.00
1.3024339.3025761	1	3024339	3025761	1423	1546	yes					BWZt13	BWZt21	1.00	0.02
1.3107284.3109571	1	3107284	3109571	2288	17700	yes		yes	BWZt4	BWZt9	BWZt13	BWZt21	1.00	0.00
1.3175256.3175844	1	3175256	3175844	589	2550	yes		yes	BWZt4	BWZt9	BWZt13	BWZt21	1.00	0.00
1.3404853.3405015	1	3404853	3405015	163	648000		5S rRNA		BWZt4	BWZt9	BWZt13	BWZt21	1.00	0.00
1.3788931.3792998	1	3788931	3792998	4068	470	yes						BWZt21	1.00	0.02
1.3817478.3818480	1	3817478	3818480	1003	282	yes						BWZt21	1.00	0.02
1.3864242.3867547	1	3864242	3867547	3306	423	yes						BWZt21	1.00	0.01
1.4059657.4059846	1	4059657	4059846	190	840						BWZt13	BWZt21	1.00	0.01
1.4098242.4102440	1	4098242	4102440	4199	762	yes					BWZt13	BWZt21	1.00	0.00
1.4411833.4412084	1	4411833	4412084	252	31300				BWZt4	BWZt9	BWZt13	BWZt21	1.00	0.00
1.4420671.4420786	1	4420671	4420786	116	2793	yes					BWZt13		1.00	0.00
1.4461121.4461429	1	4461121	4461429	309	2637		tRNA-Arg		BWZt4	BWZt9	BWZt13	BWZt21	1.00	0.00
1.4500965.4502701	1	4500965	4502701	1737	872	yes					BWZt13		1.00	0.02
1.4519579.4520495	1	4519579	4520495	917	6544			yes	BWZt4	BWZt9	BWZt13	BWZt21	1.00	0.00
1.4532087.4532257	1	4532087	4532257	171	2776		5S rRNA		BWZt4	BWZt9	BWZt13	BWZt21	1.00	0.00
1.4859784.4862055	1	4859784	4862055	2272	1326	yes						BWZt21	1.00	0.01
1.5027663.5030714	1	5027663	5030714	3052	1058	yes					BWZt13	BWZt21	1.00	0.00
1.5208279.5209283	1	5208279	5209283	1005	6982			yes	BWZt4	BWZt9	BWZt13	BWZt21	1.00	0.00
1.5370559.5371316	1	5370559	5371316	758	1768	yes						BWZt21	1.00	0.01
1.5574650.5575522	1	5574650	5575522	873	9652						BWZt13	BWZt21	1.00	0.00
1.5935918.5953168	1	5935918	5953168	17251	12900	yes					BWZt13		1.00	0.00
1.6022523.6024517	1	6022523	6024517	1995	2563			yes		BWZt9	BWZt13	BWZt21	1.00	0.00
1.6024577.6027831	1	6024577	6027831	3255	1941			yes		BWZt9	BWZt13	BWZt21	1.00	0.00
1.6042189.6044283	1	6042189	6044283	2095	2270			yes		BWZt9	BWZt13	BWZt21	1.00	0.00
1.6044579.6052106	1	6044579	6052106	7528	5140			yes		BWZt9	BWZt13	BWZt21	1.00	0.00
1.6052501.6055814	1	6052501	6055814	3314	1568			yes		BWZt9	BWZt13	BWZt21	1.00	0.00
1.6055877.6056728	1	6055877	6056728	852	487			yes		BWZt9	BWZt13	BWZt21	1.00	0.01
1.6060199.6062484	1	6060199	6062484	2286	970			yes		BWZt9	BWZt13	BWZt21	1.00	0.00
1.6062562.6077812	1	6062562	6077812	15251	13200			yes		BWZt9	BWZt13	BWZt21	1.00	0.00
1.6077865.6084551	1	6077865	6084551	6687	4298			yes		BWZt9	BWZt13	BWZt21	1.00	0.00
2.17721.18726	2	17721	18726	1006	6063			yes		BWZt9	BWZt13	BWZt21	1.00	0.00
2.47729.49709	2	47729	49709	1981	7360			yes	BWZt4	BWZt9	BWZt13	BWZt21	1.00	0.00
2.236665.237674	2	236665	237674	1010	8376	yes		yes	BWZt4	BWZt9	BWZt13	BWZt21	1.00	0.00
2.341988.345005	2	341988	345005	3018	17800	yes		yes	BWZt4	BWZt9	BWZt13	BWZt21	1.00	0.00
2.400134.400399	2	400134	400399	266	6122				BWZt4	BWZt9	BWZt13	BWZt21	1.00	0.00
2.532319.532572	2	532319	532572	254	648000		5S rRNA		BWZt4	BWZt9	BWZt13	BWZt21	1.00	0.00
2.715507.715678	2	715507	715678	172	2319						BWZt13		1.00	0.01
2.819512.820307	2	819512	820307	796	5263			yes	BWZt4	BWZt9	BWZt13	BWZt21	1.00	0.00
2.1061972.1062929	2	1061972	1062929	958	9003			yes	BWZt4	BWZt9	BWZt13	BWZt21	1.00	0.00
2.1128831.1132146	2	1128831	1132146	3316	1909	yes				BWZt9	BWZt13	BWZt21	1.00	0.00
2.1295279.1297239	2	1295279	1297239	1961	338	yes						BWZt21	1.00	0.01
2.1326532.1328523	2	1326532	1328523	1992	721	yes					BWZt13		1.00	0.03
2.1333569.1334006	2	1333569	1334006	438	2840						BWZt13	BWZt21	1.00	0.00
2.1441675.1441950	2	1441675	1441950	276	70400		tRNA-Ser		BWZt4	BWZt9	BWZt13	BWZt21	1.00	0.00
2.1664358.1664628	2	1664358	1664628	271	513						BWZt13	BWZt21	1.00	0.00
2.1741314.1742939	2	1741314	1742939	1626	10100	yes		yes	BWZt4	BWZt9	BWZt13	BWZt21	1.00	0.00
2.1895220.1896101	2	1895220	1896101	882	3764			yes	BWZt4	BWZt9	BWZt13	BWZt21	1.00	0.00
2.1903263.1903455	2	1903263	1903455	193	648000		5S rRNA		BWZt4	BWZt9	BWZt13	BWZt21	1.00	0.00
2.1909278.1910404	2	1909278	1910404	1127	6408			yes	BWZt4	BWZt9	BWZt13	BWZt21	1.00	0.00
2.1925528.1927912	2	1925528	1927912	2385	279	yes						BWZt21	1.00	0.03
2.2093476.2093705	2	2093476	2093705	230	16200	yes			BWZt4		BWZt13		1.00	0.00
2.2362398.2364568	2	2362398	2364568	2171	454	yes						BWZt21	1.00	0.00
2.2743117.2744701	2	2743117	2744701	1585	281	yes						BWZt21	1.00	0.02
2.2748785.2750628	2	2748785	2750628	1844	1124	yes						BWZt21	1.00	0.01
2.2777908.2780777	2	2777908	2780777	2870	1254	yes						BWZt21	0.99	0.02
2.2865736.2866983	2	2865736	2866983	1248	1445	yes				BWZt9			1.00	0.03
2.3153789.3154883	2	3153789	3154883	1095	1498	yes					BWZt13		1.00	0.00
2.3187737.3188483	2	3187737	3188483	747	6216			yes	BWZt4	BWZt9	BWZt13	BWZt21	1.00	0.00
2.3189040.3189775	2	3189040	3189775	736	7256			yes	BWZt4	BWZt9	BWZt13	BWZt21	1.00	0.00
2.3276658.3277710	2	3276658	3277710	1053	8657			yes	BWZt4	BWZt9	BWZt13	BWZt21	1.00	0.00
2.3411312.3412084	2	3411312	3412084	773	2302					BWZt9	BWZt13	BWZt21	1.00	0.00
2.3632877.3633814	2	3632877	3633814	938	4419			yes	BWZt4	BWZt9	BWZt13	BWZt21	1.00	0.00
2.3801864.3816890	2	3801864	3816890	15027	15300			yes	BWZt4	BWZt9	BWZt13	BWZt21	1.00	0.00
2.3816969.3821391	2	3816969	3821391	4423	3528			yes		BWZt9	BWZt13	BWZt21	1.00	0.00
2.3821445.3822997	2	3821445	3822997	1553	596			yes		BWZt9	BWZt13	BWZt21	1.00	0.00
2.3823060.3839541	2	3823060	3839541	16482	16200		tRNA-Cys	yes		BWZt9	BWZt13	BWZt21	1.00	0.00
2.3839620.3844042	2	3839620	3844042	4423	3603			yes		BWZt9	BWZt13	BWZt21	1.00	0.00
2.3844121.3848381	2	3844121	3848381	4261	1931		tRNA-Cys	yes		BWZt9	BWZt13	BWZt21	1.00	0.00
3.1178.7084	3	1178	7084	5907	3149			yes		BWZt9	BWZt13	BWZt21	1.00	0.00
3.7151.18710	3	7151	18710	11560	12200			yes	BWZt4	BWZt9	BWZt13	BWZt21	1.00	0.00
3.218432.218715	3	218432	218715	284	1229		tRNA-Tyr					BWZt21	1.00	0.00
3.387886.388363	3	387886	388363	478	867						BWZt13	BWZt21	1.00	0.01
3.391037.392727	3	391037	392727	1691	5811			yes	BWZt4	BWZt9	BWZt13	BWZt21	1.00	0.00
3.401660.407274	3	401660	407274	5615	5891			yes		BWZt9	BWZt13	BWZt21	1.00	0.00
3.448737.450457	3	448737	450457	1721	379	yes						BWZt21	1.00	0.01
3.451342.453408	3	451342	453408	2067	395	yes						BWZt21	1.00	0.00
3.587789.590578	3	587789	590578	2790	8793			yes	BWZt4	BWZt9	BWZt13	BWZt21	1.00	0.00
3.699143.699357	3	699143	699357	215	4222		tRNA-Val				BWZt13	BWZt21	1.00	0.00
3.759692.759839	3	759692	759839	148	1314					BWZt9	BWZt13	BWZt21	1.00	0.00
3.822679.823459	3	822679	823459	781	8275			yes	BWZt4	BWZt9	BWZt13	BWZt21	1.00	0.00
3.957606.958577	3	957606	958577	972	6221			yes	BWZt4	BWZt9	BWZt13	BWZt21	1.00	0.00
3.1848836.1849733	3	1848836	1849733	898	6302			yes	BWZt4	BWZt9	BWZt13	BWZt21	1.00	0.00
3.1987288.1987894	3	1987288	1987894	607	2124							BWZt21	1.00	0.01
3.2136686.2136750	3	2136686	2136750	65	331		snRNA		BWZt4				1.00	0.01
3.2252943.2253304	3	2252943	2253304	362	3819						BWZt13	BWZt21	1.00	0.00
3.2292170.2292823	3	2292170	2292823	654	248				BWZt4				1.00	0.05
3.2312849.2313471	3	2312849	2313471	623	1603		tRNA-Cys	yes				BWZt21	1.00	0.05
3.2375162.2376724	3	2375162	2376724	1563	3326	yes					BWZt13		1.00	0.00
3.2469896.2470926	3	2469896	2470926	1031	550							BWZt21	1.00	0.04
3.2711296.2712607	3	2711296	2712607	1312	508	yes						BWZt21	1.00	0.00
3.2736368.2736981	3	2736368	2736981	614	5916				BWZt4		BWZt13	BWZt21	1.00	0.00
3.2779587.2780080	3	2779587	2780080	494	594						BWZt13		1.00	0.02
3.2791693.2793471	3	2791693	2793471	1779	523	yes			BWZt4			BWZt21	1.00	0.03
3.3048306.3049442	3	3048306	3049442	1137	1168	yes					BWZt13	BWZt21	1.00	0.00

3.3241153.3242334	3	3241153	3242334	1182	651000	yes	5S rRNA		BWZt4	BWZt9	BWZt13	BWZt21	1.00	0.00
3.3403412.3404238	3	3403412	3404238	827	8607			yes	BWZt4	BWZt9	BWZt13	BWZt21	1.00	0.00
3.3468771.3476338	3	3468771	3476338	7568	2969			yes		BWZt9	BWZt13	BWZt21	1.00	0.00
3.3486626.3493394	3	3486626	3493394	6769	1790			yes		BWZt9	BWZt13	BWZt21	1.00	0.00
3.3496452.3502010	3	3496452	3502010	5559	1689	yes		yes		BWZt9	BWZt13	BWZt21	1.00	0.00
4.8404.10428	4	8404	10428	2025	1273			yes		BWZt9	BWZt13	BWZt21	1.00	0.00
4.10488.12213	4	10488	12213	1726	565			yes			BWZt13	BWZt21	1.00	0.01
4.170472.171758	4	170472	171758	1287	7978			yes	BWZt4	BWZt9	BWZt13	BWZt21	1.00	0.00
4.234947.235725	4	234947	235725	779	6692			yes	BWZt4	BWZt9	BWZt13	BWZt21	1.00	0.00
4.241532.242398	4	241532	242398	867	7016			yes		BWZt9	BWZt13	BWZt21	1.00	0.00
4.272845.274005	4	272845	274005	1161	6626			yes	BWZt4	BWZt9	BWZt13	BWZt21	1.00	0.00
4.356163.358445	4	356163	358445	2283	577	yes						BWZt21	1.00	0.02
4.595344.597737	4	595344	597737	2394	796	yes						BWZt21	1.00	0.02
4.919278.919425	4	919278	919425	148	4403		tRNA-Arg		BWZt4	BWZt9	BWZt13	BWZt21	1.00	0.00
4.1251113.1252024	4	1251113	1252024	912	7241			yes	BWZt4	BWZt9	BWZt13	BWZt21	1.00	0.00
4.1428859.1429718	4	1428859	1429718	860	6218			yes	BWZt4	BWZt9	BWZt13	BWZt21	1.00	0.00
4.2028875.2030805	4	2028875	2030805	1931	3750	yes			BWZt4	BWZt9		BWZt21	1.00	0.00
4.2215448.2215980	4	2215448	2215980	533	1231				BWZt4	BWZt9		BWZt21	1.00	0.00
4.2228316.2228727	4	2228316	2228727	412	5160						BWZt13	BWZt21	1.00	0.00
4.2261891.2262208	4	2261891	2262208	318	780						BWZt13		1.00	0.02
4.2562105.2562351	4	2562105	2562351	247	14200		tRNA-Cys	yes	BWZt4	BWZt9	BWZt13	BWZt21	1.00	0.00
4.2644748.2645586	4	2644748	2645586	839	7707			yes	BWZt4	BWZt9	BWZt13	BWZt21	1.00	0.00
4.2866934.2868658	4	2866934	2868658	1725	5776			yes	BWZt4	BWZt9	BWZt13	BWZt21	1.00	0.00
4.2869853.2880010	4	2869853	2880010	10158	6478			yes		BWZt9	BWZt13		1.00	0.00
5.59060.61544	5	59060	61544	2485	1647			yes		BWZt9	BWZt13	BWZt21	1.00	0.00
5.181519.182321	5	181519	182321	803	7098			yes	BWZt4	BWZt9	BWZt13	BWZt21	1.00	0.00
5.250300.250376	5	250300	250376	77	680	yes					BWZt13		1.00	0.00
5.308748.309026	5	308748	309026	279	358							BWZt21	1.00	0.03
5.514262.514929	5	514262	514929	668	648000	yes	5S rRNA		BWZt4	BWZt9	BWZt13	BWZt21	1.00	0.00
5.523622.524805	5	523622	524805	1184	5827			yes	BWZt4	BWZt9	BWZt13	BWZt21	1.00	0.00
5.557661.560726	5	557661	560726	3066	17900	yes		yes	BWZt4	BWZt9	BWZt13	BWZt21	1.00	0.00
5.561012.563057	5	561012	563057	2046	534	yes						BWZt21	1.00	0.01
5.748438.748459	5	748438	748459	22	4191	yes				BWZt9	BWZt13	BWZt21	1.00	0.00
5.849612.850341	5	849612	850341	730	367	yes						BWZt21	1.00	0.01
5.912353.914719	5	912353	914719	2367	399	yes			BWZt4				1.00	0.02
5.918436.919203	5	918436	919203	768	7368			yes	BWZt4	BWZt9	BWZt13	BWZt21	1.00	0.00
5.934505.936848	5	934505	936848	2344	6860			yes	BWZt4	BWZt9	BWZt13	BWZt21	1.00	0.00
5.1045197.1045805	5	1045197	1045805	609	3609		snRNA		BWZt4	BWZt9	BWZt13		1.00	0.00
5.1092026.1092263	5	1092026	1092263	238	593					BWZt9	BWZt13		1.00	0.00
5.1092560.1092727	5	1092560	1092727	168	7446		snoRNA		BWZt4				1.00	0.00
5.1098642.1098834	5	1098642	1098834	193	7171				BWZt4	BWZt9	BWZt13	BWZt21	1.00	0.00
5.1130578.1135063	5	1130578	1135063	4486	1317	yes					BWZt13	BWZt21	1.00	0.00
5.1875780.1875912	5	1875780	1875912	133	2761				BWZt4	BWZt9	BWZt13	BWZt21	1.00	0.00
5.2094726.2095999	5	2094726	2095999	1274	566	yes					BWZt13	BWZt21	1.00	0.00
5.2318983.2319321	5	2318983	2319321	339	11400				BWZt4	BWZt9	BWZt13	BWZt21	1.00	0.00
5.2486971.2487211	5	2486971	2487211	241	3039		tRNA					BWZt21	1.00	0.04
5.2645459.2646021	5	2645459	2646021	563	836			yes				BWZt21	1.00	0.02
5.2686132.2687400	5	2686132	2687400	1269	9195			yes	BWZt4	BWZt9	BWZt13	BWZt21	1.00	0.00
5.2792872.2793631	5	2792872	2793631	760	138	yes			BWZt4				1.00	0.03
5.2806545.2846948	5	2806545	2846948	40404	33100			yes	BWZt4	BWZt9	BWZt13	BWZt21	1.00	0.00
5.2850297.2861802	5	2850297	2861802	11506	12400			yes		BWZt9	BWZt13	BWZt21	1.00	0.00
6.13518.16550	6	13518	16550	3033	1539			yes		BWZt9	BWZt13	BWZt21	1.00	0.00
6.16617.39543	6	16617	39543	22927	21100			yes	BWZt4	BWZt9	BWZt13	BWZt21	1.00	0.00
6.39611.43211	6	39611	43211	3601	4096			yes		BWZt9	BWZt13	BWZt21	1.00	0.00
6.173879.174893	6	173879	174893	1015	7114			yes		BWZt9	BWZt13	BWZt21	1.00	0.00
6.212702.213658	6	212702	213658	957	4395			yes	BWZt4	BWZt9	BWZt13	BWZt21	1.00	0.00
6.218141.218388	6	218141	218388	248	1012						BWZt13	BWZt21	1.00	0.00
6.231370.231410	6	231370	231410	41	303		miRNA		BWZt4	BWZt9			1.00	0.01
6.597638.598424	6	597638	598424	787	6271			yes	BWZt4	BWZt9	BWZt13	BWZt21	1.00	0.00
6.673455.674376	6	673455	674376	922	6257			yes	BWZt4	BWZt9	BWZt13	BWZt21	1.00	0.00
6.782298.789507	6	782298	789507	7210	8041			yes	BWZt4	BWZt9	BWZt13	BWZt21	1.00	0.00
6.804863.807140	6	804863	807140	2278	7530			yes	BWZt4	BWZt9	BWZt13	BWZt21	1.00	0.00
6.927640.928434	6	927640	928434	795	4317			yes	BWZt4	BWZt9	BWZt13	BWZt21	1.00	0.00
6.999529.999949	6	999529	999949	421	6710			yes	BWZt4	BWZt9	BWZt13	BWZt21	1.00	0.00
6.1267883.1270184	6	1267883	1270184	2302	1486			yes		BWZt9	BWZt13	BWZt21	1.00	0.00
6.1808235.1808688	6	1808235	1808688	454	11300		tRNA-Cys	yes	BWZt4	BWZt9	BWZt13	BWZt21	1.00	0.00
6.2022916.2027069	6	2022916	2027069	4154	9928			yes	BWZt4	BWZt9	BWZt13	BWZt21	1.00	0.00
6.2108904.2109094	6	2108904	2109094	191	3494		snRNA				BWZt13	BWZt21	1.00	0.00
6.2197358.2198045	6	2197358	2198045	688	1025					BWZt9	BWZt13	BWZt21	1.00	0.00
6.2414949.2415730	6	2414949	2415730	782	6236			yes	BWZt4	BWZt9	BWZt13	BWZt21	1.00	0.00
7.64956.65152	7	64956	65152	197	651000		5S rRNA		BWZt4	BWZt9	BWZt13	BWZt21	1.00	0.00
7.95108.95160	7	95108	95160	53	7758					BWZt9	BWZt13	BWZt21	1.00	0.00
7.127872.128627	7	127872	128627	756	7253			yes	BWZt4	BWZt9	BWZt13	BWZt21	1.00	0.00
7.179855.180653	7	179855	180653	799	5162			yes		BWZt9	BWZt13	BWZt21	1.00	0.00
7.299254.300443	7	299254	300443	1190	1324	yes						BWZt21	1.00	0.05
7.452408.453395	7	452408	453395	988	8466			yes	BWZt4	BWZt9	BWZt13	BWZt21	1.00	0.00
7.582594.583749	7	582594	583749	1156	6210			yes	BWZt4	BWZt9	BWZt13	BWZt21	1.00	0.00
7.584106.585040	7	584106	585040	935	7723			yes	BWZt4	BWZt9	BWZt13	BWZt21	1.00	0.00
7.594685.595531	7	594685	595531	847	3772			yes	BWZt4	BWZt9	BWZt13	BWZt21	1.00	0.00
7.755129.758077	7	755129	758077	2949	7841			yes	BWZt4	BWZt9	BWZt13	BWZt21	1.00	0.00
7.826820.827289	7	826820	827289	470	248					BWZt9			1.00	0.04
7.883863.884771	7	883863	884771	909	1091						BWZt13		1.00	0.00
7.927599.928416	7	927599	928416	818	6923			yes	BWZt4	BWZt9	BWZt13	BWZt21	1.00	0.00
7.951736.951950	7	951736	951950	215	854						BWZt13	BWZt21	1.00	0.00
7.1165974.1167274	7	1165974	1167274	1301	4533	yes			BWZt4		BWZt13	BWZt21	1.00	0.00
7.1263898.1264763	7	1263898	1264763	866	7224			yes	BWZt4	BWZt9	BWZt13	BWZt21	1.00	0.00
7.1351801.1352313	7	1351801	1352313	513	5993				BWZt4	BWZt9	BWZt13	BWZt21	1.00	0.00
7.1677669.1677729	7	1677669	1677729	61	13600		18S rRNA		BWZt4	BWZt9		BWZt21	1.00	0.00
7.1678104.1678175	7	1678104	1678175	72	5575		18S rRNA				BWZt13	BWZt21	1.00	0.00
7.1685939.1685999	7	1685939	1685999	61	13600		18S rRNA		BWZt4	BWZt9		BWZt21	1.00	0.00
7.1686374.1686445	7	1686374	1686445	72	5575		18S rRNA				BWZt13	BWZt21	1.00	0.00
7.1804941.1806212	7	1804941	1806212	1272	7862			yes	BWZt4	BWZt9	BWZt13	BWZt21	1.00	0.00
7.1814849.1815870	7	1814849	1815870	1022	7794			yes	BWZt4	BWZt9	BWZt13	BWZt21	1.00	0.00
7.1870917.1873373	7	1870917	1873373	2457	2167	yes				BWZt9	BWZt13	BWZt21	1.00	0.00
7.2249688.2249714	7	2249688	2249714	27	908					BWZt9	BWZt13	BWZt21	1.00	0.00
7.2536483.2537653	7	2536483	2537653	1171	8978			yes	BWZt4	BWZt9	BWZt13	BWZt21	1.00	0.00
7.2613424.2626681	7	2613424	2626681	13258	13000			yes		BWZt9	BWZt13	BWZt21	1.00	0.00
7.2645542.2651117	7	2645542	2651117	5576	990			yes		BWZt9	BWZt13	BWZt21	1.00	0.00
8.78306.79126	8	78306	79126	821	143	yes						BWZt21	1.00	0.03
8.135097.135842	8	135097	135842	746	7312			yes	BWZt4	BWZt9	BWZt13	BWZt21	1.00	0.00
8.192706.195020	8	192706	195020	2315	528	yes					BWZt13		1.00	0.04
8.420489.421629	8	420489	421629	1141	7561			yes	BWZt4	BWZt9	BWZt13	BWZt21	1.00	0.00
8.558236.559373	8	558236	559373	1138	7380			yes	BWZt4	BWZt9	BWZt13	BWZt21	1.00	0.00
8.564251.565700	8	564251	565700	1450	7114			yes	BWZt4	BWZt9	BWZt13	BWZt21	1.00	0.00
8.821716.821919	8	821716	821919	204	919							BWZt21	1.00	0.01
8.828807.828884	8	828807	828884	78	1147							BWZt21	1.00	0.00
8.829026.829103	8	829026	829103	78	1210							BWZt21	1.00	0.00
8.1105306.1107648	8	1105306	1107648	2343	541	yes						BWZt21	1.00	0.00
8.1152834.1153178	8	1152834	1153178	345	3101		tRNA-His				BWZt13		1.00	0.00
8.1375496.1375926	8	1375496	1375926	431	4085				BWZt4	BWZt9		BWZt21	1.00	0.00
8.1401272.1402008	8	1401272	1402008	737	1823						BWZt13	BWZt21	1.00	0.00
8.1402168.1402594	8	1402168	1402594	427	17300				BWZt4	BWZt9	BWZt13	BWZt21	1.00	0.00
8.1402809.1403198	8	1402809	1403198	390	16800				BWZt4	BWZt9	BWZt13	BWZt21	1.00	0.00
8.1449944.1450478	8	1449944	1450478	535	703				BWZt4				1.00	0.04
8.1496933.1498574	8	1496933	1498574	1642	1560					BWZt9	BWZt13	BWZt21	1.00	0.00
8.1619634.1620112	8	1619634	1620112	479	5534						BWZt13		1.00	0.00

8.1778342.1779282	8	1778342	1779282	941	6727			yes	BWZt4	BWZt9	BWZt13	BWZt21	1.00	0.00
8.1779347.1780499	8	1779347	1780499	1153	7781			yes	BWZt4	BWZt9	BWZt13	BWZt21	1.00	0.00
8.1816353.1818241	8	1816353	1818241	1889	4718				BWZt4	BWZt9	BWZt13	BWZt21	1.00	0.00
8.1826676.1828336	8	1826676	1828336	1661	6470			yes	BWZt4	BWZt9	BWZt13	BWZt21	1.00	0.00
8.1839987.1840797	8	1839987	1840797	811	6638			yes	BWZt4	BWZt9	BWZt13	BWZt21	1.00	0.00
8.1960420.1962344	8	1960420	1962344	1925	814	yes						BWZt21	1.00	0.02
8.1964648.1966838	8	1964648	1966838	2191	7782			yes	BWZt4	BWZt9	BWZt13	BWZt21	1.00	0.00
8.2000570.2000759	8	2000570	2000759	190	648000		5S rRNA		BWZt4	BWZt9	BWZt13	BWZt21	1.00	0.00
8.2011199.2012128	8	2011199	2012128	930	4458			yes	BWZt4	BWZt9	BWZt13	BWZt21	1.00	0.00
8.2142675.2143478	8	2142675	2143478	804	5490			yes	BWZt4	BWZt9	BWZt13	BWZt21	1.00	0.00
8.2168811.2169041	8	2168811	2169041	231	15300		tRNA-Gly			BWZt9		BWZt21	1.00	0.00
8.2202983.2203732	8	2202983	2203732	750	6510			yes	BWZt4	BWZt9	BWZt13	BWZt21	1.00	0.00
8.2292152.2298576	8	2292152	2298576	6425	7366			yes	BWZt4	BWZt9	BWZt13	BWZt21	1.00	0.00
8.2378470.2380599	8	2378470	2380599	2130	1708			yes		BWZt9	BWZt13	BWZt21	1.00	0.00
8.2403753.2409329	8	2403753	2409329	5577	5495			yes		BWZt9	BWZt13	BWZt21	1.00	0.00
8.2418353.2420254	8	2418353	2420254	1902	324			yes				BWZt21	1.00	0.03
8.2428790.2431187	8	2428790	2431187	2398	443			yes	BWZt4				1.00	0.03
8.2431279.2439751	8	2431279	2439751	8473	5831			yes		BWZt9	BWZt13	BWZt21	1.00	0.00
9.2.19666	9	2	19666	19665	32500			yes	BWZt4	BWZt9	BWZt13	BWZt21	0.97	0.00
9.64052.64887	9	64052	64887	836	8422			yes	BWZt4	BWZt9	BWZt13	BWZt21	1.00	0.00
9.66682.67503	9	66682	67503	822	6768			yes		BWZt9	BWZt13	BWZt21	1.00	0.00
9.166474.167593	9	166474	167593	1120	427	yes					BWZt13		1.00	0.02
9.170649.171598	9	170649	171598	950	1023	yes					BWZt13		1.00	0.02
9.282960.283771	9	282960	283771	812	7060			yes	BWZt4	BWZt9	BWZt13	BWZt21	1.00	0.00
9.293777.294104	9	293777	294104	328	2067						BWZt13	BWZt21	1.00	0.00
9.403302.404358	9	403302	404358	1057	6079			yes	BWZt4	BWZt9	BWZt13	BWZt21	1.00	0.00
9.406820.407637	9	406820	407637	818	5652			yes	BWZt4	BWZt9	BWZt13	BWZt21	1.00	0.00
9.972252.973061	9	972252	973061	810	6699			yes	BWZt4	BWZt9	BWZt13	BWZt21	1.00	0.00
9.1327176.1327455	9	1327176	1327455	280	1079	yes					BWZt13		1.00	0.00
9.1413289.1414068	9	1413289	1414068	780	5207			yes	BWZt4	BWZt9	BWZt13	BWZt21	1.00	0.00
9.1417709.1418662	9	1417709	1418662	954	7248			yes	BWZt4	BWZt9	BWZt13	BWZt21	1.00	0.00
9.1426458.1427268	9	1426458	1427268	811	8397			yes	BWZt4	BWZt9	BWZt13	BWZt21	1.00	0.00
9.1433239.1434246	9	1433239	1434246	1008	8398			yes	BWZt4	BWZt9	BWZt13	BWZt21	1.00	0.00
9.1755672.1756026	9	1755672	1756026	355	486					BWZt9			1.00	0.01
9.1792703.1793023	9	1792703	1793023	321	535					BWZt9			1.00	0.03
9.1904100.1904911	9	1904100	1904911	812	20600	yes			BWZt4	BWZt9			1.00	0.00
9.1963691.1964465	9	1963691	1964465	775	5928			yes	BWZt4	BWZt9	BWZt13	BWZt21	1.00	0.00
9.1998601.1999461	9	1998601	1999461	861	8548			yes	BWZt4	BWZt9	BWZt13	BWZt21	1.00	0.00
9.2127241.2137629	9	2127241	2137629	10389	8893			yes		BWZt9	BWZt13	BWZt21	1.00	0.00
10.57964.59013	10	57964	59013	1050	164							BWZt21	1.00	0.03
10.160001.161905	10	160001	161905	1905	284							BWZt21	1.00	0.03
10.229566.233698	10	229566	233698	4133	1942						BWZt13		1.00	0.00
10.445195.445392	10	445195	445392	198	651000		5S rRNA		BWZt4	BWZt9	BWZt13	BWZt21	1.00	0.00
10.531312.533737	10	531312	533737	2426	1011				BWZt4				1.00	0.00
10.797801.797951	10	797801	797951	151	7899				BWZt4				1.00	0.00
10.1059761.1061128	10	1059761	1061128	1368	308						BWZt13	BWZt21	1.00	0.02
10.1407605.1408991	10	1407605	1408991	1387	7568			yes	BWZt4	BWZt9	BWZt13	BWZt21	1.00	0.00
10.1638022.1644097	10	1638022	1644097	6076	5301			yes		BWZt9	BWZt13	BWZt21	1.00	0.00
11.32325.33623	11	32325	33623	1299	7625			yes	BWZt4	BWZt9	BWZt13	BWZt21	1.00	0.00
11.36845.38682	11	36845	38682	1838	6518			yes	BWZt4	BWZt9	BWZt13	BWZt21	1.00	0.00
11.760814.761682	11	760814	761682	869	6828			yes	BWZt4	BWZt9	BWZt13	BWZt21	1.00	0.00
11.768223.768327	11	768223	768327	105	1930					BWZt9	BWZt13	BWZt21	1.00	0.00
11.984387.985163	11	984387	985163	777	5718			yes	BWZt4	BWZt9	BWZt13	BWZt21	1.00	0.00
11.1119359.1119964	11	1119359	1119964	606	1513				BWZt4	BWZt9		BWZt21	1.00	0.00
11.1235102.1235419	11	1235102	1235419	318	1889				BWZt4	BWZt9	BWZt13	BWZt21	1.00	0.00
11.1593332.1594253	11	1593332	1594253	922	5471			yes	BWZt4	BWZt9	BWZt13	BWZt21	1.00	0.00
11.1597670.1598473	11	1597670	1598473	804	5719			yes	BWZt4	BWZt9	BWZt13	BWZt21	1.00	0.00
11.1615364.1617483	11	1615364	1617483	2120	672			yes		BWZt9	BWZt13	BWZt21	1.00	0.00
12.475.5013	12	475	5013	4539	2488			yes		BWZt9	BWZt13	BWZt21	1.00	0.00
12.6522.12487	12	6522	12487	5966	2621			yes		BWZt9	BWZt13	BWZt21	1.00	0.00
12.13820.18839	12	13820	18839	5020	2711			yes		BWZt9	BWZt13	BWZt21	1.00	0.00
12.106740.107675	12	106740	107675	936	6795			yes	BWZt4	BWZt9	BWZt13	BWZt21	1.00	0.00
12.462748.462907	12	462748	462907	160	7669					BWZt9	BWZt13	BWZt21	1.00	0.00
12.494199.496373	12	494199	496373	2175	629							BWZt21	1.00	0.04
12.607359.608250	12	607359	608250	892	8532			yes	BWZt4	BWZt9	BWZt13	BWZt21	1.00	0.00
12.681007.681905	12	681007	681905	899	7690			yes	BWZt4	BWZt9	BWZt13	BWZt21	1.00	0.00
12.732520.732860	12	732520	732860	341	7555				BWZt4	BWZt9	BWZt13	BWZt21	1.00	0.00
12.778427.778715	12	778427	778715	289	26900				BWZt4	BWZt9	BWZt13	BWZt21	1.00	0.00
12.832633.835493	12	832633	835493	2861	498							BWZt21	1.00	0.02
12.870648.871567	12	870648	871567	920	4231						BWZt13	BWZt21	1.00	0.00
12.1012309.1012492	12	1012309	1012492	184	4578					BWZt9	BWZt13	BWZt21	1.00	0.00
12.1121157.1124000	12	1121157	1124000	2844	1213						BWZt13	BWZt21	1.00	0.00
12.1402101.1405654	12	1402101	1405654	3554	303							BWZt21	1.00	0.02
12.1427223.1442420	12	1427223	1442420	15198	12800			yes		BWZt9	BWZt13	BWZt21	1.00	0.00
12.1442499.1445629	12	1442499	1445629	3131	2138			yes		BWZt9	BWZt13	BWZt21	1.00	0.00
13.3888.6772	13	3888	6772	2885	615			yes			BWZt13	BWZt21	1.00	0.01
13.6864.9270	13	6864	9270	2407	580			yes	BWZt4				1.00	0.03
13.12950.15733	13	12950	15733	2784	343							BWZt21	1.00	0.03
13.17800.20460	13	17800	20460	2661	581			yes			BWZt13	BWZt21	1.00	0.00
13.93164.93950	13	93164	93950	787	7338			yes	BWZt4	BWZt9	BWZt13	BWZt21	1.00	0.00
13.183698.187962	13	183698	187962	4265	18100			yes	BWZt4	BWZt9	BWZt13	BWZt21	1.00	0.00
13.320524.320760	13	320524	320760	237	1620			yes			BWZt13	BWZt21	1.00	0.03
13.442993.443802	13	442993	443802	810	2419			yes		BWZt9	BWZt13		1.00	0.01
13.479338.479478	13	479338	479478	141	651000		5S rRNA		BWZt4	BWZt9	BWZt13	BWZt21	1.00	0.00
13.645590.645871	13	645590	645871	282	651000		5S rRNA		BWZt4	BWZt9	BWZt13	BWZt21	1.00	0.00
13.775785.776744	13	775785	776744	960	7477			yes	BWZt4	BWZt9	BWZt13	BWZt21	1.00	0.00
13.846733.850190	13	846733	850190	3458	738						BWZt13	BWZt21	1.00	0.01
13.910888.911312	13	910888	911312	425	6533			yes	BWZt4	BWZt9	BWZt13	BWZt21	1.00	0.00
13.951679.951897	13	951679	951897	219	960					BWZt9	BWZt13	BWZt21	1.00	0.00
13.1030685.1031551	13	1030685	1031551	867	6198			yes	BWZt4	BWZt9	BWZt13	BWZt21	1.00	0.00
13.1163358.1164368	13	1163358	1164368	1011	7628			yes	BWZt4	BWZt9	BWZt13	BWZt21	1.00	0.00
13.1168422.1177165	13	1168422	1177165	8744	4812			yes		BWZt9	BWZt13	BWZt21	1.00	0.00
13.1177242.1180173	13	1177242	1180173	2932	556			yes		BWZt9	BWZt13	BWZt21	1.00	0.00
13.1180236.1184672	13	1180236	1184672	4437	3083			yes	BWZt4	BWZt9	BWZt13	BWZt21	1.00	0.00
14.1.5659	14	1	5659	5659	2757			yes		BWZt9	BWZt13		1.00	0.00
14.5736.29448	14	5736	29448	23713	32900			yes	BWZt4	BWZt9	BWZt13	BWZt21	1.00	0.00
14.32035.35785	14	32035	35785	3751	2495			yes		BWZt9	BWZt13	BWZt21	1.00	0.00
14.280035.280949	14	280035	280949	915	8400			yes	BWZt4	BWZt9	BWZt13	BWZt21	1.00	0.00
14.294633.295658	14	294633	295658	1026	6317			yes	BWZt4	BWZt9	BWZt13	BWZt21	1.00	0.00
14.532521.533209	14	532521	533209	689	6133			yes	BWZt4	BWZt9	BWZt13	BWZt21	1.00	0.00
14.546974.547880	14	546974	547880	907	5036			yes	BWZt4	BWZt9	BWZt13	BWZt21	1.00	0.00
14.632345.633222	14	632345	633222	878	7820			yes	BWZt4	BWZt9	BWZt13	BWZt21	1.00	0.00
14.644522.646581	14	644522	646581	2060	456							BWZt21	1.00	0.02
14.712306.713157	14	712306	713157	852	7576			yes	BWZt4	BWZt9	BWZt13	BWZt21	1.00	0.00
14.744382.745201	14	744382	745201	820	7678			yes	BWZt4	BWZt9	BWZt13	BWZt21	1.00	0.00
14.767975.769865	14	767975	769865	1891	348					BWZt9	BWZt13	BWZt21	1.00	0.05
14.769927.771285	14	769927	771285	1359	6122			yes		BWZt9	BWZt13	BWZt21	1.00	0.00
15.2.14750	15	2	14750	14749	19300			yes	BWZt4	BWZt9	BWZt13	BWZt21	1.00	0.00
15.14826.24490	15	14826	24490	9665	9097			yes	BWZt4	BWZt9	BWZt13	BWZt21	1.00	0.00
15.25780.26891	15	25780	26891	1112	3419			yes		BWZt9	BWZt13	BWZt21	1.00	0.00
15.44255.45101	15	44255	45101	847	7099			yes	BWZt4	BWZt9	BWZt13	BWZt21	1.00	0.00
15.51821.52932	15	51821	52932	1112	8984			yes	BWZt4	BWZt9	BWZt13	BWZt21	1.00	0.00
15.166892.167796	15	166892	167796	905	7609			yes	BWZt4	BWZt9	BWZt13	BWZt21	1.00	0.00
15.325288.325837	15	325288	325837	550	1175						BWZt13	BWZt21	1.00	0.00
15.583955.585999	15	583955	585999	2045	1528			yes		BWZt9	BWZt13	BWZt21	1.00	0.00
15.589581.597368	15	589581	597368	7788	5681			yes		BWZt9	BWZt13	BWZt21	1.00	0.00

15.597420.601006	15	597420	601006	3587	2454		yes		BWZt9	BWZt13	BWZt21	1.00	0.00
15.601708.639500	15	601708	639500	37793	44900		yes	BWZt4	BWZt9	BWZt13	BWZt21	0.97	0.00
16.2.25177	16	2	25177	25176	29200		yes	BWZt4	BWZt9	BWZt13	BWZt21	1.00	0.00
16.26979.31598	16	26979	31598	4620	1045				BWZt9	BWZt13	BWZt21	1.00	0.00
16.36395.40088	16	36395	40088	3694	1016		yes		BWZt9	BWZt13	BWZt21	1.00	0.00
16.84151.85006	16	84151	85006	856	6833		yes		BWZt9	BWZt13	BWZt21	1.00	0.00
16.89218.90204	16	89218	90204	987	7976		yes	BWZt4	BWZt9	BWZt13	BWZt21	1.00	0.00
16.412864.413679	16	412864	413679	816	8179		yes	BWZt4	BWZt9	BWZt13	BWZt21	1.00	0.00
16.420030.421422	16	420030	421422	1393	4996		yes	BWZt4	BWZt9	BWZt13	BWZt21	1.00	0.00
16.429283.430197	16	429283	430197	915	5759		yes		BWZt9	BWZt13	BWZt21	1.00	0.00
16.578572.583821	16	578572	583821	5250	4864		yes		BWZt9	BWZt13	BWZt21	1.00	0.00
16.597962.607044	16	597962	607044	9083	11000		yes	BWZt4	BWZt9	BWZt13	BWZt21	1.00	0.00
17.20145.23364	17	20145	23364	3220	12500		yes	BWZt4	BWZt9	BWZt13	BWZt21	1.00	0.00
17.23479.32114	17	23479	32114	8636	7584		yes		BWZt9	BWZt13	BWZt21	1.00	0.00
17.32194.70869	17	32194	70869	38676	34800	tRNA-Tyr	yes	BWZt4	BWZt9	BWZt13	BWZt21	1.00	0.00
17.79759.81052	17	79759	81052	1294	6345		yes	BWZt4	BWZt9	BWZt13	BWZt21	1.00	0.00
17.561480.566789	17	561480	566789	5310	4601		yes		BWZt9	BWZt13	BWZt21	1.00	0.00
17.566841.583652	17	566841	583652	16812	17100		yes	BWZt4	BWZt9	BWZt13	BWZt21	1.00	0.00
18.8453.9307	18	8453	9307	855	7409		yes	BWZt4	BWZt9	BWZt13	BWZt21	1.00	0.00
18.85060.85835	18	85060	85835	776	7423		yes	BWZt4	BWZt9	BWZt13	BWZt21	1.00	0.00
18.94463.94734	18	94463	94734	272	4169		yes	BWZt4	BWZt9	BWZt13	BWZt21	1.00	0.00
18.112235.113035	18	112235	113035	801	6393		yes	BWZt4	BWZt9	BWZt13	BWZt21	1.00	0.00
18.237222.238251	18	237222	238251	1030	8647		yes	BWZt4	BWZt9	BWZt13	BWZt21	1.00	0.00
18.287412.292428	18	287412	292428	5017	783		yes			BWZt13	BWZt21	1.00	0.01
18.431645.432912	18	431645	432912	1268	7542		yes	BWZt4	BWZt9	BWZt13	BWZt21	1.00	0.00
18.536203.544521	18	536203	544521	8319	5487		yes		BWZt9	BWZt13	BWZt21	1.00	0.00
18.544573.545584	18	544573	545584	1012	4816		yes		BWZt9	BWZt13	BWZt21	1.00	0.00
19.2210.3581	19	2210	3581	1372	645		yes		BWZt9	BWZt13	BWZt21	1.00	0.01
19.3645.8939	19	3645	8939	5295	9526		yes	BWZt4	BWZt9	BWZt13	BWZt21	1.00	0.00
19.104217.104960	19	104217	104960	744	7287		yes	BWZt4	BWZt9	BWZt13	BWZt21	1.00	0.00
19.527513.527919	19	527513	527919	407	6989		yes	BWZt4	BWZt9	BWZt13	BWZt21	1.00	0.00
20.1166.4761	20	1166	4761	3596	2395		yes		BWZt9	BWZt13	BWZt21	1.00	0.00
20.5781.9528	20	5781	9528	3748	1156		yes		BWZt9	BWZt13	BWZt21	1.00	0.00
20.230607.231363	20	230607	231363	757	6902		yes	BWZt4	BWZt9	BWZt13	BWZt21	1.00	0.00
20.297635.298058	20	297635	298058	424	620		yes				BWZt21	1.00	0.02
20.453037.466416	20	453037	466416	13380	13900		yes		BWZt9	BWZt13	BWZt21	1.00	0.00
20.467436.471636	20	467436	471636	4201	2520		yes		BWZt9	BWZt13	BWZt21	1.00	0.00
21.2.26958	21	2	26958	26957	32800		yes	BWZt4	BWZt9	BWZt13	BWZt21	0.99	0.00
21.27372.40407	21	27372	40407	13036	13000		yes		BWZt9	BWZt13	BWZt21	1.00	0.00
21.40468.43135	21	40468	43135	2668	2069		yes		BWZt9	BWZt13	BWZt21	1.00	0.00
21.43194.45416	21	43194	45416	2223	3362		yes		BWZt9	BWZt13	BWZt21	1.00	0.00
21.227542.233177	21	227542	233177	5636	5514		yes		BWZt9	BWZt13	BWZt21	1.00	0.00
21.349300.352594	21	349300	352594	3295	1062					BWZt13	BWZt21	1.00	0.00
21.400171.401658	21	400171	401658	1488	959		yes	BWZt4	BWZt9	BWZt13	BWZt21	1.00	0.00
21.401722.409211	21	401722	409211	7490	3487		yes		BWZt9	BWZt13	BWZt21	1.00	0.00

†The difference of the maximum posterior probability between the fungus containing samples (infected wheat and in vitro culture) and the fungus free samples (mock-inoculated healthy wheat)

‡Minimum sRNA loci differential expression false discovery rate (FDR) in at least one comparison between the infected wheat samples and the corresponding mock samples

Table S5. Chromosomal distribution of *Zymoseptoria tritici* IPO323 sRNA loci and their activity (inferred from the sRNAseq data).

chromosome	chromosome length (nt)	number of sRNA loci‡	total length of sRNA loci (nt)	total sRNA counts from all loci	% chromosome occupied by sRNA loci	counts/1kb genomic DNA	counts/1kb sRNA loci
1	6,088,797	45	152,153	218,325	2.5	36	1,435
2	3,860,111	33	63,921	163,311	1.7	42	2,555
3	3,505,381	26	64,904	93,428	1.9	27	1,439
4	2,880,011	17	30,210	75,864	1.0	26	2,511
5	2,861,803	23	77,336	132,706	2.7	46	1,716
6	2,674,951	16	52,120	97,057	1.9	36	1,862
7	2,665,280	23	39,548	124,074	1.5	47	3,137
8	2,443,572	33	54,915	160,215	2.2	66	2,918
9	2,142,475	21	44,564	152,016	2.1	71	3,411
10	1,682,575	8	18,496	24,477	1.1	15	1,323
11	1,624,292	10	9,658	43,883	0.6	27	4,544
12	1,462,624	17	49,909	99,351	3.4	68	1,991
13	1,185,774	17	39,889	69,581	3.4	59	1,744
14	773,098	13	44,520	94,038	5.8	122	2,112
15	639,501	11	80,153	111,246	12.5	174	1,388
16	607,044	10	52,790	80,868	8.7	133	1,532
17	584,099	5	35,272	48,130	6.0	82	1,365
18	573,698	9	19,350	52,669	3.4	92	2,722
19	549,847	4	7,818	24,447	1.4	44	3,127
20	472,105	6	26,106	27,493	5.5	58	1,053
21	409,213	8	62,793	62,253	15.3	152	991

Accessory chromosomes are shown in blue font.

‡34 siRNA loci that overlap with tRNA, rRNA, snRNA, and snoRNA genes (see Table S4) were excluded from the analyses presented in this table

Table S6. *Zymoseptoria tritici* IPO323 sRNA loci and mature sRNAs predicted to target wheat transcripts.

A. sRNA loci generating mature sRNAs, which have predicted mRNAs targets in wheat

1.168753-169514
1.2-24760
1.24832-33907
1.2630102-2631752
1.3107284-3109571
1.3404853-3405015
1.4420671-4420786
1.4519579-4520495
1.4859784-4862055
1.5208279-5209283
1.52723-55652
1.55724-59340
1.5574650-5575522
1.59399-69532
1.6022523-6024517
1.6024577-6027831
1.6042189-6044283
1.6044579-6052106
1.6052501-6055814
1.6060199-6062484
1.6062562-6077812
1.6077865-6084551
1.610884-611019
1.681033-681226
1.912518-913393
1.952268-954112
2.1061972-1062929
2.1295279-1297239
2.1333569-1334006
2.1741314-1742939
2.1895220-1896101
2.1903263-1903455
2.1909278-1910404
2.236665-237674
2.3187737-3188483
2.3189040-3189775
2.3276658-3277710
2.3411312-3412084
2.341988-345005
2.3632877-3633814
2.3801864-3816890
2.3816969-3821391
2.3839620-3844042
2.400134-400399
2.47729-49709
2.532319-532572
2.819512-820307
3.1178-7084
3.1848836-1849733
3.2252943-2253304
3.2375162-2376724
3.2736368-2736981
3.2779587-2780080
3.3241153-3242334
3.3403412-3404238
3.387886-388363
3.391037-392727
3.401660-407274
3.587789-590578
3.7151-18710
3.822679-823459
4.1251113-1252024
4.170472-171758
4.2228316-2228727
4.234947-235725
4.2644748-2645586
4.272845-274005
4.2866934-2868658
4.2869853-2880010
5.1045197-1045805
5.1092560-1092727
5.181519-182321
5.1875780-1875912
5.2318983-2319321
5.2686132-2687400
5.2806545-2846948
5.2850297-2861802
5.514262-514929
5.523622-524805
5.557661-560726
5.59060-61544
5.748438-748459
5.918436-919203
5.934505-936848
6.1267883-1270184
6.16617-39543
6.173879-174893
6.2022916-2027069
6.2108904-2109094
6.2414949-2415730

6.39611-43211
6.597638-598424
6.673455-674376
6.782298-789507
6.804863-807140
6.927640-928434
6.999529-999949
7.1263898-1264763
7.127872-128627
7.1351801-1352313
7.179855-180653
7.1804941-1806212
7.1814849-1815870
7.2536483-2537653
7.2613424-2626681
7.452408-453395
7.582594-583749
7.584106-585040
7.594685-595531
7.755129-758077
7.951736-951950
8.135097-135842
8.1402168-1402594
8.1402809-1403198
8.1496933-1498574
8.1778342-1779282
8.1779347-1780499
8.1816353-1818241
8.1826676-1828336
8.1839987-1840797
8.1964648-1966838
8.2000570-2000759
8.2011199-2012128
8.2142675-2143478
8.2202983-2203732
8.2292152-2298576
8.2378470-2380599
8.2403753-2409329
8.2431279-2439751
8.420489-421629
8.558236-559373
8.564251-565700
9.1413289-1414068
9.1417709-1418662
9.1426458-1427268
9.1433239-1434246
9.170649-171598
9.1904100-1904911
9.1963691-1964465
9.1998601-1999461
9.2-19666
9.2127241-2137629
9.282960-283771
9.403302-404358
9.406820-407637
9.64052-64887
9.66682-67503
9.972252-973061
10.1407605-1408991
10.1638022-1644097
10.445195-445392
10.797801-797951
11.1119359-1119964
11.1593332-1594253
11.1597670-1598473
11.1615364-1617483
11.32325-33623
11.36845-38682
11.760814-761682
11.768223-768327
11.984387-985163
12.1012309-1012492
12.106740-107675
12.1427223-1442420
12.1442499-1445629
12.607359-608250
12.6522-12487
12.681007-681905
13.1030685-1031551
13.1163358-1164368
13.1168422-1177165
13.1180236-1184672
13.183698-187962
13.479338-479478
13.645590-645871
13.775785-776744
13.910888-911312
13.93164-93950
13.951679-951897
14.1-5659
14.280035-280949
14.294633-295658
14.32035-35785
14.532521-533209

14.5736-29448
14.632345-633222
14.712306-713157
14.744382-745201
14.769927-771285
15.14826-24490
15.166892-167796
15.2-14750
15.25780-26891
15.325288-325837
15.44255-45101
15.51821-52932
15.583955-585999
15.589581-597368
15.597420-601006
15.601708-639500
16.2-25177
16.26979-31598
16.412864-413679
16.420030-421422
16.429283-430197
16.578572-583821
16.597962-607044
16.84151-85006
16.89218-90204
17.20145-23364
17.23479-32114
17.32194-70869
17.561480-566789
17.566841-583652
17.79759-81052
18.112235-113035
18.237222-238251
18.431645-432912
18.536203-544521
18.544573-545584
18.8453-9307
18.85060-85835
19.104217-104960
19.3645-8939
19.527513-527919
20.230607-231363
20.297635-298058
20.453037-466416
21.2-26958
21.227542-233177
21.27372-40407
21.401722-409211
21.40468-43135
21.43194-45416

B. Mature sRNAs predicted to target wheat mRNAs

Mature sRNA ID	sRNA sequence
ZtsRNA1	TTGGGGAATCCGTAGTGTTGT
ZtsRNA1.1	GGGGAATCCGTGGTGTTGTATG
ZtsRNA2	TGCACTGGTTGCTCGAACGCT
ZtsRNA3	TAACCATCTTTCGGGTCTGACT
ZtsRNA3.1	AACCATCTTTCGGGTCTGACT
ZtsRNA3.2	GTTAACCATCTTTCGGGTCTGACT
ZtsRNA4	TGGAGATCGCGAAGGAGGTTC
ZtsRNA4.1	TCCATGGAGATCGCGAAGGAG
ZtsRNA5.1	TGGCTCAGCTGCTGGCTCTGG
ZtsRNA5.2	TGGCTCGGCTGCTGGCTCAGC
ZtsRNA6.1	TAGGATTGCCTCTATAGCCTG
ZtsRNA6.2	TGGGATTGCCTCTATAGCCTG
ZtsRNA7.1	TATGTCTCGGGCTCGTCGATA
ZtsRNA7.2	TGTGTCTCGGGCTCGTCGATA
ZtsRNA8.1	TTCGCGCTTCCTCTTAACCAT
ZtsRNA8.2	TTCGCGCTTCCTCTTAACTAT
ZtsRNA9.1	TAGTCGTCTCGTCCCAGAACT
ZtsRNA9.2	TAGTCGTCTCGTCCTAGAACT
ZtsRNA10.1	TAGTATCGTCCTTCCGTATCG
ZtsRNA10.2	TAGTATCGTCCTTCCGTGTCG
ZtsRNA11.1	TATCTGTTTAACATCTTCATC
ZtsRNA11.2	TATCTGTTTAACATCTTTATC
ZtsRNA12	AACACCTGTACGTGGTGCCGGCTA
ZtsRNA13.1	CAGGGAATTTTTGGCATACCA
ZtsRNA13.2	AGGGAATTTTTGGCATACCAC
ZtsRNA14.1	CACCAACGATGTTCTCTTTTT
ZtsRNA14.2	TCACCAACGATGTTCTCTTTT
ZtsRNA15.1	TGACAGCATCGTCGACAATGG
ZtsRNA15.2	TTGACAGCATCGTCGACAATG
ZtsRNA16.1	AAATGATTTAATGAGCCTGACT
ZtsRNA16.2	AATGATTTAATGAGCCTGACT
ZtsRNA17.1	TCCGTCGGCCTCAATCACACC
ZtsRNA17.2	TTCCGTCGGCCTCAATCACAC
ZtsRNA18.1	TATTGATGCCGGCGAATCGAT
ZtsRNA18.2	TGATGCCGGCGAATCGATTGG
ZtsRNA19.1	TACTTCGCTGCACGATCTCCC
ZtsRNA19.2	TCGCTGCACGATCTCCCGGAG
ZtsRNA20.1	TCGGTCCACAAAGCTCCAGCC

ZtsRNA20.2	TGCATCGGTCCACAAAGCTCC
ZtsRNA21.1	GGGCTTATGGTGCAGTGGTAGC
ZtsRNA21.2	TTATGGTGCAGTGGTAGCATT
ZtsRNA22.1	CTTTGGATCGGAGTTGTGGGATGG
ZtsRNA22.2	TTTGGATCGGAGTTGTGGGATGGC
ZtsRNA23	AATACACGTGGCGGCGGCGGT
ZtsRNA24.1	CCATCTTTTTTTTTGTCTGATC
ZtsRNA24.2	CTTACCATCTTTTTTTTTGTCTGA
ZtsRNA25	AATCCGAATCCGACTCTGACGACG
ZtsRNA26	CAATTCGTGCACCTTCTTTTT
ZtsRNA27	CCCGCAAGATCAGTCCTCTCG
ZtsRNA28	CGATGAGTAGGACGTAAAGTAG
ZtsRNA29	CTTTCCACTCCGACGTCATGT
ZtsRNA30.1	TATGGAGCGTGCGATTCTTTT
ZtsRNA30.2	TTATGGAGCGTGCGATTCTTT
ZtsRNA30.3	TTATGGAGCGTGCGATTCTTTT
ZtsRNA31.1	TTACGACTTTTCTTCTTACCT
ZtsRNA31.2	TTTACGACTTTTCTTCTTACC
ZtsRNA32.1	TCGAATCCCATTCTGTTCACC
ZtsRNA32.2	TCGAATCCCATTCTGTTCACCA
ZtsRNA32.3	TTCGAATCCCATTCTGTTCACCA
ZtsRNA33.1	CTTCGTCGAACGACCATGTTTT
ZtsRNA33.2	CTTCGTCGAACGACCATGTTTTT
ZtsRNA33.3	TCTTCGTCGAACGACCATGTT
ZtsRNA34.1	CACATCAGATTCTGATTCTGGG
ZtsRNA34.2	CATCACATCAGATTCTGATTC
ZtsRNA34.3	TCATCACATCAGATTCTGATT
ZtsRNA35.1	TCCGGCTCCTCTGATTCGTCG
ZtsRNA35.2	TGTCCGGCTCCTCTGATTCGT
ZtsRNA36.1	CGGAATTTTTGATGCCCCCCA
ZtsRNA36.2	CGTGTTAGGCGGAATTTTTGATGC
ZtsRNA36.3	GCGGAATTTTTGATGCCCCCC
ZtsRNA37.1	TACCAATTCGAGCTGCTCTTT
ZtsRNA37.2	TGCTCTTTGATCTGCTGATAG
ZtsRNA37.3	TTCGAGCTGCTCTTTGATCTG
ZtsRNA38.1	ACTGCCTCTGGCTGACGCGAA
ZtsRNA38.2	CGCCAGTATTGCACTGCCTCTGGC
ZtsRNA38.3	TCGCCAGTATTGCACTGCCTCTGG
ZtsRNA39.1	CCTTTGGACTGCTCTACGGAGC
ZtsRNA39.2	CCTTTGGACTGCTCTACGGAGCC
ZtsRNA39.3	CTTTGGACTGCTCTACGGAGC
ZtsRNA39.4	TTTGGACTGCTCTACGGAGCC
ZtsRNA40.1	ATGGCACTTTCGAAGACTCGG
ZtsRNA40.2	ATGGCACTTTCGAAGACTCGGC
ZtsRNA40.3	ATGGCACTTTCGAAGACTCGGCG
ZtsRNA40.4	ATGGCACTTTCGAAGACTCGGCGA
ZtsRNA41.1	TGGGCGTTGGGCGTTGGTCAG
ZtsRNA41.2	TGGGCGTTGGTCAGGGTCACC
ZtsRNA41.3	TTGGGCGTTGGGCGTTGGTCA
ZtsRNA41.4	TTGGGCGTTGGTCAGGGTCAC
ZtsRNA42.1	AGAAGGGAAACCAAGACAGTCT
ZtsRNA42.2	GAAGGGAAACCAAGACAGTCT
ZtsRNA43.1	TCAGGCAAAGGCTGACGACGA
ZtsRNA43.2	TTCAGGCAAAGGCTGACGACG
ZtsRNA43.3	TTCAGGCAAAGGCTGACGACGA
ZtsRNA44.1	TATCGTCGTCAGCCTTTGCCT
ZtsRNA44.2	TATCGTCGTCAGCCTTTGCCTG
ZtsRNA45.1	CCGTCCGATCAGCCATGATAA
ZtsRNA45.2	CTTCCCGTCCGATCAGCCATG
ZtsRNA45.3	CTTCCCGTCCGATCAGCCATGA
ZtsRNA45.4	CTTCCCGTCCGATCAGCCATT
ZtsRNA45.5	TTCCCGTCCGATCAGCCATGA
ZtsRNA46.1	TAGGACTGCTATTTTCCTAGG
ZtsRNA46.2	TATTTTCCTAGGACTGCTATC
ZtsRNA46.3	TCTTCCTAGGACTGCTATCTT
ZtsRNA46.4	TGCTATTTTCCTAGGACTGCT
ZtsRNA46.5	TGCTATTTTCCTAGGACTGCTA
ZtsRNA47.1	TATCTTCACGGGCGATGGTCG
ZtsRNA47.2	TGGCGACGATATCTTCACGGG
ZtsRNA48.1	AACGACGACGACGACTCTGAA
ZtsRNA48.2	CAACGACGACGACGACTCTGAA
ZtsRNA49.1	TACCTCGCCGTCTCAAATATC
ZtsRNA49.2	TTTTGCTCTACCTCGCCGTCT
ZtsRNA50.1	ACACTAGTACTCAAGTTGGTG
ZtsRNA50.2	CTAGTACTCAAGTTGGTGACA
ZtsRNA51.1	AAGTTGGTGACAATTGGGGAAT
ZtsRNA51.2	AGTTGGTGACAATTGGGGAAT
ZtsRNA51.3	TCAAGTTGGTGACAATTGGGG
ZtsRNA51.4	TGACAATTGGGGAATCCGTAG
ZtsRNA51.5	TGGTGACAATTGGGGAATCCG
ZtsRNA52.1	TTTGAGACGGCGAGGTAGAGC
ZtsRNA52.2	TTTGAGACGGCGAGGTAGAAC
ZtsRNA52.3	TGAGACGGCGAGGTAGAGCAA
ZtsRNA53	CTTTGACCTCTTCGGAGGCGTGAA
ZtsRNA54	GCACCTTCACGCGGTGGCCGTCGT
ZtsRNA55	GCCCAATCTTCCTTGGATGTCTGA
ZtsRNA56	GGACCCTTGGCGCAGCGGTAGCGT

ZtsRNA57	GGGTAGTTGGTCTAGGGGTAT
ZtsRNA58	GTGAGTATTACTCTAGTTCCGTTA
ZtsRNA59	TAACCACTCGGCCACCTGTCC
ZtsRNA60	TAACGATTTCTTTTAAGCTCT
ZtsRNA61	TAACGCGGGCCGCACCTTCCG
ZtsRNA62	TAAGCCACATCTCCATTGTAG
ZtsRNA63	TAAGGGACGTAGACGAGGTAG
ZtsRNA64	TAATAGGATAGTATAGAAGGC
ZtsRNA65	TACACTACGTCGTCGAGCTCC
ZtsRNA66	TACACTGCGAACTGCTCTACC
ZtsRNA67	TACACTGGTCCTTTCTTGAAG
ZtsRNA68	TACATACGAGGTGTTCTGCTG
ZtsRNA69	TACCTCTTGGATTTGCTGTCT
ZtsRNA70	TACGGATCTTCCTTCTACGAG
ZtsRNA71	TACTCATCGGACGGAGCCTTT
ZtsRNA72	TACTTACTCGTCCTGCTCGTC
ZtsRNA73	TACTTCCGGACAAAACGCCCT
ZtsRNA74	TACTTTCAAACTCGATCAATT
ZtsRNA75	TAGAAGCGATAGAGGAGGACT
ZtsRNA76	TAGAAGGAATGTCCGTAGATG
ZtsRNA77	TAGAATCTTTGAAATTAGCTA
ZtsRNA78	TAGACGCGGAGCTAGAGCTCG
ZtsRNA79	TAGACTCTGCTGACTAGGCAC
ZtsRNA80	TAGAGCAAAACCGGTGGCTAG
ZtsRNA81	TAGAGGACGAGGAGGACGAGG
ZtsRNA82	TAGAGGACGATTTCGACGACG
ZtsRNA83	TAGAGGTAGAGCTAGTAGACA
ZtsRNA84	TAGCAGCTCTCCGAGATCTCC
ZtsRNA85	TAGCATTGGGCGAGAAATCCG
ZtsRNA86	TAGCCACCGGTTTTGCTCTAC
ZtsRNA87	TAGCCGGCCTGCCCTAGCATC
ZtsRNA88	TAGCGACCGGACACGGACCCT
ZtsRNA89	TAGCGCTCGAGGATCTAGATT
ZtsRNA90	TAGCGGCAAGGTAGGCTAGAA
ZtsRNA91	TAGCGTGCGTAGCTCTCTATT
ZtsRNA92	TAGCGTTCTCGGGAGGATCTG
ZtsRNA93	TAGCTCGAGCGGTAACAGACT
ZtsRNA94	TAGCTCGTCCTGTTTTCCTAG
ZtsRNA95	TAGGATGGGGTAATCGACCCG
ZtsRNA96	TAGGCAGGAGACATCGAGACA
ZtsRNA97	TAGGGACATGCGATTTCGATT
ZtsRNA98	TAGGGCATTGCGAGTGAACAG
ZtsRNA99	TAGGTAAGAAGAAAAGTCGTA
ZtsRNA100	TAGGTATCGAACTCGATAATT
ZtsRNA101	TAGGTCTCTAGAAGCACTTCG
ZtsRNA102	TAGGTCTGGCCCTCCTCTAGC
ZtsRNA103	TAGTATCCGTCGTCGAGCTCG
ZtsRNA104	TAGTCGACGAAGTGCTTCCCG
ZtsRNA105	TAGTCGCATCTGTCGTAATCG
ZtsRNA106	TAGTTAGGGAAGTTCTGCTCT
ZtsRNA107	TATCATGACTGCTGCGTTGCC
ZtsRNA108	TATCCACTGTATACAACACTC
ZtsRNA109	TATCGGATCTCGTGGAGTATG
ZtsRNA110	TATCGGTAGTCTCTCTTTTTG
ZtsRNA111	TATCGTCGATTCTACAAGCCG
ZtsRNA112	TATCGTCGCCAACACCCTCGA
ZtsRNA113	TATCTCTGCCGGAGCTGAATG
ZtsRNA114	TATCTGACTTCACTTGACGAA
ZtsRNA115	TATGCTCGACGAACCAGGTCT
ZtsRNA116	TATGCTCGGTCGACACCATTC
ZtsRNA117	TATGGCGTGTGACTCAAGCTC
ZtsRNA118	TCAAAGGCGTCGGAGCTGCTG
ZtsRNA119	TCAACCGATCGCTTCGTCTCC
ZtsRNA120	TCAACGAGCGTGGATACTTCA
ZtsRNA121	TCACTTCTGAACCTTGTGACG
ZtsRNA122	TCAGAGTCGTCGTCAGAGTCG
ZtsRNA123	TCAGATCAGGTGCAAAGGTAG
ZtsRNA124	TCCGACGTCCCTCTAGTGCTT
ZtsRNA125	TCCGGAGCGTGCTTCTTTACG
ZtsRNA126	TCCGGATTCGGCTTCGCTATC
ZtsRNA127	TCCTCCGTCTCGTCCGCCTCG
ZtsRNA128	TCCTGTAAGCACTGGAGGGACG
ZtsRNA129	TCGAAGGGATGCTCGTAGAAG
ZtsRNA130	TCGACCGGTCTATTTCCCTCT
ZtsRNA131	TCGACTATGTCACTGGCAGCG
ZtsRNA132	TCGATTCCGGGGTCGACCTCA
ZtsRNA133	TCGCATTCTCGCACCGTCGCA
ZtsRNA134	TCGCCCCGAGCGGCTCTGACC
ZtsRNA135	TCGCCCGTGAAGATATCGTCG
ZtsRNA136	TCGCTTCGGCTCATACTCCTA
ZtsRNA137	TCGGACCCTTTCCCTTTCGAG
ZtsRNA138	TCGGATTCGGATTCAGAGTCG
ZtsRNA139	TCGGCTAGAATTCCTCCTCTA
ZtsRNA140	TCGGTAGGACGTTAAAAGGCT
ZtsRNA141	TCGGTCGCGGTAGAGCTAGCG
ZtsRNA142	TCGTTACTTCGCCGACCTCTC
ZtsRNA143	TCTCATGTGTCCATGTTAGCT

ZtsRNA144	TCTGAACCTCCTTCGCGATCC
ZtsRNA145	TGAAGGGCCTTGTCGAATACA
ZtsRNA146	TGACCAACGACACGAGCTGCT
ZtsRNA147	TGACCAGGTCTAACTCCAACT
ZtsRNA148	TGACGAAGTCTCTAGAGAAGG
ZtsRNA149	TGAGACTTGTCGAGCGGGCGA
ZtsRNA150	TGAGTCTGCTCTTCTTGTTAC
ZtsRNA151	TGATTGTTCCAGAAGATGAGT
ZtsRNA152	TGCACGTGTACCATTAGCCTG
ZtsRNA153	TGCCCGAGCCGTCCTCTGTAG
ZtsRNA154	TGCCGCTGGGCGGATTTCTCG
ZtsRNA155	TGCGACTGCAAGAGCAATTCG
ZtsRNA156	TGCGCTGCGACGTAGGAGACG
ZtsRNA157	TGCGTGCGGCCTTGAGCTTCTGA
ZtsRNA158	TGCTGGAGCTTGCAAACGAGT
ZtsRNA159	TGGACTGCTTCTTTCTTCTTC
ZtsRNA160	TGGCGATGGCGACTGATCTGA
ZtsRNA161	TGGCGCACCGCTGAACTTCCC
ZtsRNA162	TGGGAGACGTGGTAGCCATCG
ZtsRNA163	TGGTCGAGGGTGTTGGCGACG
ZtsRNA164	TGGTGTTTGGCTTCATCTGCG
ZtsRNA165	TGGTTATTCGTCGTGCTGATG
ZtsRNA166	TGTCCGGTCTTGTTCCTTAGG
ZtsRNA167	TGTCCTCGTCGTCGTCAAATG
ZtsRNA168	TGTCGTTGGTCACCGTCCAGT
ZtsRNA169	TGTCTTGGTTTCCCTTCCGAG
ZtsRNA170	TGTTCGCAACGTAGAGGGCTG
ZtsRNA171	TGTTGATCGAGGGCTCATTCC
ZtsRNA172	TGTTGGCGGCGATATCTCTAC
ZtsRNA173	TGTTGTGGTGGATCAAAGGCG
ZtsRNA174	TGTTTGATCCGGACTGTCTTG
ZtsRNA175	TTAATATCTCCGAGAACGACA
ZtsRNA176	TTACCCCATCCTAGCCACCGG
ZtsRNA177	TTCCACCTCTTCATCATTCCG
ZtsRNA178	TTCCAGATCCAGGTCGTGAACATC
ZtsRNA179	TTCCGCGGCAGTACTATGTCT
ZtsRNA180	TTCCTCTGCCGTCTCTCCTCG
ZtsRNA181	TTCCTTTTCCGACTGTTCTCG
ZtsRNA182	TTCGCCACCCTCTGAACGGTG
ZtsRNA183	TTCGTGTGCTCGTATTCGTGA
ZtsRNA184	TTGCGGTCGACGAATCAGAGG
ZtsRNA185	TTGCTACGTCAGTCTGAGCTA
ZtsRNA186	TTGGATTGAGGTCTGGTGAAT
ZtsRNA187	TTGGCGACCACGATCTCCTCG
ZtsRNA188	TTGTTGCTCTGATCAATGCAC
ZtsRNA189	TTTCCAGCCCACCTTGCCGCT
ZtsRNA190	TTTCGAGAAGGATTTCGGGCG
ZtsRNA191	TTTCTGCTCTGCTAGCTTTCT
ZtsRNA192	TTTGCACCTGATCTGAACTGA
ZtsRNA193	TTTGCCGAGGACTCTCCTAAG

C. sRNA loci generating the four mature sRNAs (ZtsRNA1 - ZtsRNA4) selected for an in depth study

sRNA locus	Mature sRNA ID	length (nt)	sRNA read counts from all samples	overlaps with protein coding genes	overlaps with other genes	overlaps with TE / repetitive elements	sRNA loci active at 4, 9, 13, or 21 dpi during wheat infection				posterior probability difference†	Min FDR differential expression‡
1.3404853-3405015	ZtsRNA1	163	648000	-	-	-	BWZt4	BWZt9	BWZt13	BWZt21	1.00	0.00
2.532319-532572	ZtsRNA1	254	648000	-	-	-	BWZt4	BWZt9	BWZt13	BWZt21	1.00	0.00
2.1903263-1903455	ZtsRNA1	193	648000	-	-	-	BWZt4	BWZt9	BWZt13	BWZt21	1.00	0.00
3.3241153-3242334	ZtsRNA1	1182	651000	yes	-	-	BWZt4	BWZt9	BWZt13	BWZt21	1.00	0.00
5.514262-514929	ZtsRNA1	668	648000	yes	-	-	BWZt4	BWZt9	BWZt13	BWZt21	1.00	0.00
8.2000570-2000759	ZtsRNA1	190	648000	-	-	-	BWZt4	BWZt9	BWZt13	BWZt21	1.00	0.00
10.445195-445392	ZtsRNA1	198	651000	-	-	-	BWZt4	BWZt9	BWZt13	BWZt21	1.00	0.00
13.479338-479478	ZtsRNA1	141	651000	-	-	-	BWZt4	BWZt9	BWZt13	BWZt21	1.00	0.00
13.645590-645871	ZtsRNA1	282	651000	-	-	-	BWZt4	BWZt9	BWZt13	BWZt21	1.00	0.00
1.3107284-3109571	ZtsRNA2	2288	17700	yes	-	yes	BWZt4	BWZt9	BWZt13	BWZt21	1.00	0.00
2.341988-345005	ZtsRNA2	3018	17800	yes	-	yes	BWZt4	BWZt9	BWZt13	BWZt21	1.00	0.00
5.557661-560726	ZtsRNA2	3066	17900	yes	-	yes	BWZt4	BWZt9	BWZt13	BWZt21	1.00	0.00
13.183698-187962	ZtsRNA2	4265	18100	-	-	yes	BWZt4	BWZt9	BWZt13	BWZt21	1.00	0.00
3.2375162-2376724	ZtsRNA3	1563	3326	yes	-	-			BWZt13		1.00	0.00
8.1402809-1403198	ZtsRNA4	390	16800	-	-	-	BWZt4	BWZt9	BWZt13	BWZt21	1.00	0.00

Table S7. Wheat transcripts predicted to be targeted by *Zymoseptoria tritici* IPO323 mature sRNAs.

targeted wheat transcript	wheat transcript annotation
Traes_1AL_0F92F56F8.1	mitochondrial-processing peptidase subunit, mitochondrial precursor, putative, expressed;0.0;723
Traes_1AL_14F4067BA.1	cytochrome b6, putative, expressed;4e-31;133
Traes_1AL_1CB3A83EE.1	IQ calmodulin-binding motif family protein, putative, expressed;0.0;843
Traes_1AL_2B919E578.3	crcB-like protein, expressed;0.0;680
Traes_1AL_375367151.1	fatty acid hydroxylase, putative, expressed;4e-144;508
Traes_1AL_486E54C74.1	expressed protein;0.0;761
Traes_1AL_4A458DF14.1	PPR repeat containing protein, expressed;0.0;844
Traes_1AL_6CD69EE7E.1	cobalt ion transporter, putative, expressed;9e-150;527
Traes_1AL_99F5BB0D8.1	methyltransferase, putative, expressed;0.0;1089
Traes_1AL_A3B85CAE6.1	tesmin/TSO1-like CXC domain containing protein, expressed;1e-09;60.8
Traes_1AL_ABE93C8E7.1	peptidyl-tRNA hydrolase, mitochondrial precursor protein, putative, expressed;3e-97;352
Traes_1AL_B33A889C4.1	endonuclease/exonuclease/phosphatase family domain containing protein, expressed;0.0;783
Traes_1AL_C4650CF30.3	selT-like protein precursor, putative, expressed;6e-65;243
Traes_1AL_C4B40A214.1	NAD dependent epimerase/dehydratase family protein, putative, expressed;0.0;737
Traes_1AL_D1E192C9C.2	leucine-rich repeat family protein, putative, expressed;0.0;711
Traes_1AL_D74509506.2	ternary complex factor MIP1, putative, expressed;0.0;757
Traes_1AL_E7F4CC5A1.1	zinc finger, C3HC4 type domain containing protein, expressed;6e-121;431
Traes_1AL_EB5141D4C.7	NBS-LRR disease resistance protein, putative, expressed;0.0;667
Traes_1AS_AC509D7F5.1	protein kinase APK1A, chloroplast precursor, putative, expressed;5e-171;598
Traes_1AS_B3DDADFFA.1	AGC_PVPK_like_kin82y.12 - ACG kinases include homologs to PKA, PKG and PKC, expressed;0.0;653
Traes_1AS_D2C38EEF3.1	nuclear factor related to kappa-B-binding protein, related, putative, expressed;0.0;1539
Traes_1AS_EB5297361.1	white-brown complex homolog protein 12, putative, expressed;0.0;996
Traes_1BL_0EABC6DF5.1	expressed protein;0.0;929
Traes_1BL_190920E1E.1	glucose-1-phosphate adenylyltransferase large subunit, chloroplast precursor, putative, expressed;0.0;860
Traes_1BL_29B4E633D.1	IQ calmodulin-binding motif family protein, putative, expressed;0.0;829
Traes_1BL_39AE9ED1C.1	SAM dependent carboxyl methyltransferase, putative, expressed;1e-111;400
Traes_1BL_3EEA722DF.1	ZOS5-12 - C2H2 zinc finger protein, expressed;6e-36;149
Traes_1BL_42C7AFE7A.2	OsFBX53 - F-box domain containing protein, expressed;3e-110;396
Traes_1BL_44DACABDA.1	expressed protein;0.0;634
Traes_1BL_4B54A24F9.1	n/a
Traes_1BL_6ABEEF6DC.1	peptidyl-tRNA hydrolase, mitochondrial precursor protein, putative, expressed;8e-82;300
Traes_1BL_7175F3833.1	zinc finger, C3HC4 type domain containing protein, expressed;2e-129;460
Traes_1BL_73EBB04B5.2	glycosyl hydrolase, family 31, putative, expressed;0.0;795
Traes_1BL_84EEAC5D5.4	mitochondrial-processing peptidase subunit, mitochondrial precursor, putative, expressed;4e-133;472
Traes_1BL_9046B5730.2	cytoplasmic membrane protein, putative, expressed;5e-33;137
Traes_1BL_93285025D.1	lipoxygenase, putative, expressed;2e-85;315
Traes_1BL_A0644DA53.1	ternary complex factor MIP1, putative, expressed;4e-128;455
Traes_1BL_B722F2F54.1	Leucine Rich Repeat family protein, expressed;1e-177;620
Traes_1BL_BEB9B5EF3.1	myosin-2 heavy chain, non muscle, putative, expressed;0.0;749
Traes_1BL_C943EF0D8.2	selT-like protein precursor, putative, expressed;3e-65;244
Traes_1BL_DF929E48F.1	crcB-like protein, expressed;0.0;681
Traes_1BL_E9106179C.2	AGC_PVPK_like_kin82y.12 - ACG kinases include homologs to PKA, PKG and PKC, expressed;8e-63;239
Traes_1BL_EABF29D66.1	OsSub7 - Putative Subtilisin homologue, expressed;2e-67;252
Traes_1BL_EC9A57F9E.1	endonuclease/exonuclease/phosphatase family domain containing protein, expressed;0.0;780
Traes_1BL_FDCE71D9E.1	zinc finger, C3HC4 type domain containing protein, expressed;2e-73;274
Traes_1BS_307AF1E47.1	peroxidase precursor, putative, expressed;4e-64;242
Traes_1BS_3D04F8758.1	receptor-like protein kinase 2 precursor, putative, expressed;0.0;1704
Traes_1BS_4A63D1DBC.2	phosphatidate cytidylyltransferase, putative, expressed;0.0;700
Traes_1BS_6799B4072.1	cytochrome b6, putative, expressed;2e-25;114
Traes_1BS_77D2B4C0D.2	haloacid dehalogenase-like hydrolase family protein, putative, expressed;3e-165;578
Traes_1BS_ACC0652C7.1	transmembrane amino acid transporter protein, putative, expressed;0.0;785
Traes_1BS_B64557385.1	disease resistance protein RGA1, putative, expressed;0.0;691
Traes_1BS_B734EDEA7.1	metal cation transporter, putative, expressed;7e-132;468
Traes_1BS_DB050544B.1	KIP1, putative, expressed;9e-180;629
Traes_1BS_FEE01077D.1	expressed protein;3e-21;99.0
Traes_1DL_14F4067BA.1	cytochrome b6, putative, expressed;4e-31;133
Traes_1DL_1F7D352CA.1	NAD dependent epimerase/dehydratase family protein, putative, expressed;0.0;737
Traes_1DL_23506ECCE.2	IQ calmodulin-binding motif family protein, putative, expressed;0.0;843
Traes_1DL_4518A1BF6.1	peptidyl-tRNA hydrolase, mitochondrial precursor protein, putative, expressed;3e-86;315
Traes_1DL_4CFBFAC6C.1	flavonol synthase/flavanone 3-hydroxylase, putative, expressed;2e-53;206
Traes_1DL_560607990.1	DUF630/DUF632 domains containing protein, putative, expressed;0.0;744

Traes_1DL_580C04A3D.1	transporter family protein, putative, expressed;2e-142;502
Traes_1DL_5AD65B0D4.1	crcB-like protein, expressed;0.0;680
Traes_1DL_668C082D7.1	Leucine Rich Repeat family protein, expressed;0.0;1344
Traes_1DL_7DDE52966.1	tesmin/TSO1-like CXC domain containing protein, expressed;5e-08;55.8
Traes_1DL_808AF52AC.2	OsFBX53 - F-box domain containing protein, expressed;4e-110;396
Traes_1DL_81BFD05931.4	expressed protein;0.0;921
Traes_1DL_8EC90B950.1	selT-like protein precursor, putative, expressed;9e-67;249
Traes_1DL_91D91B69A.1	mitochondrial-processing peptidase subunit, mitochondrial precursor, putative, expressed;0.0;728
Traes_1DL_97C3D29AF.3	NBS-LRR disease resistance protein, putative, expressed;0.0;749
Traes_1DL_C06754AD1.1	zinc finger, C3HC4 type domain containing protein, expressed;4e-174;608
Traes_1DL_D2F45A44E.1	zinc finger, C3HC4 type domain containing protein, expressed;2e-130;462
Traes_1DL_ED171DA70.2	endonuclease/exonuclease/phosphatase family domain containing protein, expressed;0.0;778
Traes_1DL_F4B23A668.1	ternary complex factor MIP1, putative, expressed;0.0;703
Traes_1DS_14F4067BA.1	cytochrome b6, putative, expressed;4e-31;133
Traes_1DS_1571BA3C8.1	haloacid dehalogenase-like hydrolase family protein, putative, expressed;3e-165;578
Traes_1DS_475F92381.7	AGC_PVPK_like_kin82y.12 - ACG kinases include homologs to PKA, PKG and PKC, expressed;0.0;641
Traes_1DS_550739F69.2	expressed protein;4e-44;176
Traes_1DS_77D05F27F.1	phosphatidate cytidylyltransferase, putative, expressed;3e-121;432
Traes_1DS_791028249.1	expressed protein;5e-59;224
Traes_1DS_A6A528A3B.1	expressed protein;3e-56;216
Traes_1DS_D49FED82C.1	expressed protein;9e-101;363
Traes_2AL_091681562.2	pentatricopeptide, putative, expressed;0.0;1063
Traes_2AL_09879131A.1	plastid-specific 30S ribosomal protein 3, chloroplast precursor, putative, expressed;9e-50;194
Traes_2AL_0B9E1F386.1	WD domain, G-beta repeat domain containing protein, expressed;0.0;1563
Traes_2AL_1A638BE5E.1	L-ascorbate oxidase homolog precursor, putative, expressed;0.0;609
Traes_2AL_295E0C7C5.1	RNA-binding region RNP-1, putative, expressed;1e-111;402
Traes_2AL_2ABB28A56.1	RNA recognition motif containing protein, putative, expressed;0.0;714
Traes_2AL_2B32DB242.1	AIR9 protein, putative, expressed;0.0;727
Traes_2AL_2F1EBA9A5.1	RNA recognition motif containing protein, putative, expressed;5e-81;299
Traes_2AL_3035352F3.1	ZOS7-01 - C2H2 zinc finger protein, expressed;2e-29;127
Traes_2AL_34786C78C.1	inactive receptor kinase At1g27190 precursor, putative, expressed;1e-76;283
Traes_2AL_3E18AC376.1	expressed protein;1e-40;163
Traes_2AL_3E18AC3761.1	expressed protein;1e-40;163
Traes_2AL_3E1961FE8.1	cytochrome P450, putative, expressed;0.0;729
Traes_2AL_42E1AD58D.2	PPR repeat domain containing protein, putative, expressed;5e-118;423
Traes_2AL_57BF37A86.1	no apical meristem protein, putative, expressed;2e-119;427
Traes_2AL_5B6D378D4.1	expressed protein;2e-119;427
Traes_2AL_78B25BEC4.1	phosphatidylinositol-4-phosphate 5-kinase, putative, expressed;0.0;961
Traes_2AL_78EFCE67B.1	cytochrome P450, putative, expressed;4e-43;171
Traes_2AL_84DE2C67B.2	RNA recognition motif containing protein, putative, expressed;0.0;714
Traes_2AL_895F81BEB.2	potassium transporter, putative, expressed;7e-148;521
Traes_2AL_8F6A456B3.1	cytochrome P450, putative, expressed;0.0;782
Traes_2AL_90DC433FE.1	expressed protein;2e-65;246
Traes_2AL_A377FF6FE.1	TKL_IRAK_CR4L.3 - The CR4L subfamily has homology with Crinkly4, expressed;0.0;798
Traes_2AL_AA82F1ACF.2	calcium-transporting ATPase, plasma membrane-type, putative, expressed;3e-125;446
Traes_2AL_B5DE57D18.1	MYB family transcription factor, putative, expressed;2e-19;91.7
Traes_2AL_B7F30A338.1	histone H1, putative, expressed;4e-26;115
Traes_2AL_BFB0C6D4C.1	homeobox associated leucine zipper, putative, expressed;3e-50;195
Traes_2AL_CCE1B7CA8.2	transposon protein, putative, Mutator sub-class, expressed;8e-150;528
Traes_2AL_DFB829624.2	peptide transporter PTR2, putative, expressed;0.0;670
Traes_2AL_F24D031AA.1	AP2 domain containing protein, expressed;5e-69;258
Traes_2AL_F5D77DFC3.1	cytochrome b6, putative, expressed;1e-07;52.8
Traes_2AS_06B305ADA.1	zeta-carotene desaturase, chloroplast/chromoplast precursor, putative, expressed;0.0;1011
Traes_2AS_1437F6A1B.1	amidase, putative, expressed;0.0;668
Traes_2AS_14F4067BA.1	cytochrome b6, putative, expressed;4e-31;133
Traes_2AS_205BC3596.1	expressed protein;1e-63;241
Traes_2AS_20F68A948.1	expressed protein;6e-137;485
Traes_2AS_3B248893C.2	expressed protein;8e-100;360
Traes_2AS_42E368404.1	IQ calmodulin-binding motif family protein, expressed;0.0;639
Traes_2AS_447395A66.1	expressed protein;7e-26;113
Traes_2AS_4DE6446BD.2	WD domain, G-beta repeat domain containing protein, expressed;0.0;1478
Traes_2AS_53250F380.1	NADH-ubiquinone oxidoreductase 51 kDa subunit, mitochondrial precursor, putative, expressed;0.0;915
Traes_2AS_5CAE6A22E.1	FACT complex subunit SPT16, putative, expressed;0.0;1018
Traes_2AS_5F11D7ACF.1	phospholipase C, putative, expressed;8e-125;444

Traes_2AS_7C18F8EF6.1	1-phosphatidylinositol-4-phosphate 5-kinase/ zinc ion binding protein, putative, expressed;0.0;2303
Traes_2AS_92AA478DA.1	OsFBX275 - F-box domain containing protein, expressed;1e-97;354
Traes_2AS_B18B6B26F.1	BTBN16 - Bric-a-Brac, Tramtrack, Broad Complex BTB domain with non-phototropic hypocotyl 3 NPH3 domain, expre
Traes_2AS_B96BB2490.1	zinc finger C-x8-C-x5-C-x3-H type family protein, expressed;0.0;1036
Traes_2AS_BE8D336DB.1	ribosome recycling factor, putative, expressed;6e-93;338
Traes_2AS_BFBFCCAE2.1	cytochrome P450, putative, expressed;5e-28;120
Traes_2AS_DE63DCE1F.2	RNA recognition motif containing protein, putative, expressed;0.0;875
Traes_2AS_E17243D52.1	GRAS family transcription factor domain containing protein, expressed;5e-30;128
Traes_2AS_E7FED0F00.1	pentatricopeptide repeat domain containing protein, putative, expressed;0.0;1051
Traes_2AS_EC975D5AB.2	alpha-N-arabinofuranosidase A, putative, expressed;0.0;993
Traes_2AS_FA7059723.1	MYB family transcription factor, putative, expressed;3e-115;412
Traes_2BL_0895B944F.2	OTU-like cysteine protease family protein, putative, expressed;1e-126;451
Traes_2BL_08FC042C0.2	RNA-binding region RNP-1, putative, expressed;3e-109;394
Traes_2BL_10C9610DA.1	cytochrome P450, putative, expressed;0.0;694
Traes_2BL_1C31451BE.1	BTBZ4 - Bric-a-Brac, Tramtrack, Broad Complex BTB domain with TAZ zinc finger and Calmodulin-binding domains, ex
Traes_2BL_3CCE6F0A1.1	polygalacturonase inhibitor 1 precursor, putative, expressed;1e-45;181
Traes_2BL_428BF7743.1	cytochrome P450, putative, expressed;0.0;712
Traes_2BL_4C1D54940.1	potassium transporter, putative, expressed;0.0;1205
Traes_2BL_52480A403.2	oxidoreductase, short chain dehydrogenase/reductase family, putative, expressed;2e-104;377
Traes_2BL_5945B9FDB.1	histone H1, putative, expressed;8e-26;114
Traes_2BL_5DC21F3CD.1	AP2 domain containing protein, expressed;3e-69;259
Traes_2BL_6AEE8AC28.1	no apical meristem protein, putative, expressed;8e-121;431
Traes_2BL_6C9860B0D.1	expressed protein;1e-34;143
Traes_2BL_6F5788E6F.1	expressed protein;4e-72;268
Traes_2BL_754076A48.1	inactive receptor kinase At1g27190 precursor, putative, expressed;4e-81;298
Traes_2BL_78139C29C.2	expressed protein;4e-123;439
Traes_2BL_7A01C0963.4	wall-associated receptor kinase-like 20 precursor, putative, expressed;9e-116;414
Traes_2BL_8D03AD41F.1	cytochrome P450, putative, expressed;1e-116;417
Traes_2BL_9DD547641.1	WD domain, G-beta repeat domain containing protein, expressed;3e-120;429
Traes_2BL_9E38243B0.1	ZOS7-01 - C2H2 zinc finger protein, expressed;3e-33;139
Traes_2BL_9E38243B01.1	ZOS7-01 - C2H2 zinc finger protein, expressed;3e-33;139
Traes_2BL_AC1A3ADBE.2	cadmium tolerance factor, putative, expressed;4e-167;587
Traes_2BL_B15F55EBC.2	RNA recognition motif containing protein, putative, expressed;0.0;711
Traes_2BL_B4193BAEF.1	peptide transporter PTR2, putative, expressed;0.0;912
Traes_2BL_B57DA0C02.2	L-ascorbate oxidase homolog precursor, putative, expressed;0.0;727
Traes_2BL_B69300543.1	homeobox associated leucine zipper, putative, expressed;6e-66;248
Traes_2BL_B9145D951.2	potassium channel AKT1, putative, expressed;0.0;1174
Traes_2BL_C3676B9CC.1	POEI19 - Pollen Ole e I allergen and extensin family protein precursor, expressed;5e-57;219
Traes_2BL_C84D00D9B.1	protein kinase, putative, expressed;2e-47;184
Traes_2BL_C9E82112E1.1	aspartic proteinase nepenthesin precursor, putative, expressed;3e-148;523
Traes_2BL_D48F61FF3.1	U-box domain-containing protein, putative, expressed;0.0;665
Traes_2BL_DADDEF6D8.2	PPR repeat domain containing protein, putative, expressed;0.0;815
Traes_2BL_E2DCFA60B.1	calcium-transporting ATPase, plasma membrane-type, putative, expressed;0.0;764
Traes_2BL_E54974B70.1	pentatricopeptide repeat domain containing protein, putative, expressed;1e-163;573
Traes_2BL_E7871AFBA.1	AP2 domain containing protein, expressed;3e-69;259
Traes_2BL_EC380C112.1	TKL_IRAK_CR4L.3 - The CR4L subfamily has homology with Crinkly4, expressed;0.0;697
Traes_2BL_EF68D6200.1	AIR9 protein, putative, expressed;0.0;722
Traes_2BL_F304D7728.1	cytochrome P450, putative, expressed;5e-159;558
Traes_2BL_FA3B8F0E5.1	harpin-induced protein 1 domain containing protein, expressed;4e-71;265
Traes_2BL_FB11AE0BD.1	polygalacturonase inhibitor 1 precursor, putative, expressed;1e-45;181
Traes_2BL_FB11AE0BD1.1	polygalacturonase inhibitor 1 precursor, putative, expressed;1e-45;181
Traes_2BS_0AE7A9CC21.1	expressed protein;3e-138;489
Traes_2BS_0E78C009A.1	expressed protein;1e-65;247
Traes_2BS_23097C174.1	zinc finger C-x8-C-x5-C-x3-H type family protein, expressed;0.0;1182
Traes_2BS_2410051E8.1	histone acetyltransferase HAC5, putative, expressed;0.0;1539
Traes_2BS_2E4E870EF.1	ribosome recycling factor, putative, expressed;2e-92;336
Traes_2BS_58E7D5315.1	NAD dependent epimerase/dehydratase family domain containing protein, expressed;0.0;651
Traes_2BS_5D4F1CEF7.2	RNA recognition motif containing protein, putative, expressed;0.0;868
Traes_2BS_6374BF6AC.1	IQ calmodulin-binding motif family protein, expressed;0.0;661
Traes_2BS_662E31835.2	expressed protein;5e-107;385
Traes_2BS_72DCCC2AA.1	cytochrome P450 72A1, putative, expressed;0.0;811
Traes_2BS_91EC0D1B7.1	protein kinase, putative, expressed;0.0;913
Traes_2BS_95E3ABC9C.1	expressed protein;7e-147;518
Traes_2BS_9C52B176E.1	glucan endo-1,3-beta-glucosidase precursor, putative, expressed;1e-170;597

Traes_2BS_9CB52FFC4.1	zeta-carotene desaturase, chloroplast/chromoplast precursor, putative, expressed;0.0;1045
Traes_2BS_9D58086CF.1	pectinesterase, putative, expressed;0.0;796
Traes_2BS_A2C6C9BD6.1	stripe rust resistance protein Yr10, putative, expressed;4e-58;221
Traes_2BS_A4DCE06CE.2	WD domain, G-beta repeat domain containing protein, expressed;0.0;1607
Traes_2BS_A594778DC.1	expressed protein;3e-62;236
Traes_2BS_B713C8E76.1	stripe rust resistance protein Yr10, putative, expressed;6e-12;67.0
Traes_2BS_CAEE18AAC.1	DUF1645 domain containing protein, putative, expressed;4e-63;239
Traes_2BS_D580B56A3.1	expressed protein;3e-161;566
Traes_2BS_E35529023.1	NADH-ubiquinone oxidoreductase 51 kDa subunit, mitochondrial precursor, putative, expressed;0.0;916
Traes_2BS_E5732CD2C.1	alpha-N-arabinofuranosidase A, putative, expressed;0.0;991
Traes_2BS_E6A403F63.1	cytochrome P450 72A1, putative, expressed;0.0;822
Traes_2BS_F75822422.1	expressed protein;5e-167;585
Traes_2BS_FDABA21A7.1	GRAS family transcription factor domain containing protein, expressed;0.0;720
Traes_2DL_037522CF1.1	RNA recognition motif containing protein, putative, expressed;0.0;717
Traes_2DL_09A84CB09.1	expressed protein;3e-70;262
Traes_2DL_10C74B50E.2	RNA-binding region RNP-1, putative, expressed;4e-111;401
Traes_2DL_3EBEC47B3.1	polygalacturonase inhibitor 1 precursor, putative, expressed;2e-48;107
Traes_2DL_6E913E1A4.1	PsbP, putative, expressed;5e-79;290
Traes_2DL_75BF1DBF2.1	pentatricopeptide, putative, expressed;1e-91;333
Traes_2DL_8B3712638.1	expressed protein;9e-14;75.5
Traes_2DL_8B66F30F9.1	ent-kaurene synthase, chloroplast precursor, putative, expressed;0.0;889
Traes_2DL_9833B9F07.1	expressed protein;3e-06;48.9
Traes_2DL_9FDA5C607.1	cytochrome P450, putative, expressed;3e-72;268
Traes_2DL_AA291009C.1	AP2 domain containing protein, expressed;5e-07;50.4
Traes_2DL_AB6FEEC91.1	histone H1, putative, expressed;2e-24;110
Traes_2DL_B250957E6.1	NB-ARC/LRR disease resistance protein, putative, expressed;3e-54;207
Traes_2DL_C4B1C27CA.1	RNA recognition motif containing protein, putative, expressed;1e-48;190
Traes_2DL_CF9C6D943.2	protein kinase, putative, expressed;3e-60;228
Traes_2DL_DF7B995C9.2	no apical meristem protein, putative, expressed;1e-79;275
Traes_2DL_EF06B33F9.2	n/a
Traes_2DL_FAE13169F.1	calcium-transporting ATPase, plasma membrane-type, putative, expressed;0.0;1731
Traes_2DL_FE7C9635F.1	nuclease PA3, putative, expressed;3e-13;71.2
Traes_2DL_FE8D235D5.1	ZOS7-01 - C2H2 zinc finger protein, expressed;1e-32;137
Traes_2DS_0028CD306.1	zeta-carotene desaturase, chloroplast/chromoplast precursor, putative, expressed;0.0;1030
Traes_2DS_1125D8A30.1	cytochrome P450 72A1, putative, expressed;0.0;812
Traes_2DS_18DAEBA49.1	GTPase-activating protein, putative, expressed;1e-139;494
Traes_2DS_1AB8B3758.1	IQ calmodulin-binding motif family protein, expressed;0.0;652
Traes_2DS_1C28A840C.2	amidase, putative, expressed;3e-43;171
Traes_2DS_1E14A1C3A.2	Phosphatidylinositol kinase and FAT containing domain protein, putative, expressed;0.0;2120
Traes_2DS_29ED1D7E0.1	protein kinase, putative, expressed;0.0;922
Traes_2DS_2EC803D72.1	DUF1645 domain containing protein, putative, expressed;4e-08;54.3
Traes_2DS_2FAFF7235.1	transposon protein, putative, unclassified, expressed;3e-43;174
Traes_2DS_398F9E2F9.1	RNA recognition motif containing protein, putative, expressed;3e-19;91.3
Traes_2DS_3A03E6866.1	lectin-like receptor kinase, putative, expressed;3e-116;416
Traes_2DS_49959692B.1	MYB family transcription factor, putative, expressed;2e-102;369
Traes_2DS_49959692B1.1	MYB family transcription factor, putative, expressed;2e-102;369
Traes_2DS_5C8A4D416.1	dimethyladenosine transferase, putative, expressed;1e-136;483
Traes_2DS_64F37A63E.1	cytochrome P450, putative, expressed;0.0;647
Traes_2DS_795D042B8.1	flavin-containing monooxygenase family protein, putative, expressed;0.0;677
Traes_2DS_867D0797A.2	ribosome recycling factor, putative, expressed;1e-36;148
Traes_2DS_93608B31A.1	cytochrome b6, putative, expressed;2e-30;131
Traes_2DS_9B86CE58D.1	alpha-N-arabinofuranosidase A, putative, expressed;0.0;966
Traes_2DS_C911681D1.1	NADH-ubiquinone oxidoreductase 51 kDa subunit, mitochondrial precursor, putative, expressed;0.0;804
Traes_2DS_DE9475523.2	n/a
Traes_2DS_E5E62E1DF.1	expressed protein;1e-140;497
Traes_2DS_F9104B72E.1	FACT complex subunit SPT16, putative, expressed;0.0;1245
Traes_3AL_0D611A4EF.2	SET domain containing protein, expressed;2e-100;363
Traes_3AL_14F4067BA.1	cytochrome b6, putative, expressed;4e-31;133
Traes_3AL_20404D7E4.1	mitochondrial-processing peptidase subunit, mitochondrial precursor, putative, expressed;9e-163;570
Traes_3AL_2975257C3.1	expressed protein;0.0;952
Traes_3AL_30C211BB7.1	expressed protein;0.0;764
Traes_3AL_32C9EEC67.1	antigen peptide transporter-like 1, chloroplast precursor, putative, expressed;2e-149;527
Traes_3AL_37EA7312B.2	pentatricopeptide repeat protein PPR1106-17, putative, expressed;0.0;672
Traes_3AL_3CE8B4063.1	transcriptional corepressor LEUNIG, putative, expressed;0.0;784

Traes_3AL_8C6F4F66A.1	GHMP kinases ATP-binding protein, putative, expressed;1e-127;454
Traes_3AL_9005F85AF.1	expressed protein;5e-170;596
Traes_3AL_925AAD7CE.1	ATP binding protein, putative, expressed;1e-131;467
Traes_3AL_A066DC175.1	beta-galactosidase precursor, putative, expressed;4e-32;134
Traes_3AL_AF9D811C3.1	expressed protein;2e-107;387
Traes_3AL_CA6B6A8CE.1	SET domain protein SDG117, putative, expressed;9e-12;67.0
Traes_3AL_D3D600952.1	S-locus-like receptor protein kinase, putative, expressed;0.0;1279
Traes_3AL_DDD7627D7.1	WD domain, G-beta repeat domain containing protein, expressed;1e-75;280
Traes_3AL_E4A0B69C5.2	galactosyltransferase, putative, expressed;4e-83;304
Traes_3AL_EB28DB33C.2	lipase, putative, expressed;0.0;897
Traes_3AL_FF2267EF5.2	zinc finger family protein, putative, expressed;0.0;825
Traes_3AS_0D233DB25.1	glucosidase II beta subunit-like domain containing protein, expressed;3e-152;535
Traes_3AS_42E490701.1	monocopper oxidase, putative, expressed;3e-79;293
Traes_3AS_43D5892CB.2	retrotransposon protein, putative, Ty1-copia subclass, expressed;0.0;1617
Traes_3AS_49AE2778F.1	expressed protein;3e-14;74.3
Traes_3AS_4E21250EA.2	wax synthase, putative, expressed;2e-134;477
Traes_3AS_5B1F212B0.1	transposon protein, putative, unclassified, expressed;5e-44;173
Traes_3AS_6678A7D6F.1	cytochrome P450, putative, expressed;2e-88;322
Traes_3AS_6706B4CCE.1	cytochrome b6, putative, expressed;1e-25;113
Traes_3AS_6AC58CA11.2	ternary complex factor MIP1, putative, expressed;1e-55;214
Traes_3AS_8D2099B24.1	nodal modulator 1 precursor, putative, expressed;2e-68;255
Traes_3AS_9CB9425D81.11	pentatricopeptide, putative, expressed;0.0;1827
Traes_3AS_A9D946CDB.1	importin-alpha re-exporter, putative, expressed;0.0;1224
Traes_3AS_ACBC778FF.2	XIK, putative, expressed;0.0;903
Traes_3AS_ACC13CC21.1	transposon protein, putative, unclassified, expressed;0.0;812
Traes_3AS_B6616C762.1	expressed protein;1e-65;246
Traes_3AS_C11206884.1	expressed protein;7e-152;535
Traes_3AS_EDDA9E245.1	cytokinin dehydrogenase precursor, putative, expressed;7e-28;119
Traes_3AS_F0C76D06E.1	retrotransposon protein, putative, unclassified, expressed;6e-09;57.0
Traes_3AS_F0C76D06E1.1	retrotransposon protein, putative, unclassified, expressed;6e-09;57.0
Traes_3B_00AC7406E.2	GDSL-like lipase/acylhydrolase, putative, expressed;4e-77;286
Traes_3B_017090820.1	AP2/EREBP transcription factor BABY BOOM, putative, expressed;7e-127;452
Traes_3B_02B5D826F.1	heat shock protein 101, putative, expressed;0.0;872
Traes_3B_0DFD83BB0.1	receptor-like protein kinase precursor, putative, expressed;0.0;1233
Traes_3B_1C2CC2737.1	expressed protein;2e-171;600
Traes_3B_1DE4243CF.1	WD domain, G-beta repeat domain containing protein, expressed;4e-76;281
Traes_3B_204F59612.1	importin-alpha re-exporter, putative, expressed;0.0;1225
Traes_3B_240E7DC49.2	expressed protein;3e-156;550
Traes_3B_27823CD65.1	expressed protein;4e-29;125
Traes_3B_3007A0B49.1	plastocyanin-like domain containing protein, putative, expressed;2e-65;246
Traes_3B_36B347970.1	ion channel DMI1, chloroplast precursor, putative, expressed;0.0;1247
Traes_3B_3F6764629.2	wax synthase, putative, expressed;1e-147;521
Traes_3B_422A54CF6.1	leucoanthocyanidin dioxygenase, putative, expressed;3e-118;422
Traes_3B_4745DEECD.1	aquaporin protein, putative, expressed;1e-60;229
Traes_3B_4A757460F.1	cytochrome P450, putative, expressed;0.0;728
Traes_3B_4D8D01EDC.1	AP2 domain containing protein, expressed;3e-28;122
Traes_3B_52F9AF137.1	phosphoesterase family protein, putative, expressed;1e-78;290
Traes_3B_5CE03374D.1	ATP binding protein, putative, expressed;0.0;852
Traes_3B_6F94B79ED.1	expressed protein;2e-80;296
Traes_3B_6FF962479.6	pentatricopeptide, putative, expressed;0.0;1764
Traes_3B_728668685.1	glycosyl hydrolases family 17, putative, expressed;3e-113;406
Traes_3B_783145518.1	outer membrane protein, OMP85 family protein, expressed;5e-55;211
Traes_3B_7FA6E49BC.2	transcriptional corepressor LEUNIG, putative, expressed;0.0;783
Traes_3B_814B63B3B.1	sulfotransferase domain containing protein, expressed;2e-121;432
Traes_3B_89B839481.1	zinc finger family protein, putative, expressed;1e-19;94.4
Traes_3B_91C0A4F75.1	glucosidase II beta subunit-like domain containing protein, expressed;1e-158;557
Traes_3B_987C3D183.2	retrotransposon protein, putative, Ty1-copia subclass, expressed;0.0;1677
Traes_3B_9F2AF0F06.2	expressed protein;6e-44;175
Traes_3B_A06D9FE4E.1	pentatricopeptide repeat protein PPR1106-17, putative, expressed;0.0;912
Traes_3B_A0CFD8B09.1	cytochrome P450, putative, expressed;0.0;708
Traes_3B_A53AE2708.2	NAD binding domain of 6-phosphogluconate dehydrogenase containing protein, expressed;3e-128;456
Traes_3B_A62194A70.1	phosphoesterase family protein, putative, expressed;9e-78;287
Traes_3B_A8D8007CE.3	PPR repeat domain containing protein, putative, expressed;0.0;793
Traes_3B_AAA567A7C.2	Sad1 / UNC-like C-terminal domain containing protein, putative, expressed;3e-79;293

Traes_3B_ACA0B2FE3.2	embryo-specific 3, putative, expressed;1e-55;213
Traes_3B_B018E0E3F.1	antigen peptide transporter-like 1, chloroplast precursor, putative, expressed;7e-149;525
Traes_3B_B0B39F08E.2	XIK, putative, expressed;0.0;909
Traes_3B_BB678EDC9.2	mitochondrial-processing peptidase subunit, mitochondrial precursor, putative, expressed;0.0;746
Traes_3B_C3CA81F1A.1	OsFBK23 - F-box domain and kelch repeat containing protein, expressed;0.0;701
Traes_3B_C6D1C9CD6.1	histone-lysine N-methyltransferase SUVR3, putative, expressed;1e-33;140
Traes_3B_D214CA621.2	expressed protein;6e-151;532
Traes_3B_D4422DD53.2	DDT domain containing protein, putative, expressed;0.0;1852
Traes_3B_D8913A925.1	sulfotransferase domain containing protein, expressed;4e-58;220
Traes_3B_D8913A9251.1	sulfotransferase domain containing protein, expressed;4e-58;220
Traes_3B_DABF51922.2	n/a
Traes_3B_DC47DEA94.2	GHMP kinases ATP-binding protein, putative, expressed;4e-150;529
Traes_3B_E041382C5.2	powdery mildew resistance protein PM3b, putative, expressed;0.0;984
Traes_3B_E613D74C7.2	galactosyltransferase, putative, expressed;4e-165;578
Traes_3B_F05B72B99.1	alpha-1,4-glucan-protein synthase, putative, expressed;0.0;666
Traes_3B_F68129CD7.2	sulfotransferase domain containing protein, expressed;7e-120;427
Traes_3DL_0253F1D11.1	antigen peptide transporter-like 1, chloroplast precursor, putative, expressed;2e-148;524
Traes_3DL_17927771F.1	BTBM1 - Bric-a-Brac, Tramtrack, Broad Complex BTB domain with Meprin and TRAF Homology MATH domain, expres
Traes_3DL_29E0EDEE5.2	pentatricopeptide repeat protein PPR1106-17, putative, expressed;6e-116;414
Traes_3DL_2BE060085.1	outer mitochondrial membrane porin, putative, expressed;1e-118;423
Traes_3DL_3EF10D41F.1	expressed protein;0.0;902
Traes_3DL_4A579C5AE.2	GHMP kinases ATP-binding protein, putative, expressed;1e-139;493
Traes_3DL_4C13F598C.1	transposon protein, putative, unclassified, expressed;2e-93;340
Traes_3DL_550A28DCA.1	WD domain, G-beta repeat domain containing protein, expressed;4e-76;281
Traes_3DL_70DB0CBC9.1	expressed protein;7e-55;196
Traes_3DL_8B8C61B5C.2	galactosyltransferase, putative, expressed;6e-40;159
Traes_3DL_92202CE15.1	MBTB21 - Bric-a-Brac, Tramtrack, Broad Complex BTB domain with Meprin and TRAF Homology MATH domain, expre
Traes_3DL_9D99D436E.6	n/a
Traes_3DL_9F783D300.1	mitochondrial-processing peptidase subunit, mitochondrial precursor, putative, expressed;0.0;658
Traes_3DL_A2127ABF7.1	alpha-1,4-glucan-protein synthase, putative, expressed;3e-16;81.3
Traes_3DL_B1C067FC9.1	expressed protein;0.0;735
Traes_3DL_B3425E966.2	SET domain protein SDG117, putative, expressed;6e-12;67.0
Traes_3DL_B9B059069.1	expressed protein;9e-14;73.2
Traes_3DL_ECC7B3DDC.2	transcriptional corepressor LEUNIG, putative, expressed;3e-133;473
Traes_3DS_072E3F246.2	pentatricopeptide, putative, expressed;0.0;1667
Traes_3DS_1A8DD29C0.1	cytochrome b6, putative, expressed;4e-19;91.7
Traes_3DS_47560A18D.1	n/a
Traes_3DS_ACCBCF727.1	importin-alpha re-exporter, putative, expressed;4e-146;514
Traes_3DS_E60A79C15.3	exostosin family protein, putative, expressed;6e-112;400
Traes_3DS_E843BD60D.1	glucosidase II beta subunit-like domain containing protein, expressed;7e-31;129
Traes_4AL_08650B7FC.1	expressed protein;2e-08;55.5
Traes_4AL_197CAE419.1	aberrant pollen transmission 1, putative, expressed;2e-139;493
Traes_4AL_19C21724F.2	protein transporter, putative, expressed;1e-154;544
Traes_4AL_2C949D942.1	PIII4 - Proteinase inhibitor II family protein precursor, expressed;1e-16;82.4
Traes_4AL_2F2190057.1	cytokinin-O-glucosyltransferase 2, putative, expressed;1e-166;408
Traes_4AL_3C5D2E722.2	spotted leaf 11, putative, expressed;4e-90;328
Traes_4AL_4B411B3A7.1	spermidine synthase, putative, expressed;4e-151;532
Traes_4AL_5AA1DFD55.1	retinol dehydrogenase, putative, expressed;1e-119;427
Traes_4AL_5B5B861D1.1	D-mannose binding lectin family protein, expressed;0.0;1028
Traes_4AL_60DF5384D.1	expressed protein;2e-100;362
Traes_4AL_6BCC55248.1	ubiquitin carboxyl-terminal hydrolase domain containing protein, expressed;0.0;862
Traes_4AL_77A43AE68.2	Cupin domain containing protein, expressed;3e-54;208
Traes_4AL_84D1591DF.1	ankyrin repeat family protein, putative, expressed;9e-69;258
Traes_4AL_884BA5746.1	nodulation-signaling pathway 2 protein, putative, expressed;1e-135;481
Traes_4AL_8B67440A2.1	1-phosphatidylinositol-4-phosphate 5-kinase/ zinc ion binding protein, putative, expressed;0.0;1640
Traes_4AL_A5B9F7B36.1	aminotransferase, classes I and II, domain containing protein, expressed;0.0;644
Traes_4AL_B0ABF8863.2	expressed protein;9e-75;278
Traes_4AL_BE200647B.1	cyclin, putative, expressed;3e-129;369
Traes_4AL_D48452758.1	TATA-binding protein, putative, expressed;8e-97;350
Traes_4AL_E8C519202.1	cytochrome b6, putative, expressed;2e-10;62.0
Traes_4AL_E9E72DEE2.1	subtilisin N-terminal Region family protein, expressed;2e-34;141
Traes_4AL_F75EDC0121.1	expressed protein;8e-102;368
Traes_4AL_F85A4543B.1	Cupin domain containing protein, expressed;4e-69;258
Traes_4AL_F9DCE24F4.1	fatty acid desaturase, putative, expressed;0.0;647

Traes_4AL_FA94601FA.1	nodulin, putative, expressed;2e-63;239
Traes_4AL_FE0AC7213.2	RNA polymerase III RPC4 domain containing protein, expressed;1e-67;254
Traes_4AL_FF379258F.1	auxin efflux carrier component, putative, expressed;2e-67;252
Traes_4AS_02DDB73FF.1	CUE domain containing protein, expressed;2e-120;431
Traes_4AS_197CAE419.1	aberrant pollen transmission 1, putative, expressed;2e-139;493
Traes_4AS_3075D8D45.1	expressed protein;7e-45;177
Traes_4AS_4B47AEE0D.2	KIP1, putative, expressed;0.0;1006
Traes_4AS_547AD04C7.2	expressed protein;4e-98;355
Traes_4AS_56B8F305C.1	expressed protein;0.0;816
Traes_4AS_58DE58B00.1	flap endonuclease, putative, expressed;0.0;1624
Traes_4AS_58DE58B001.1	flap endonuclease, putative, expressed;0.0;1624
Traes_4AS_5F95A9C48.2	OsSub27 - Putative Subtilisin homologue, expressed;0.0;1103
Traes_4AS_6740A0426.2	n/a
Traes_4AS_6F3D0F407.1	DNAJ heat shock N-terminal domain-containing protein, putative, expressed;0.0;706
Traes_4AS_8E594F267.1	tetratricopeptide repeat domain containing protein, expressed;5e-111;398
Traes_4AS_9089F11EC.1	RNase L inhibitor protein-related, putative, expressed;5e-101;365
Traes_4AS_92067319F.1	no apical meristem protein, putative, expressed;0.0;720
Traes_4AS_B59D0F85B.1	mannose-1-phosphate guanyltransferase, putative, expressed;0.0;665
Traes_4AS_C3BC395FC.1	OsSCP11 - Putative Serine Carboxypeptidase homologue, expressed;4e-177;619
Traes_4AS_D7511DC6D.1	transmembrane amino acid transporter protein, putative, expressed;0.0;898
Traes_4AS_D763D08C2.2	eukaryotic translation initiation factor, putative, expressed;4e-55;211
Traes_4AS_E5556AFAD.1	DEAD-box ATP-dependent RNA helicase, putative, expressed;0.0;1100
Traes_4AS_E8C519202.1	cytochrome b6, putative, expressed;2e-10;62.0
Traes_4AS_F0BE29CA6.2	receptor-like protein kinase precursor, putative, expressed;0.0;1494
Traes_4BL_0726DD6EC.1	tetratricopeptide repeat domain containing protein, expressed;1e-109;394
Traes_4BL_12097D3B9.1	OsSCP11 - Putative Serine Carboxypeptidase homologue, expressed;5e-135;478
Traes_4BL_14F4067BA.1	cytochrome b6, putative, expressed;4e-31;133
Traes_4BL_1C4500370.1	DNAJ heat shock N-terminal domain-containing protein, putative, expressed;4e-173;605
Traes_4BL_2136196AE.2	no apical meristem protein, putative, expressed;0.0;716
Traes_4BL_3A5910CB1.2	3-5 exonuclease family protein, putative, expressed;1e-44;178
Traes_4BL_455C9E392.1	mannose-1-phosphate guanyltransferase, putative, expressed;0.0;666
Traes_4BL_63129F6EA.1	OsSub27 - Putative Subtilisin homologue, expressed;2e-80;296
Traes_4BL_69CCE3A33.1	expressed protein;0.0;815
Traes_4BL_84545B3BC.2	CUE domain containing protein, expressed;0.0;992
Traes_4BL_96D26EBE3.2	eukaryotic translation initiation factor, putative, expressed;4e-55;211
Traes_4BL_AB6EA9652.1	cyclin-dependent kinase A-1, putative, expressed;1e-112;402
Traes_4BL_B25D04B2A.1	expressed protein;3e-46;182
Traes_4BL_BBD90FBEE.1	expressed protein;4e-54;207
Traes_4BL_CE91DFE45.1	60S ribosomal protein L18-3, putative, expressed;8e-96;347
Traes_4BL_D9E97AA3B.1	transmembrane amino acid transporter protein, putative, expressed;6e-103;370
Traes_4BL_E8209275B.1	cytochrome P450 protein, putative, expressed;4e-06;47.4
Traes_4BS_14F4067BA.1	cytochrome b6, putative, expressed;4e-31;133
Traes_4BS_4DA228A0D.4	Cupin domain containing protein, expressed;1e-65;247
Traes_4BS_4E68C8E47.1	cupin domain containing protein, expressed;2e-123;441
Traes_4BS_5930EF5D6.1	aminotransferase, classes I and II, domain containing protein, expressed;0.0;798
Traes_4BS_5E12F0B27.1	AGC_AGC_other_GWLd.2 - ACG kinases include homologs to PKA, PKG and PKC, expressed;0.0;1837
Traes_4BS_6067314D4.2	HEAT repeat family protein, putative, expressed;0.0;1194
Traes_4BS_77C3CDB1D.1	zinc finger protein-related, putative, expressed;5e-142;502
Traes_4BS_9D72D851C.1	dirigent, putative, expressed;9e-66;247
Traes_4BS_ACBE893D5.1	phytanoyl-CoA dioxygenase, putative, expressed;2e-133;472
Traes_4BS_B0651AAC8.1	1-phosphatidylinositol-4-phosphate 5-kinase/ zinc ion binding protein, putative, expressed;0.0;1647
Traes_4BS_D2F0025CE.2	expressed protein;3e-103;372
Traes_4DL_05BF7F181.1	expressed protein;3e-32;136
Traes_4DL_144856316.1	hsp20/alpha crystallin family protein, putative, expressed;1e-58;222
Traes_4DL_1603FCED3.2	DEAD-box ATP-dependent RNA helicase, putative, expressed;0.0;1111
Traes_4DL_20B81B9E9.1	3-5 exonuclease family protein, putative, expressed;2e-70;264
Traes_4DL_260D64264.1	cell cycle checkpoint protein RAD17, putative, expressed;0.0;639
Traes_4DL_27051274A.2	expressed protein;8e-102;367
Traes_4DL_28E7C238E.2	no apical meristem protein, putative, expressed;1e-107;386
Traes_4DL_2C636B5DB.2	KIP1, putative, expressed;0.0;1996
Traes_4DL_427175FD6.2	expressed protein;2e-169;593
Traes_4DL_4955B6A74.1	CUE domain containing protein, expressed;4e-05;44.3
Traes_4DL_4B08D7DF4.1	OsSCP11 - Putative Serine Carboxypeptidase homologue, expressed;4e-116;415
Traes_4DL_5138702FD.1	cytochrome P450 protein, putative, expressed;2e-09;58.5

Traes_4DL_562002F72.1	OsSub27 - Putative Subtilisin homologue, expressed;1e-137;487
Traes_4DL_66BE3C639.2	cytochrome P450, putative, expressed;0.0;655
Traes_4DL_69A2A1F13.1	modifier of rudimentary protein, expressed;1e-65;246
Traes_4DL_78705D128.3	expressed protein;2e-101;366
Traes_4DL_816A82380.1	receptor-like protein kinase precursor, putative, expressed;0.0;1504
Traes_4DL_9C6C9CB1A.1	14-3-3 protein, putative, expressed;6e-58;211
Traes_4DL_A8AE227C0.1	tetratricopeptide repeat domain containing protein, expressed;8e-112;401
Traes_4DL_BCA20D7B4.1	RNase L inhibitor protein-related, putative, expressed;3e-91;332
Traes_4DL_BF0B81D38.2	transmembrane amino acid transporter protein, putative, expressed;0.0;884
Traes_4DL_DE7774064.1	eukaryotic translation initiation factor, putative, expressed;4e-55;211
Traes_4DL_E2CE97697.1	1-phosphatidylinositol-4-phosphate 5-kinase/ zinc ion binding protein, putative, expressed;0.0;1652
Traes_4DL_F647D6077.1	zinc finger C-x8-C-x5-C-x3-H type family protein, expressed;1e-21;99.4
Traes_4DS_0B314A876.1	expressed protein;1e-44;177
Traes_4DS_1CF5D9107.1	fatty acid desaturase, putative, expressed;0.0;665
Traes_4DS_50A614BFB.1	CCT/B-box zinc finger protein, putative, expressed;1e-81;301
Traes_4DS_54CE7EA5E.1	expressed protein;0.0;867
Traes_4DS_5835552DF.1	expressed protein;2e-48;189
Traes_4DS_700213C0B.1	TATA-binding protein, putative, expressed;6e-104;374
Traes_4DS_998201C4C.3	expressed protein;2e-107;386
Traes_4DS_B329DED6D.1	cyclin, putative, expressed;3e-137;486
Traes_4DS_BB124F81F.1	aminotransferase, classes I and II, domain containing protein, expressed;0.0;784
Traes_4DS_BDB03ED9C.3	potassium channel protein, putative, expressed;1e-97;354
Traes_4DS_C2E603176.1	cupin domain containing protein, expressed;7e-130;462
Traes_4DS_D50FB82F2.1	oxygen evolving enhancer protein 3 domain containing protein, expressed;3e-42;169
Traes_4DS_D71562B14.1	Cupin domain containing protein, expressed;3e-68;255
Traes_4DS_D7894C5BF.1	Cupin domain containing protein, expressed;2e-67;253
Traes_4DS_F495922CB.2	Cupin domain containing protein, expressed;3e-66;249
Traes_4DS_FF5323EAC.1	dirigent, putative, expressed;3e-65;245
Traes_5AL_06DC5CDA7.1	AP2 domain containing protein, expressed;4e-39;158
Traes_5AL_0E7E66066.1	mTERF family protein, expressed;8e-24;106
Traes_5AL_12B7B7A3B.1	retrotransposon protein, putative, unclassified, expressed;1e-16;83.6
Traes_5AL_1A119E73D.1	WD domain, G-beta repeat domain containing protein, expressed;0.0;1365
Traes_5AL_20D235B9F.1	pentatricopeptide, putative, expressed;2e-138;490
Traes_5AL_2A40F0298.1	AGC_PVPK_like_kin82y.16 - ACG kinases include homologs to PKA, PKG and PKC, expressed;0.0;655
Traes_5AL_3618D7967.1	expressed protein;0.0;838
Traes_5AL_588A7DB54.1	protein kinase, putative, expressed;3e-17;84.7
Traes_5AL_832CDADBB.1	OsSCP44 - Putative Serine Carboxypeptidase homologue, expressed;3e-169;592
Traes_5AL_984F155C1.2	PHLOEM 2-LIKE A10, putative, expressed;1e-39;157
Traes_5AL_98521B264.1	transcription factor, putative, expressed;3e-10;61.2
Traes_5AL_A3A857B99.2	expressed protein;8e-66;247
Traes_5AL_BB0405CD3.1	pollen signalling protein with adenylyl cyclase activity, putative, expressed;0.0;903
Traes_5AL_E8D0BB87B.3	NAD dependent epimerase/dehydratase family protein, putative, expressed;0.0;729
Traes_5AL_EE3A00A12.2	cyclin-dependent kinase A-1, putative, expressed;4e-118;421
Traes_5AL_F6064A23A.2	expressed protein;0.0;1113
Traes_5AS_94ACA1538.1	cytochrome b6, putative, expressed;3e-28;123
Traes_5BL_028D02DF6.1	homeobox associated leucine zipper, putative, expressed;3e-44;176
Traes_5BL_03D637203.1	cytochrome P450, putative, expressed;2e-145;514
Traes_5BL_0D3F2CF69.2	Ser/Thr protein phosphatase family protein, putative, expressed;3e-158;556
Traes_5BL_111B47D32.1	PHLOEM 2-LIKE A10, putative, expressed;7e-121;431
Traes_5BL_1FC1BC551.1	oxidoreductase, short chain dehydrogenase/reductase family domain containing protein, expressed;8e-130;461
Traes_5BL_3D2A3CDD6.1	transcription initiation factor IIB, putative, expressed;0.0;708
Traes_5BL_3EDC2B2CE.1	lissencephaly type-1-like homology motif, putative, expressed;0.0;676
Traes_5BL_4332A094A.1	ubiquitin family protein, putative, expressed;6e-73;272
Traes_5BL_4332A094A1.1	ubiquitin family protein, putative, expressed;6e-73;272
Traes_5BL_545CD9F8B1.1	mitochondrial carrier protein, putative, expressed;1e-122;437
Traes_5BL_5C2E1C7F8.2	OsSAUR40 - Auxin-responsive SAUR gene family member, expressed;5e-55;210
Traes_5BL_5CE389C1A.2	cytochrome P450, putative, expressed;2e-83;305
Traes_5BL_5F432CEF9.1	UDP-glucoronosyl and UDP-glucosyl transferase domain containing protein, expressed;2e-116;407
Traes_5BL_625657BE4.1	cytochrome P450, putative, expressed;1e-110;397
Traes_5BL_63DA0055E.2	expressed protein;4e-56;216
Traes_5BL_68BDA8BC7.1	pirin, putative, expressed;3e-143;505
Traes_5BL_691C79CF8.1	stress-induced protein, putative, expressed;8e-72;209
Traes_5BL_6B0820536.1	IBR domain containing protein, expressed;9e-135;478
Traes_5BL_6F5717D80.1	IscA-like iron-sulfur assembly protein, mitochondrial precursor, putative, expressed;5e-48;187

Traes_5BL_7444BC60E.1	pollen signalling protein with adenylyl cyclase activity, putative, expressed;0.0;936
Traes_5BL_7FB30012A.1	PPR repeat domain containing protein, putative, expressed;0.0;1191
Traes_5BL_8512C24F7.1	periplasmic beta-glucosidase precursor, putative, expressed;0.0;890
Traes_5BL_902F46492.1	ZOS5-12 - C2H2 zinc finger protein, expressed;2e-23;105
Traes_5BL_90FC7AB89.1	OsFBDUF47 - F-box and DUF domain containing protein, expressed;0.0;785
Traes_5BL_94C5B581B.1	expressed protein;1e-97;355
Traes_5BL_9A8443C38.1	reticuline oxidase-like protein precursor, putative, expressed;9e-162;568
Traes_5BL_B58E08F2F.1	OsSub57 - Putative Subtilisin homologue, expressed;0.0;1145
Traes_5BL_B724A4311.1	peptidyl-prolyl cis-trans isomerase, putative, expressed;7e-104;374
Traes_5BL_C05A786BB.1	NAD dependent epimerase/dehydratase family protein, putative, expressed;0.0;731
Traes_5BL_C08428870.1	transcription factor, putative, expressed;6e-175;612
Traes_5BL_C4CA0822B.1	Cupin domain containing protein, expressed;7e-70;261
Traes_5BL_C79594291.1	mTERF family protein, expressed;3e-102;369
Traes_5BL_C99C99B3C.1	expressed protein;0.0;1092
Traes_5BL_CD6CD078C.1	leucine-rich repeat receptor protein kinase EXS precursor, putative, expressed;2e-18;57.8
Traes_5BL_D3FF5C8C9.1	expressed protein;2e-46;181
Traes_5BL_DFFA40472.1	D-mannose binding lectin family protein, expressed;0.0;1054
Traes_5BL_E0C2C7D72.2	leaf senescence related protein, putative, expressed;0.0;748
Traes_5BL_E48CF8316.1	Cupin domain containing protein, expressed;1e-68;257
Traes_5BL_ECA7A48BC.2	expressed protein;2e-47;185
Traes_5BL_EF54ABD85.1	OsSub7 - Putative Subtilisin homologue, expressed;1e-128;457
Traes_5BL_F51D3D269.1	crcB-like protein, expressed;4e-21;97.4
Traes_5BL_F8A34CF5A.1	HLS, putative, expressed;1e-104;377
Traes_5BL_FC6D62A99.1	oxidoreductase, short chain dehydrogenase/reductase family domain containing protein, expressed;2e-77;286
Traes_5BS_2C2072466.1	Glutaredoxin, thioredoxin-like superfamily
Traes_5BS_3DA538381.2	chalcone and stilbene synthases, putative, expressed;6e-180;628
Traes_5BS_409B24307.1	Glutaredoxin, thioredoxin-like superfamily
Traes_5BS_685364484.1	reticulon domain containing protein, putative, expressed;7e-95;345
Traes_5BS_742D62174.1	expressed protein;2e-69;259
Traes_5BS_95A52A87F.2	MYB family transcription factor, putative, expressed;2e-93;340
Traes_5BS_9A5DEAB72.1	galactosyltransferase family protein, putative, expressed;0.0;919
Traes_5BS_AE808B2D5.1	DNA binding protein, putative, expressed;8e-132;468
Traes_5BS_D58B83EFC.1	carbohydrate binding protein, putative, expressed;0.0;980
Traes_5BS_E2116F66A.1	OsFBDUF17 - F-box and DUF domain containing protein, expressed;2e-124;398
Traes_5BS_EA45D02B6.1	cytochrome b6, putative, expressed;4e-26;115
Traes_5BS_EA45D02B61.1	cytochrome b6, putative, expressed;4e-26;115
Traes_5BS_EA45D02B62.1	cytochrome b6, putative, expressed;4e-26;115
Traes_5DL_02477C27A.1	mTERF family protein, expressed;1e-94;344
Traes_5DL_029F13A75.1	n/a
Traes_5DL_1950C1FC2.2	transcription factor, putative, expressed;2e-173;606
Traes_5DL_23CBF391D.2	CAAX amino terminal protease family protein, putative, expressed;4e-122;438
Traes_5DL_25F045F32.1	IscA-like iron-sulfur assembly protein, mitochondrial precursor, putative, expressed;4e-49;191
Traes_5DL_294CF0C3A.1	retrotransposon protein, putative, unclassified, expressed;9e-17;84.0
Traes_5DL_488FBAEEC.2	LTPL14 - Protease inhibitor/seed storage/LTP family protein precursor, expressed;2e-30;129
Traes_5DL_4A82A1EB5.1	leaf senescence related protein, putative, expressed;4e-161;565
Traes_5DL_5B7A127BA.1	Cupin domain containing protein, expressed;6e-72;268
Traes_5DL_5F97D4DAB.1	n/a
Traes_5DL_6669993AB.1	oxidoreductase, short chain dehydrogenase/reductase family domain containing protein, expressed;3e-125;446
Traes_5DL_7156326D7.1	NAD dependent epimerase/dehydratase family protein, putative, expressed;0.0;729
Traes_5DL_795A597CD.1	cytochrome b6, putative, expressed;4e-31;133
Traes_5DL_85A7A16F4.1	OsFBDUF47 - F-box and DUF domain containing protein, expressed;0.0;795
Traes_5DL_85C6DAE2F.2	expressed protein;2e-60;230
Traes_5DL_91EFA6E79.1	D-mannose binding lectin family protein, expressed;0.0;1033
Traes_5DL_9819A5F4A.1	expressed protein;7e-28;119
Traes_5DL_A6EC2A121.1	amino acid permease family protein, putative, expressed;1e-160;564
Traes_5DL_A95A73FFE.2	transcription initiation factor IIB, putative, expressed;6e-157;551
Traes_5DL_BC501B3FC.1	peptidyl-prolyl cis-trans isomerase, putative, expressed;7e-104;374
Traes_5DL_BE544F15F.1	expressed protein;6e-61;231
Traes_5DL_D897DFC04.1	cytochrome P450, putative, expressed;2e-57;221
Traes_5DL_DC720C4C0.2	expressed protein;7e-66;247
Traes_5DL_E86CFDE67.1	Ser/Thr protein phosphatase family protein, putative, expressed;0.0;703
Traes_5DL_E98838ED9.1	response regulator receiver domain containing protein, expressed;1e-25;112
Traes_5DL_F0D3764AE.1	expressed protein;5e-23;104
Traes_5DL_F2A8D27D3.1	GDP-mannose transporter, putative, expressed;2e-178;623

Traes_5DS_1DD7E13CF.2	cytochrome P450, putative, expressed;2e-29;125
Traes_5DS_286CDF1FE1.2	Glutaredoxin, thioredoxin-like superfamily
Traes_5DS_6CBA32EA1.1	WD-40 repeat family protein, putative, expressed;9e-166;581
Traes_5DS_8D8699156.2	reticulon domain containing protein, putative, expressed;3e-101;366
Traes_5DS_A4147A90B.1	galactosyltransferase family protein, putative, expressed;0.0;801
Traes_5DS_AEC533CD9.1	chalcone and stilbene synthases, putative, expressed;0.0;640
Traes_5DS_B0BE4E0F9.2	cytochrome P450, putative, expressed;0.0;655
Traes_5DS_FC6EAC840.1	cytochrome b6, putative, expressed;2e-28;122
Traes_5DS_FD0D30583.1	invertase/pectin methylesterase inhibitor family protein, putative, expressed;8e-43;171
Traes_6AL_34FD736F3.1	transporter, major facilitator family, putative, expressed;0.0;939
Traes_6AL_391580249.1	AT hook motif family protein, expressed;5e-174;609
Traes_6AL_451D27E5E.1	fasciclin-like arabinogalactan precursor protein, putative, expressed;5e-24;107
Traes_6AL_51C78D35E.2	expressed protein;3e-50;194
Traes_6AL_5E2427983.1	ABC transporter, ATP-binding protein, putative, expressed;0.0;1098
Traes_6AL_6C8740229.1	dynamin, putative, expressed;0.0;1041
Traes_6AL_8262324DA.1	OsSCP10 - Putative Serine Carboxypeptidase homologue, expressed;1e-160;563
Traes_6AL_8BF77066D.3	resistance protein LR10, putative, expressed;0.0;824
Traes_6AL_A923A5347.1	oligopeptidase, putative, expressed;0.0;1222
Traes_6AL_ADC78CBC4.1	pentatricopeptide, putative, expressed;7e-89;325
Traes_6AL_EA45D02B6.1	cytochrome b6, putative, expressed;4e-26;115
Traes_6AL_F099F366F.1	phosphoribosyl transferase, putative, expressed;1e-167;587
Traes_6AL_F46F74A23.1	galactosyltransferase family protein, putative, expressed;0.0;893
Traes_6AS_07A9CA267.2	plant neutral invertase domain containing protein, expressed;0.0;921
Traes_6AS_11466A986.2	pollen signalling protein with adenylyl cyclase activity, putative, expressed;0.0;940
Traes_6AS_14F4067BA.1	cytochrome b6, putative, expressed;4e-31;133
Traes_6AS_21AF25515.2	ATA15 protein, putative, expressed;3e-93;339
Traes_6AS_24C6E88D9.1	retrotransposon protein, putative, unclassified, expressed;5e-44;174
Traes_6AS_4D5775944.1	helix-loop-helix DNA-binding domain containing protein, expressed;4e-94;343
Traes_6AS_66DA47CC7.1	alpha-1,4-glucan-protein synthase, putative, expressed;2e-60;105
Traes_6AS_712D46544.1	SNF2 family N-terminal domain containing protein, expressed;0.0;1436
Traes_6AS_760F5B9E1.1	alpha-1,4-glucan-protein synthase, putative, expressed;2e-58;105
Traes_6AS_799DE5651.1	alpha-1,4-glucan-protein synthase, putative, expressed;6e-41;98.2
Traes_6AS_799DE56511.1	alpha-1,4-glucan-protein synthase, putative, expressed;1e-41;98.2
Traes_6AS_799DE56512.1	alpha-1,4-glucan-protein synthase, putative, expressed;1e-41;98.2
Traes_6AS_8AE59E7D9.1	OsSub10 - Putative Subtilisin homologue, expressed;0.0;884
Traes_6AS_9596E4B58.1	E2F family transcription factor protein, putative, expressed;3e-180;629
Traes_6AS_A4768153B.1	glycosyl hydrolases family 17, putative, expressed;2e-162;570
Traes_6AS_D4A88E320.1	expressed protein;2e-05;45.1
Traes_6AS_DDB21F262.1	pollen signalling protein with adenylyl cyclase activity, putative, expressed;2e-152;537
Traes_6BL_14F4067BA.1	cytochrome b6, putative, expressed;4e-31;133
Traes_6BL_55882F049.3	cyclin G-associated kinase, putative, expressed;0.0;1103
Traes_6BL_5837C5783.2	ATA15 protein, putative, expressed;1e-93;340
Traes_6BL_65BEAB58C.2	aldehyde dehydrogenase, putative, expressed;0.0;777
Traes_6BL_BE1824E98.2	cadmium tolerance factor, putative, expressed;2e-143;464
Traes_6BL_C1AC5C2F6.1	OsWAK22 - OsWAK receptor-like protein kinase, expressed;8e-29;122
Traes_6BL_E2F8DAB34.2	ceramide kinase-related, putative, expressed;5e-162;569
Traes_6BL_E486D0E41.2	glucose-6-phosphate 1-dehydrogenase, cytoplasmic isoform, putative, expressed;0.0;778
Traes_6BL_E5D5A61DA.1	vignain precursor, putative, expressed;2e-162;569
Traes_6BL_FAD2FD89C.1	transporter, major facilitator family, putative, expressed;0.0;848
Traes_6BL_FE09F7466.1	selenium-binding protein-like, putative, expressed;7e-89;325
Traes_6BS_1660E311F.1	potassium transporter, putative, expressed;9e-23;102
Traes_6BS_21574D556.1	cytochrome b6, putative, expressed;6e-31;133
Traes_6BS_302BCA824.1	CGMC_MAPKCMGC_2.7 - CGMC includes CDA, MAPK, GSK3, and CLKC kinases, expressed;1e-95;347
Traes_6BS_5671DF531.1	wall-associated receptor kinase 3 precursor, putative, expressed;3e-111;399
Traes_6BS_73AF866E3.2	ubiquitin-conjugating enzyme, putative, expressed;2e-95;345
Traes_6BS_A1BCAA7ED.1	blight resistance protein T118, putative, expressed;0.0;672
Traes_6BS_A51045ADB.1	transporter, major facilitator family, putative, expressed;0.0;645
Traes_6BS_B844D6558.2	SNF2 family N-terminal domain containing protein, expressed;0.0;1306
Traes_6BS_BD894AD26.1	auxin response factor 6, putative, expressed;0.0;1447
Traes_6DL_057BC5C9A.1	serine/threonine-protein kinase receptor precursor, putative, expressed;4e-45;177
Traes_6DL_10926529D.1	dynamin, putative, expressed;0.0;1099
Traes_6DL_12CD9ACC0.1	glutamate receptor, putative, expressed;3e-84;309
Traes_6DL_14F4067BA.1	cytochrome b6, putative, expressed;4e-31;133
Traes_6DL_1B30D0116.1	fasciclin-like arabinogalactan precursor protein, putative, expressed;8e-61;231

Traes_6DL_1B77BC5531.2	n/a
Traes_6DL_1D288651B.1	pentatricopeptide, putative, expressed;4e-109;393
Traes_6DL_2A69F7528.1	retrotransposon protein, putative, unclassified, expressed;1e-21;99.4
Traes_6DL_315652308.2	ceramide kinase-related, putative, expressed;8e-142;502
Traes_6DL_3C5DF323B.2	vignain precursor, putative, expressed;2e-152;536
Traes_6DL_3ED45AC7F.1	phosphoribosyl transferase, putative, expressed;4e-149;525
Traes_6DL_5E7B1EA4D.1	expressed protein;1e-29;127
Traes_6DL_6EC763C47.1	peptide transporter PTR2, putative, expressed;3e-174;609
Traes_6DL_8B7AE5FF2.1	pectinesterase, putative, expressed;7e-157;551
Traes_6DL_92B3B176F.2	AT hook motif family protein, expressed;2e-176;617
Traes_6DL_C44F19089.1	transporter, major facilitator family, putative, expressed;0.0;901
Traes_6DL_E31AB6EED.1	alpha-amylase precursor, putative, expressed;0.0;704
Traes_6DL_FECA3FE13.1	AP2 domain containing protein, expressed;6e-98;355
Traes_6DS_03B231497.1	ubiquitin-conjugating enzyme, putative, expressed;2e-95;345
Traes_6DS_053061050.1	helix-loop-helix DNA-binding domain containing protein, expressed;6e-46;182
Traes_6DS_0AC635A1C.1	transporter, major facilitator family, putative, expressed;0.0;635
Traes_6DS_0AC635A1C1.1	transporter, major facilitator family, putative, expressed;0.0;635
Traes_6DS_185723D1E.1	STE_PAK_Ste20_STLK.1 - STE kinases include homologs to sterile 7, sterile 11 and sterile 20 from yeast, expressed;2e
Traes_6DS_5EE5BC9B3.1	GDSL-like lipase/acylhydrolase, putative, expressed;2e-140;496
Traes_6DS_6E31981FF.1	phytosulfokine receptor precursor, putative, expressed;2e-97;353
Traes_6DS_794C3DF2C.1	transporter, major facilitator family, putative, expressed;0.0;730
Traes_6DS_8D82C81DB.1	cytochrome b6, putative, expressed;4e-31;133
Traes_6DS_AA7F69F37.1	SNF2 family N-terminal domain containing protein, expressed;0.0;1433
Traes_6DS_F56597DB3.2	DUF581 domain containing protein, expressed;3e-49;192
Traes_7AL_094506A5A.1	zinc finger CCHC domain-containing protein 10, putative, expressed;1e-53;207
Traes_7AL_18EE69735.1	serine/threonine-protein kinase receptor precursor, putative, expressed;0.0;780
Traes_7AL_4BC00ED54.1	serine/threonine-protein kinase receptor precursor, putative, expressed;0.0;631
Traes_7AL_61C51C0CD.1	expressed protein;6e-71;265
Traes_7AL_7A2639A1B.2	copper-transporting ATPase, putative, expressed;0.0;1533
Traes_7AL_7E37FB227.1	cytochrome b6, putative, expressed;2e-31;133
Traes_7AL_C64F17133.2	OsWAK108 - OsWAK receptor-like protein kinase, expressed;0.0;664
Traes_7AL_CCBA8A129.1	phospholipase C, putative, expressed;5e-132;468
Traes_7AL_E3ADC8C38.1	auxin response factor 18, putative, expressed;1e-106;335
Traes_7AL_F05316203.2	vacuolar-sorting receptor precursor, putative, expressed;9e-48;186
Traes_7AL_F445E67BB.3	phosphatidylinositol-4-phosphate 5-Kinase, putative, expressed;0.0;2104
Traes_7AL_F8EB563ED.1	cytochrome P450, putative, expressed;0.0;647
Traes_7AS_0D3979FCB.1	DNA-repair protein complementing XP-C cells, putative, expressed;2e-84;308
Traes_7AS_10A9A261D.1	OsWAK11 - OsWAK receptor-like protein kinase, expressed;4e-14;73.9
Traes_7AS_13B014806.2	expressed protein;3e-23;108
Traes_7AS_1D7969D3E.1	60S ribosomal protein L39, putative, expressed;5e-14;73.9
Traes_7AS_24DCC6FA4.1	pentatricopeptide, putative, expressed;0.0;649
Traes_7AS_342973D401.5	phosphatidylinositol 3- and 4-kinase family protein, putative, expressed;0.0;804
Traes_7AS_3C1208141.1	bolA, putative, expressed;6e-33;136
Traes_7AS_7F104C1B7.1	transposon protein, putative, unclassified, expressed;2e-81;300
Traes_7AS_9C66A3714.1	coiled-coil domain containing 49, putative, expressed;7e-54;209
Traes_7AS_BC38CEEE2.1	citrate-binding protein precursor, putative, expressed;9e-92;333
Traes_7AS_BC38CEEE21.1	citrate-binding protein precursor, putative, expressed;9e-92;333
Traes_7AS_E28C6595A.2	DNA repair ATPase-related, putative, expressed;8e-171;597
Traes_7AS_F9C6EBD97.2	uvrB/uvrC motif family protein, expressed;8e-156;549
Traes_7BL_03FF808BF.1	transposable element protein, putative, containing Pfam profile: PF03004, Transposase_24, expressed;2e-111;400
Traes_7BL_0D3B2A96A.1	receptor-like kinase, putative, expressed;2e-46;181
Traes_7BL_0FABEE965.2	phosphatidylinositol-4-phosphate 5-Kinase, putative, expressed;0.0;832
Traes_7BL_18D335F08.2	auxin response factor 18, putative, expressed;2e-49;191
Traes_7BL_25381F605.1	expressed protein;7e-147;518
Traes_7BL_2C564E996.2	phospholipase C, putative, expressed;7e-133;471
Traes_7BL_34ECCF38F.1	cytochrome P450, putative, expressed;2e-68;256
Traes_7BL_5EB114AB7.1	cytochrome P450, putative, expressed;7e-73;270
Traes_7BL_69A11C4AF.1	WD repeat-containing protein, putative, expressed;0.0;1455
Traes_7BL_998EC9F74.2	MYB family transcription factor, putative, expressed;0.0;895
Traes_7BL_C5D2C17B3.1	transferase family protein, putative, expressed;4e-12;67.4
Traes_7BL_D6BB0C6DF.1	cytochrome b6, putative, expressed;7e-32;135
Traes_7BL_F9ECF4528.2	serine/threonine-protein kinase receptor precursor, putative, expressed;0.0;780
Traes_7BS_51B7DEC0E.1	expressed protein;1e-70;264
Traes_7BS_65EBEDEFC.1	expressed protein;1e-31;133

Traes_7BS_93A86D7A11.1	phosphatidylinositol 3- and 4-kinase family protein, putative, expressed;0.0;1007
Traes_7BS_955956707.1	SET domain containing protein, expressed;0.0;1177
Traes_7BS_9C61A9363.1	cytochrome b6, putative, expressed;2e-30;131
Traes_7BS_BFDFF014C.1	coiled-coil domain containing 49, putative, expressed;5e-56;216
Traes_7BS_C2D1B9700.2	uvrB/uvrC motif family protein, expressed;1e-128;457
Traes_7BS_CB15DB953.1	HECT-domain domain containing protein, expressed;2e-23;106
Traes_7DL_0C02D38AD.1	dehydrogenase, putative, expressed;2e-161;566
Traes_7DL_310E46F15.3	MYB family transcription factor, putative, expressed;0.0;916
Traes_7DL_3C03C77D4.1	expressed protein;0.0;797
Traes_7DL_55ADB3528.1	auxin response factor 18, putative, expressed;0.0;748
Traes_7DL_58184284F.2	pollen signalling protein with adenylyl cyclase activity, putative, expressed;0.0;977
Traes_7DL_66682FE93.1	zinc finger CCHC domain-containing protein 10, putative, expressed;1e-53;207
Traes_7DL_807EB60BD.1	phospholipase C, putative, expressed;6e-133;471
Traes_7DL_8DFE5AF5E.2	chromo domain-containing protein LHP1, putative, expressed;2e-75;281
Traes_7DL_96775548A.1	cytochrome b6, putative, expressed;4e-25;112
Traes_7DL_C203DF866.1	zinc finger protein, putative, expressed;2e-17;85.5
Traes_7DL_C5D3B7775.1	receptor-like kinase, putative, expressed;1e-95;347
Traes_7DL_FDD56DA11.2	phosphatidylinositol-4-phosphate 5-Kinase, putative, expressed;0.0;2087
Traes_7DS_0D1C03C27.1	SET domain containing protein, expressed;0.0;1186
Traes_7DS_219826521.2	uvrB/uvrC motif family protein, expressed;0.0;635
Traes_7DS_32EFB7CD0.2	pentatricopeptide, putative, expressed;0.0;694
Traes_7DS_3C83C8EF9.2	DNA repair ATPase-related, putative, expressed;4e-170;595
Traes_7DS_3D49B9F9B.2	60S ribosomal protein L39, putative, expressed;1e-24;109
Traes_7DS_4E9E23050.1	expressed protein;2e-53;206
Traes_7DS_53C6E8C4E.1	cytochrome b6, putative, expressed;8e-26;114
Traes_7DS_5DBF1BD6C.7	phosphatidylinositol 3- and 4-kinase family protein, putative, expressed;0.0;998
Traes_7DS_60A8AFEEF.1	OsWAK11 - OsWAK receptor-like protein kinase, expressed;3e-14;74.3
Traes_7DS_6B52B942F.2	DNA-repair protein complementing XP-C cells, putative, expressed;0.0;1021
Traes_7DS_6F5B0EBD8.1	expressed protein;2e-25;113
Traes_7DS_771C3A317.1	bolA, putative, expressed;7e-39;158
Traes_7DS_8B2BA1741.2	aldehyde dehydrogenase, putative, expressed;0.0;755
Traes_7DS_985883968.2	no apical meristem protein, putative, expressed;1e-76;285
Traes_7DS_98EE649A5.1	expressed protein;1e-31;132
Traes_7DS_AF579B6E8.2	ZOS8-11 - C2H2 zinc finger protein, expressed;0.0;677
Traes_7DS_EDF4F3552.1	citrate-binding protein precursor, putative, expressed;5e-75;278
Traes_7DS_F219C7147.2	peptidase, putative, expressed;0.0;464

Table S8. *Zymoseptoria tritici* (*Zt*) genes selected for gene silencing using BSMV-HIGS.

Target	Function	Expected phenotype	HIGS construct size* (in bp)	si-Fi software predictions (all siRNA hits *vs* effective siRNA hits)	si-Fi predicted off-target siRNA hits**
ZtCYP51 (construct M5)	Ergosterol biosynthesis	Lethal; fungicide target	343 bp	234 / 89	Zt: no<br>Wheat: no
ZtTUBa (construct M4)	Microtubules	Lethal or reduced virulence	281 bp	193 / 68	Zt: YES, to putative uncharacterised protein (siRNA hits - 61/22)<br>Wheat: no
ZtTUBb (construct M1)	Microtubules	Lethal or reduced virulence; fungicide target	303 bp	193 / 90	Zt: no<br>Wheat: no
ZtALG2 (construct M7)	Alpha-1,2-mannosyl-transferase	Loss of pathogenicity towards wheat	315 bp	209 / 86	Zt: no<br>Wheat: no

*size of protein coding sequences inserted into the BSMV vector (not including the LIC adaptor sequences)
**Tested for cross-hits against *Z. tritici* and wheat transcripts databases

Methods S1: Small RNA locus likelihood estimation.

Small RNA (sRNA) locus likelihood estimation is to provide a posterior probability value indicating the probability of a given locus to be a true small RNA locus. The posterior probability is based on the sRNA sequencing reads coverage depth and read length distribution information for a given locus. A true sRNA locus is assumed to have higher coverage than a null locus does, which may have some short RNA sequences aligned that originate for example from degradation of mRNA. In addition, sRNA locus usually has a dominant length reads, e.g. 21 nt or 24 nt reads are highly dominated in a sRNA locus compared to other lengths reads. On the other hand, a locus with random, degraded mRNA products usually displays a more uniform distribution of all types short length reads. The following method to estimate sRNA locus probability is based on the above two assumptions.

Bayesian model for sRNA locus prediction

Let *I_l~B(1, p), l=1,...L*, be the Bernoulli random variable indicating whether a locus *l* is a sRNA locus, *Prob(I_l)=p*, where:

$$I_l = \begin{cases} 0 & if\ I_l = 0 \\ 1 & if\ I_l = 1 \end{cases} \tag{1}$$

Then the posterior probability for a sRNA locus is:

$$\Pr(I_l = 1 \mid data) = \frac{p \Pr(data \mid I_l = 1)}{p \Pr(data \mid I_l = 1) + (1-p)\Pr(data \mid I_l = 0)} \tag{2}$$

The *Prob(data|I_l=1)* is based on both read coverage depth and read length distribution, therefore for locus *l*:

$$\Pr(data \mid I_l = 1) = \Pr(readcount_l \mid I_l = 1)\Pr(length - distribution_l \mid I_l = 1) \tag{3}$$

And *Prob(data|I_l=0)* for null locus *l:*

$$\Pr(data \mid I_l = 0) = \Pr(readcount_l \mid I_l = 0)\Pr(length - distribution_l \mid I_l = 0) \tag{4}$$

Negative binomial model for read count data

Negative binomial distribution is applied to model read count data. u_s and φ_s denotes the mean count and dispersion for sRNA loci, respectively; u_n an*d* φ_n denotes the mean count and dispersion for null loci, respectively. Therefore, for specific locus *l*, we have:

$$y_l \mid (u_s, \varphi_s, I_l = 1) \sim NB(u_s, \varphi_s)$$

$$y_l \mid (u_n, \varphi_n, I_l = 0) \sim NB(u_n, \varphi_n) \tag{5}$$

where y_j is a read count for a given locus *j*.

Finally, based on the negative binomial probability mass function, we have:

$$\Pr(readcount_l \mid I_l = 0) = P(Y = y_j \mid \mu_n, \varphi_n) = \frac{\Gamma(y + \varphi_n^{-1})}{\Gamma(\varphi_n^{-1})\Gamma(y+1)} \left(\frac{1}{1 + \mu_n \varphi_n}\right)^{\varphi^{-1}} \left(\frac{\mu_n}{\varphi_n^{-1} + \mu_n}\right)^{y}$$
